# An integrated data analysis reveals distribution, hosts, and pathogen diversity of *Haemaphysalis concinna*

**DOI:** 10.1186/s13071-024-06152-5

**Published:** 2024-02-27

**Authors:** Jing Liu, Xiao-Yu Han, Run-Ze Ye, Qing Xu, Xiao-Yang Wang, Ze-Hui Li, Yi Sun, Ke Song, Bao-Yu Wang, Shan-Shan Wang, Jin-Yue Liu, Lin Zhao, Wu-Chun Cao

**Affiliations:** 1 Institute of EcoHealth, School of Public Health, Cheeloo College of Medicine, Shandong University, 44 Wenhua Road, Lixia District, Jinan, 250012 People’s Republic of China; 2 State Key Laboratory of Pathogen and Biosecurity, Beijing Institute of Microbiology and Epidemiology, 20 Dong-da Street, Fengtai District, Beijing, 100071 People’s Republic of China

**Keywords:** *Haemaphysalis concinna*, Geographical distribution, Tick-borne disease, Avians, Ecological niche model

## Abstract

**Background:**

*Haemaphysalis concinna*, carrying multiple pathogens, has attracted increasing attention because of its expanded geographical range and significant role in disease transmission. This study aimed to identify the potential public health risks posed by *H. concinna* and *H. concinna*-associated pathogens.

**Methods:**

A comprehensive database integrating a field survey, literature review, reference book, and relevant websites was developed. The geographical distribution of *H. concinna* and its associated pathogens was illustrated using ArcGIS. Meta-analysis was performed to estimate the prevalence of *H. concinna*-associated microbes. Phylogenetic and geographical methods were used to investigate the role of birds in the transmission of *H. concinna*-associated microbes. The potential global distribution of *H. concinna* was predicted by ecological niche modeling.

**Results:**

*Haemaphysalis concinna* was distributed in 34 countries across the Eurasian continent, predominantly in China, Russia, and Central Europe. The tick species carried at least 40 human pathogens, including six species in the *Anaplasmataceae* family, five species of *Babesia*, four genospecies in the complex *Borrelia burgdorferi* sensu lato, ten species of spotted fever group rickettsiae, ten species of viruses, as well as *Francisella*, *Coxiella*, and other bacteria. *Haemaphysalis concinna* could parasitize 119 host species, with nearly half of them being birds, which played a crucial role in the long-distance transmission of tick-borne microbes. Our predictive modeling suggested that *H. concinna* could potentially survive in regions where the tick has never been previously recorded such as central North America, southern South America, southeast Oceania, and southern Africa.

**Conclusions:**

Our study revealed the wide distribution, broad host range, and pathogen diversity of *H. concinna*. Authorities, healthcare professionals, and the entire community should address the growing threat of *H. concinna* and associated pathogens. Tick monitoring and control, pathogen identification, diagnostic tools, and continuous research should be enhanced.

**Graphical Abstract:**

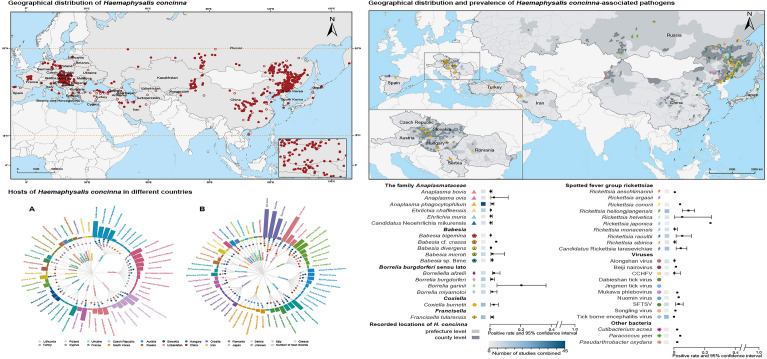

**Supplementary Information:**

The online version contains supplementary material available at 10.1186/s13071-024-06152-5.

## Background

*Haemaphysalis concinna*, a common vector in the Eurasian continent, plays a crucial role in transmitting various pathogens [1, 2]. In China alone, *H. concinna* has been reported to carry at least 22 different pathogens, including *Anaplasma phagocytophilum*, *Ehrlichia chaffeensis*, *Borrelia garinii*, *Babesia microti*, *Coxiella burnetii*, *Rickettsia raoultii*, and tick-borne encephalitis virus [1], posing a significant threat to human and animal health. China has experienced multiple outbreaks of *H. concinna*-borne diseases since the early twentieth century [3, 4], with one resulting in an unprecedented number of 126,843 human bites and causing a direct economic loss of approximately 3.45 million yuan (0.5 million $). Recent research indicates that the geographical range of *H. concinna* has expanded to southwest Poland and Lithuania, where this tick species has never been reported before [5, 6]. There is a growing concern that *H. concinna* has spread to new regions worldwide and could potentially introduce various pathogens to wider regions and populations.

*Haemaphysalis concinna* is reported to infest various animal hosts, especially avians [2, 7]. The long distances that avians span during migration may impact tick distribution and the spread of tick-borne microbes [8, 9]. Although previous research has suggested that birds could be important in spreading *Babesia* genotypes within *H. concinna* [10], evidence of the relationships between avian hosts and *H. concinna*-borne microbes remains limited. This study conducted a comprehensive analysis by integrating multiple global data sources to characterize the spatial distribution, associated pathogens, hosts, and potential habitats of *H. concinna*.

## Methods

### Data collection

By integrating multiple data sources, including a field survey, reference book, literature review, and related websites, we have extracted a comprehensive database detailing the distribution, associated pathogens, and hosts of *H. concinna*. The field survey was conducted by our Tick Genome and Microbiome Consortium from 2016 to 2021, covering 28 provinces, metropolises, or autonomous regions in mainland China. Ticks were identified to the species level by an entomologist. Distribution data of *H. concinna* were also collected from the reference book, Fauna Sinica-Arachnida Ixodida [11], the Global Biodiversity Information Facility (GBIF, https://www.gbif.org/), and GenBank (https://www.ncbi.nlm.nih.gov/genbank/).

Two independent reviewers searched for published articles from between January 1, 1959, and February 28, 2023, in electronic databases, including Web of Science, PubMed, China National Knowledge Infrastructure, and the WanFang database using the terms "*Haemaphysalis concinna*" and the Chinese name for *Haemaphysalis concinna*. Publications with the specified terms in any part of their content were identified. Articles were included for analysis if they met the following inclusion criteria: in English or Chinese, providing information on *H. concinna*’s location, associated microbes, or hosts. Articles lacking sufficient detail or duplicates were excluded. Additional file 1: Fig. S1 and Text S1 provide detailed descriptions of the literature search and data extraction.

Data on 24 environmental variables were collected to predict the global potential distribution of *H. concinna* (Additional file 2: Table S1). From the WorldClim database (http://www.worldclim.org), 19 bioclimatic variables (BIO1-BIO19) for 1970–2000 and elevation were downloaded. Slope and aspect variables were extracted from the Spatial Analyst Tool with ArcGIS (version 10.2; ESRI, Redlands, CA, USA). Percent tree cover and land cover were obtained from the Resource and Environmental Science and Data Center of Global Map (https://globalmaps.github.io/). All environmental variables were resampled at a layer resolution of 5 arc-min (~ 10 km).

Microbe sequences associated with *H. concinna* and those carried by birds were collected from GenBank. Sequences without geographic information were excluded from analysis. The species of avian hosts was identified from the literature review.

### Distribution of *H. concinna* and associated pathogens

The geographical distributions of *H. concinna* and its associated pathogens were mapped using ArcGIS. Latitude and longitude coordinates were utilized to denote the collection locations of *H. concinna*. In cases where the exact collection location was unavailable, the centroids of administrative regions were used instead.

### Estimation of pathogen prevalence

A meta-analysis was performed to estimate the combined positive rate and 95% confidence interval (CI) for each *H. concinna*-associated microbe. Studies that did not specify the number of ticks were excluded. When only one study identified a particular pathogen species, the positive rate was calculated as the number of positive ticks divided by the total number of ticks, without a 95% CI. However, when two or more studies were included, the combined positive rate and a 95% CI were estimated using R software (version 4.2.3, meta package). The *I*^2^ statistic was used to measure the variability of the data. *I*^2^ > 50% indicated that the heterogeneity was significant, and a random effects model was used. Otherwise, a fixed-effect model was utilized.

### Role of birds in the transmission of *H. concinna*-associated microbes

To assess the genetic evolutionary relationship between birds and tick-borne microbes of the same genus, phylogenetic methods were employed. Multiple sequence alignments were conducted using the L-INS-i algorithm in MAFFT (version 7.487). Ambiguously aligned regions were eliminated using TrimAl (version 1.4.rev15). The optimal nucleotide substitution model for each alignment was determined using the IQ-Tree (version 2.2.2.3) algorithm. Phylogenetic trees were constructed using the maximum likelihood method with 1000 bootstrap tests for reliability assessment. The resulting maximum-likelihood trees were visualized using FigTree (version 1.4.4).

Geographical analysis was conducted to examine the spatial correlation between *H. concinna* and avian species. Since there is no comprehensive database documenting the migration routes of migrant birds worldwide, only some bird species and regions that are important to *H. concinna* were focused on in this study. Nine bird migration routes in Europe were obtained from the Birds Migration Atlas website (https://migrationatlas.org/about) and three bird migration routes in China were derived from the Chinese National Geographic website (www.dili360.com).

### Prediction model of distribution of *H. concinna*

To predict the potential distribution of *H. concinna*, ecological niche modeling (ENM) was employed using the maximum entropy approach [12, 13]. A total of 243 actual locations (excluding administrative region centroids) were eventually included in the model construction process (Additional file 3: Table S2). Different combinations of variables, regularization multipliers (0–4), and feature classes (linear = L, quadratic = Q, product = P, threshold = T, and hinge = H) were examined to avoid spatial autocorrelation, collinearity, and overfitting [14–16]. The best-fitting model was selected based on the lower Akaike information criterion corrected (AICc) [17]. The final ENM was constructed using 25 replicates in Maxent software (version 3.4.1) with cross-validation. Extrapolation and clamping were turned off, and the model underwent a maximum of 10,000 iterations. Model performance was assessed by the area under the receiver-operating characteristic curve (AUC), which ranges from 0 to 1.

## Results

**Fig. 1:**
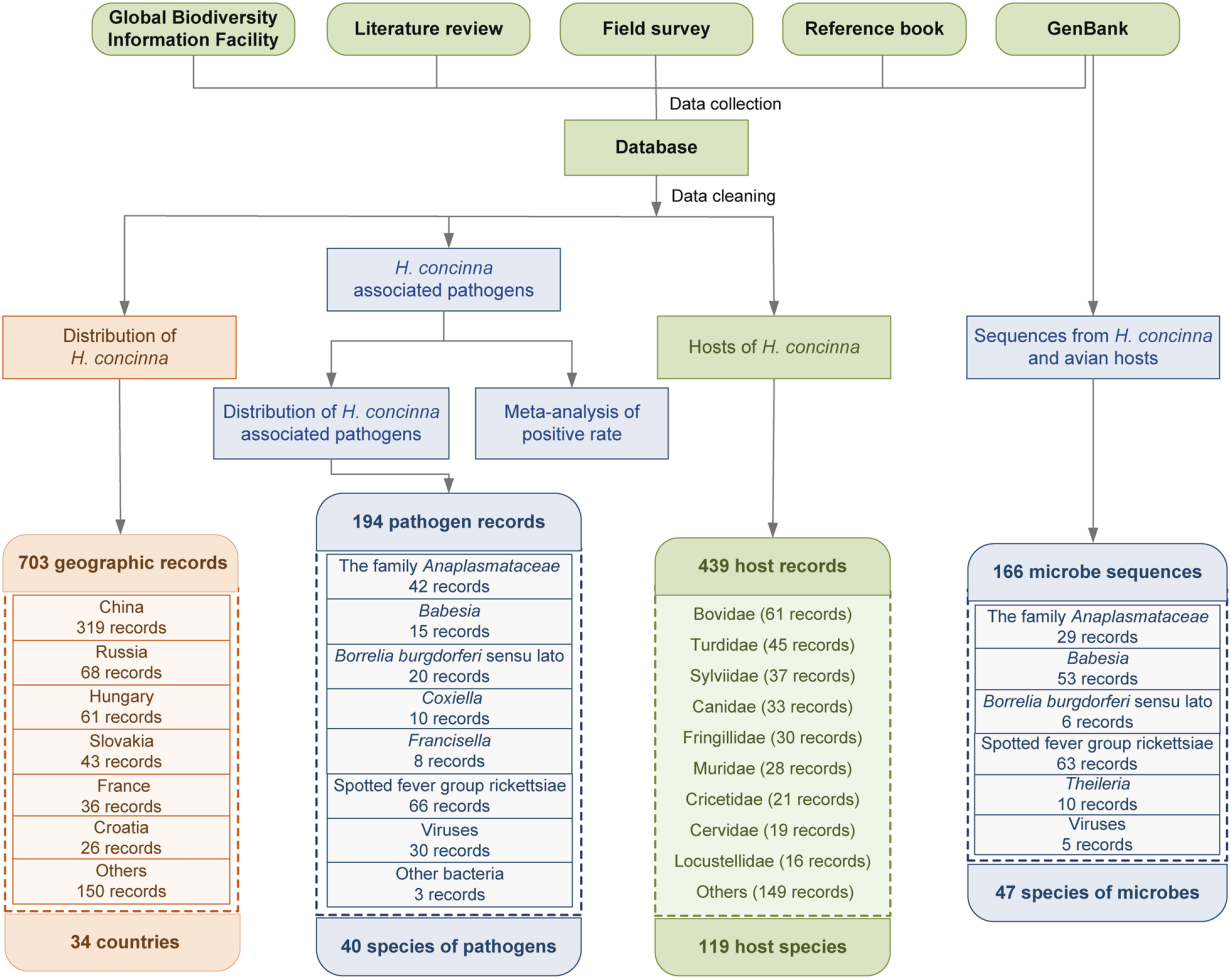
Study design and records. *H. concinna Haemaphysalis concinna*

Analysis of the aggregated data revealed that there were at least 703 geographic records of *H. concinna* in 34 countries, with 40 species of pathogens identified. A total of 439 records for 119 host species were documented. In addition, 166 microbe sequences from *H. concinna* and avian hosts were collected, representing 47 different microbial species (Fig. 1, Additional file 4: Text S2).

### Geographical distribution of *H. concinna*

**Fig. 2:**
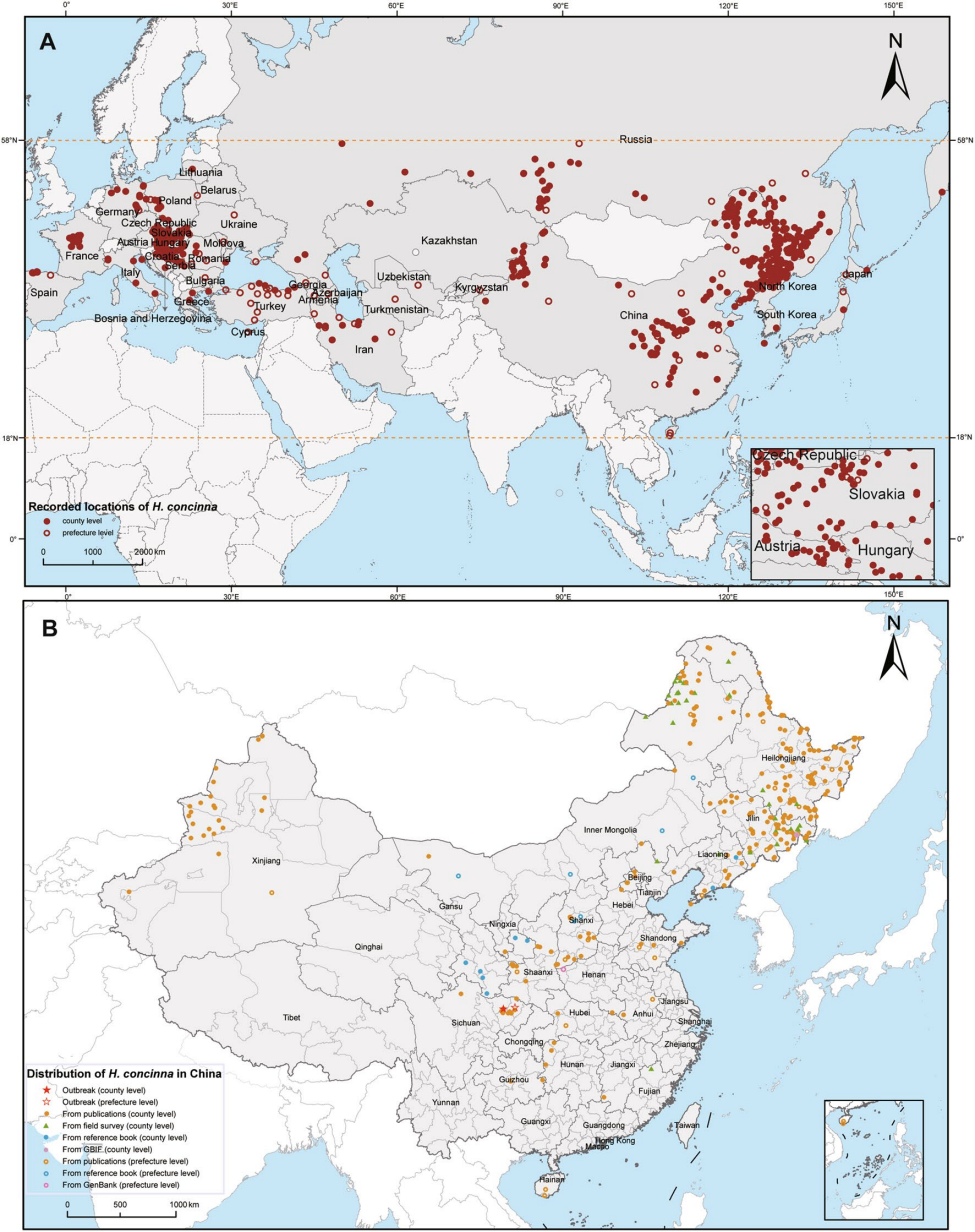
Geographical distribution of *Haemaphysalis concinna.* **A** Recorded locations of *H. concinna* worldwide. Closed circles represent the county-level regions; open circles represent the prefecture-level regions. The tick species was distributed in the regions between 18°–58° latitude in the northern hemisphere. **B** Recorded locations of *H. concinna* in China. Five-pointed stars represent regions with *H. concinna* outbreaks

*Haemaphysalis concinna* was distributed between 18° to 58° in the northern latitudes of the Eurasian continent and could be found in coastal and inland regions (Fig. 2A). In China, *H. concinna* was widely reported in 21 provinces, 76 prefectures, and 146 counties. It was mainly concentrated in the northeast region of China, but both outbreaks of *H. concinna* occurred in the southwest region (Sichuan Province) (Fig. 2B). In Russia, it was predominantly distributed in the southern region, most frequently reported in the Amur Oblast, Khabarovsk territory, Altai, and Kemerovo Oblast. Additionally, *H. concinna* was reported in many European countries, particularly in Central Europe, such as the Czech Republic, Slovakia, Hungary, and Austria.

### Distribution and prevalence of *H. concinna*-associated pathogens

**Fig. 3:**
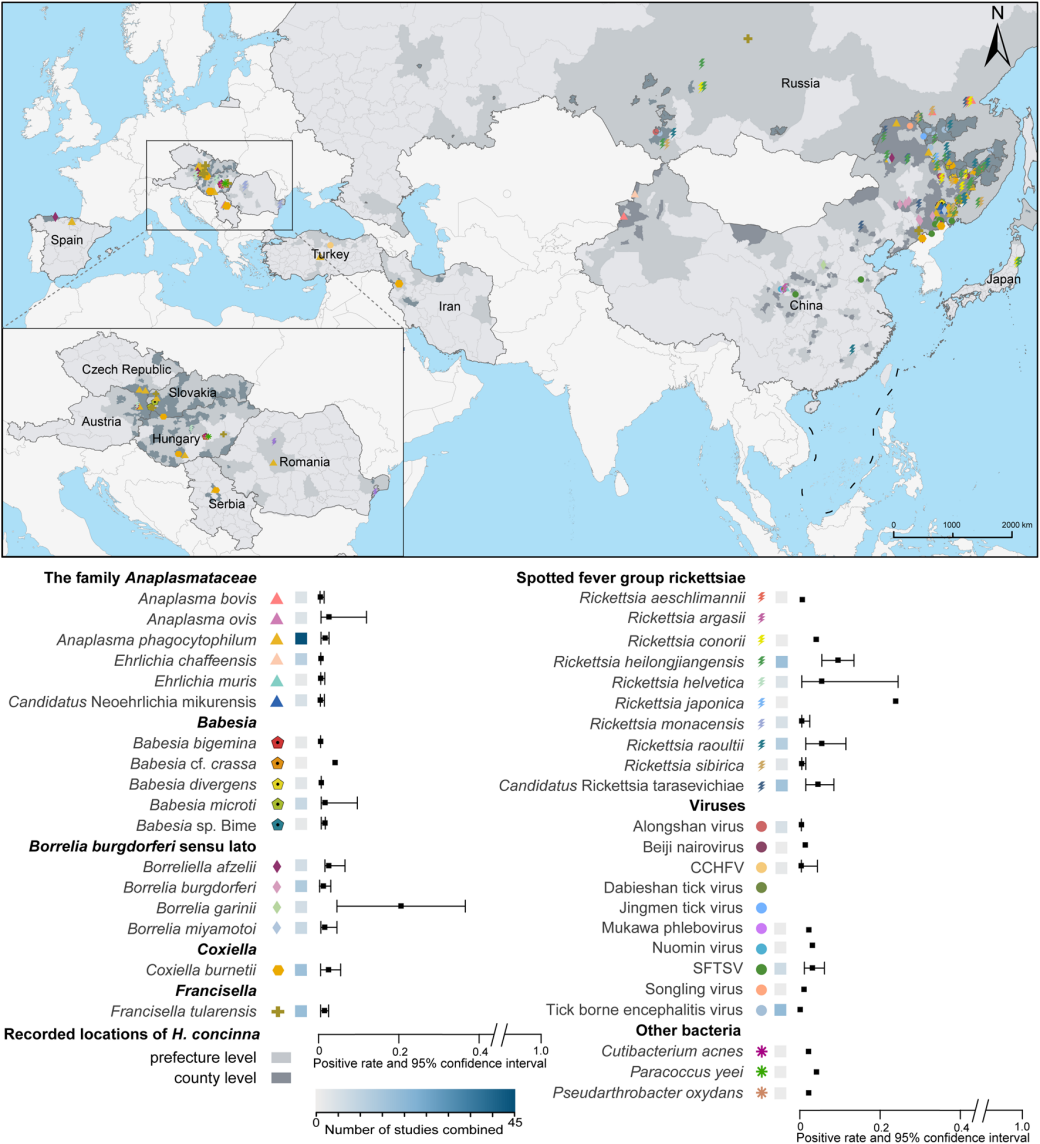
Geographical distribution and prevalence of *Haemaphysalis concinna*-associated pathogens. *CCHFV* Crimean-Congo hemorrhagic fever virus, *SFTSV* severe fever with thrombocytopenia syndrome virus

We plotted the spatial distribution and prevalence of *H. concinna*-associated pathogens (Fig. 3, Additional file 5: Fig. S2). Although *H. concinna* was widely distributed throughout Eurasia, most pathogens were predominantly found in Northeast China. *Anaplasma phagocytophilum* was considered to be the most widely distributed pathogen, reported in ten countries. Spotted fever group rickettsiae (SFGR) were also commonly reported, particularly in northeastern China and southern Russia*.* In China, *H. concinna* harbored the highest variety of pathogens (32 species), including six species of the *Anaplasmataceae* family, four species of *Babesia*, four species of *B. burgdorferi* sensu lato, seven species of SFGR, nine species of viruses, as well as *C. burnetii* and *Francisella tularensis*. Southern Russia also showed significant pathogen diversity of *H. concinna*, involving two species from the *Anaplasmataceae* family, six species of SFGR, as well as Alongshan virus, tick-borne encephalitis virus, and *F. tularensis*. Pathogens were sporadically distributed in the central region of Europe, including two species from the *Anaplasmataceae* family, two species of *Babesia*, two species of SFGR, *Borreliella afzelii*, as well as *C. burnetii*, *F. tularensis*, and other bacteria. Crimean-Congo hemorrhagic fever virus was only reported in Turkey.

Among the 40 *H. concinna*-associated pathogens, most had low positive rates, but certain species of SFGR showed relatively higher positive rates (Fig. 3). *Rickettsia japonica* had the highest positive rate of 0.23, followed by *R. heilongjiangensis* at 0.09 (95% CI 0.05–0.13). Both *R. raoultii* and *Rickettsia helvetica* showed positive rates of 0.05 (95% CI 0.01–0.11 for *R. raoultii* and 95% CI 0.00–0.24 for *R. helvetica*). Additionally, *B. garinii* also had a high positive rate (0.20, 95% CI 0.04–0.46). Other pathogens exhibited low positive rates, none exceeding 0.04. *Anaplasma ovis* had the highest positive rate of 0.02 (95% CI 0.00–0.12) in the family *Anaplasmataceae*. All positive rates of *Babesia* species were < 0.01 except for *Babesia* cf. *crassa* at 0.03. Ten virus species were detected, with severe fever with thrombocytopenia syndrome virus (0.03; 95% CI 0.01–0.06) and Nuomin virus (0.03) having relatively higher prevalence.

### Hosts of *H. concinna*

**Fig. 4:**
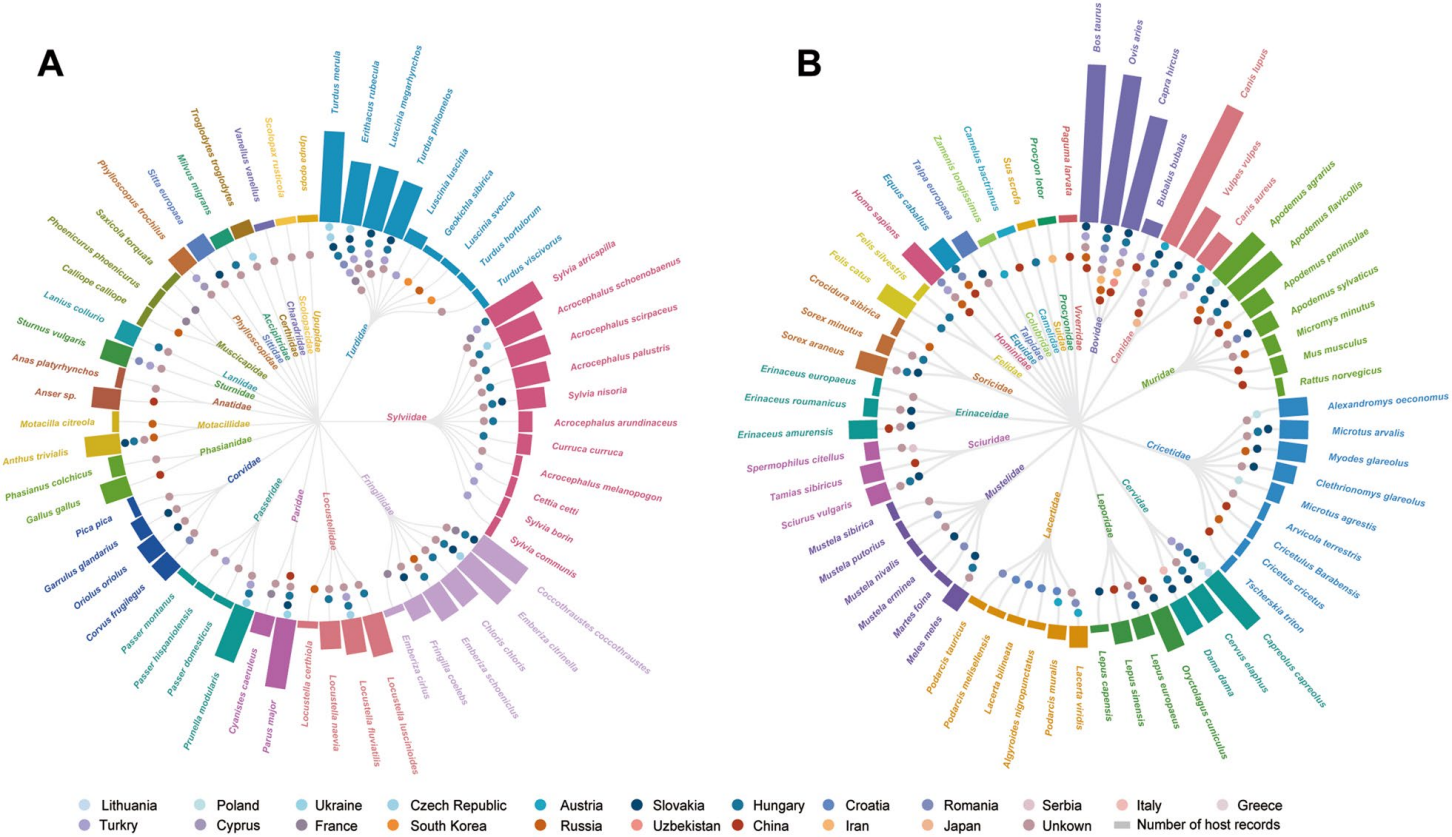
Hosts of *Haemaphysalis concinna* in different countries. **A** Aves parasitized by *H. concinna*. The inner circle displays the names of host families, and the outer circle displays the names of host species. Solid circles indicate the countries where the hosts of *H. concinna* were identified, and solid rectangles represent the number of records for the hosts. **B** Mammalia and Lepidosauria parasitized by *H. concinna*

*Haemaphysalis concinna* had a broad host range, including Aves (birds), Mammalia (mammals), and Lepidosauria (lizards, snakes). A total of 119 species belonging to 40 animal families could serve as hosts for *H. concinna*. Birds, ungulates, carnivores, rodents, and lagomorphs were relatively common among the identified hosts. Notably, there were 58 species of bird hosts for *H. concinna* (Fig. 4A). These birds were frequently observed in Europe, particularly in Hungary, which stood out as a prominent location harboring 23 species of avian hosts. Additionally, *H. concinna* was also reported to feed on 54 species of mammals and seven lepidosaur species (Fig. 4B). Ungulates such as *Bos taurus* (cattle), *Ovis aries* (sheep), and *Capra hircus* (goat) were common hosts. The carnivore species *Canis lupus* (dog) was recognized as the most widely distributed host, with records in eight countries. Furthermore, a diverse group of 19 rodent species and four lagomorph species were known hosts for *H. concinna*.

### Role of birds in transmitting *H. concinna*-associated microbes

**Fig. 5:**
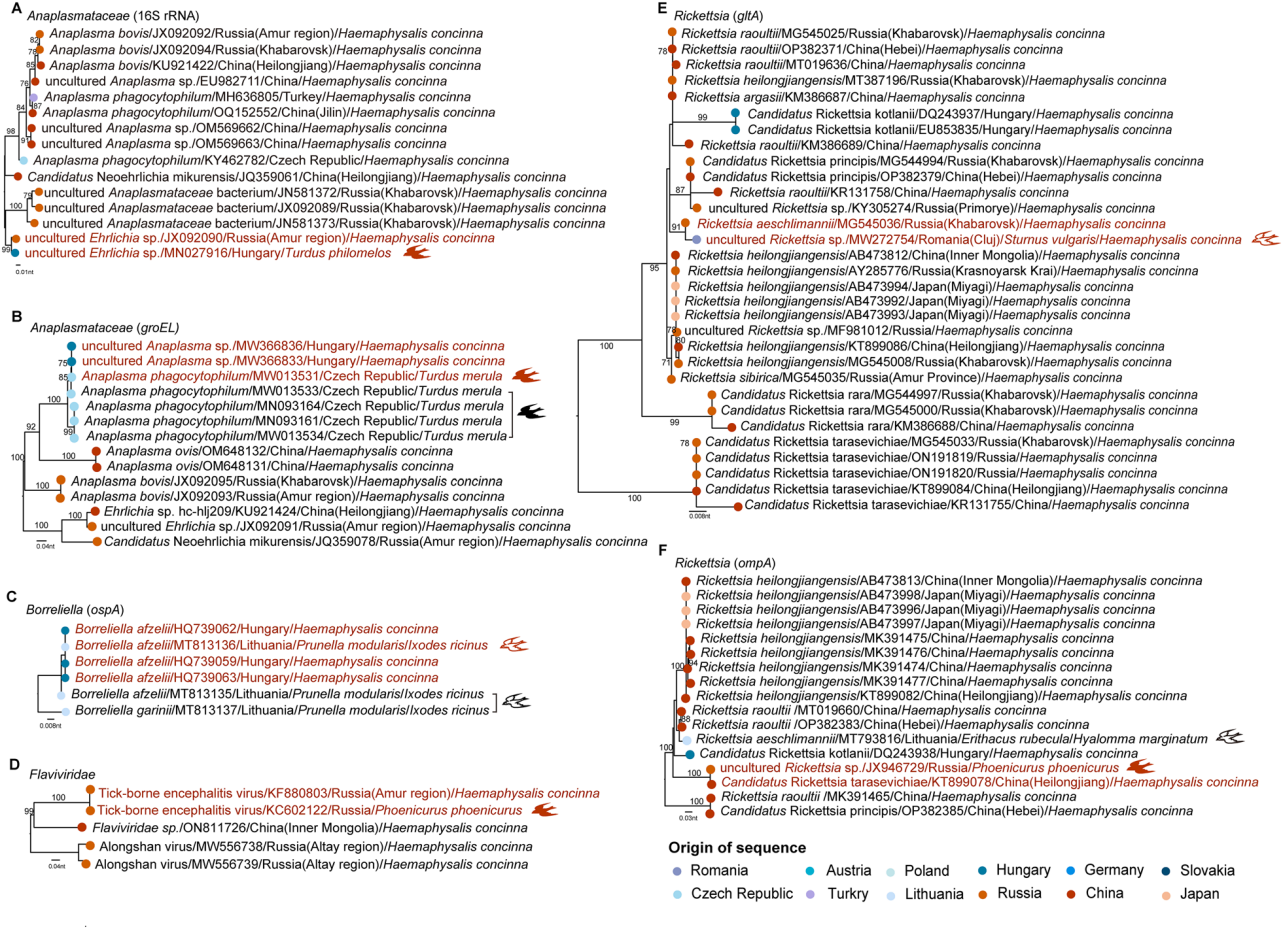
Phylogenetic analysis of *Haemaphysalis concinna*-associated microbes and avians. Solid bird shape represents sequences derived from birds, while hollow bird shape represents sequences derived from ticks parasitized on birds. Sequences that share a close genetic relationship between ticks and birds are highlighted in red

Birds could play a crucial role in transmitting *H. concinna*-associated microbes because of genetic similarities observed between the *H. concinna*-associated microbes and avian hosts across different geographical regions (Fig. 5, Additional file 6: Fig. S3). For instance, uncultured *Ehrlichia* sp. identified from *H. concinna* in the Russian Far East and *Turdus philomelos* in Hungary showed a high similarity with 98.1% nucleotide identities of 16S rRNA gene (Fig. 5A). *Turdus philomelos* was a commonly parasitized bird species by *H. concinna*. Similar genetic relationships were observed in other microbes (5B, 5D, and 5F). *Rickettsia aeschlimannii* and uncultured *Rickettsia* sp., two *Rickettsia* strains isolated from *H. concinna* at distant locations, exhibited 100% similarity, one of which parasitized on *Sturnus vulgaris* (Fig. 5E). Similar genetic relationships were also observed in other tick species (Fig. 5C). Geographical analysis further revealed that the distribution of *H. concinna* was highly consistent with the migration routes of birds in China and Europe (Additional file 7: Figs. S4, S5), supporting the hypothesis that migratory birds might play a key role in dispersal of ticks and tick-borne microbes.

### Predicted distribution of* H. concinna*

Suitable areas for *H. concinna* were predicted using ecological niche modeling. The parameters of the optimal model were: the regularization multiplier was 2.9 and the feature combination was LPTH (linear, product, threshold, hinge). Nine independent variables, including BIO11, BIO12, BIO18, land cover, BIO4, elevation, BIO15, BIO19, and BIO17, were selected to develop the prediction model. The Maxent model with 25 replicates yielded an average AUC value of 0.966 (SD 0.016), indicating the high predictive performance of our model. The main environmental variables influencing the geographical distribution of *H. concinna* were BIO11, BIO12, BIO18, and land cover. The areas with a mean temperature of coldest quarter of 0.4 ℃, annual precipitation of 641.5 mm, precipitation of warmest quarter of 407.6 mm, and land cover of urban construction land, broadleaf deciduous forest, or cropland were predicted to have the highest probability of suitability for *H. concinna* (Additional file 8: Figs. S6–S8, Table S3).

**Fig. 6:**
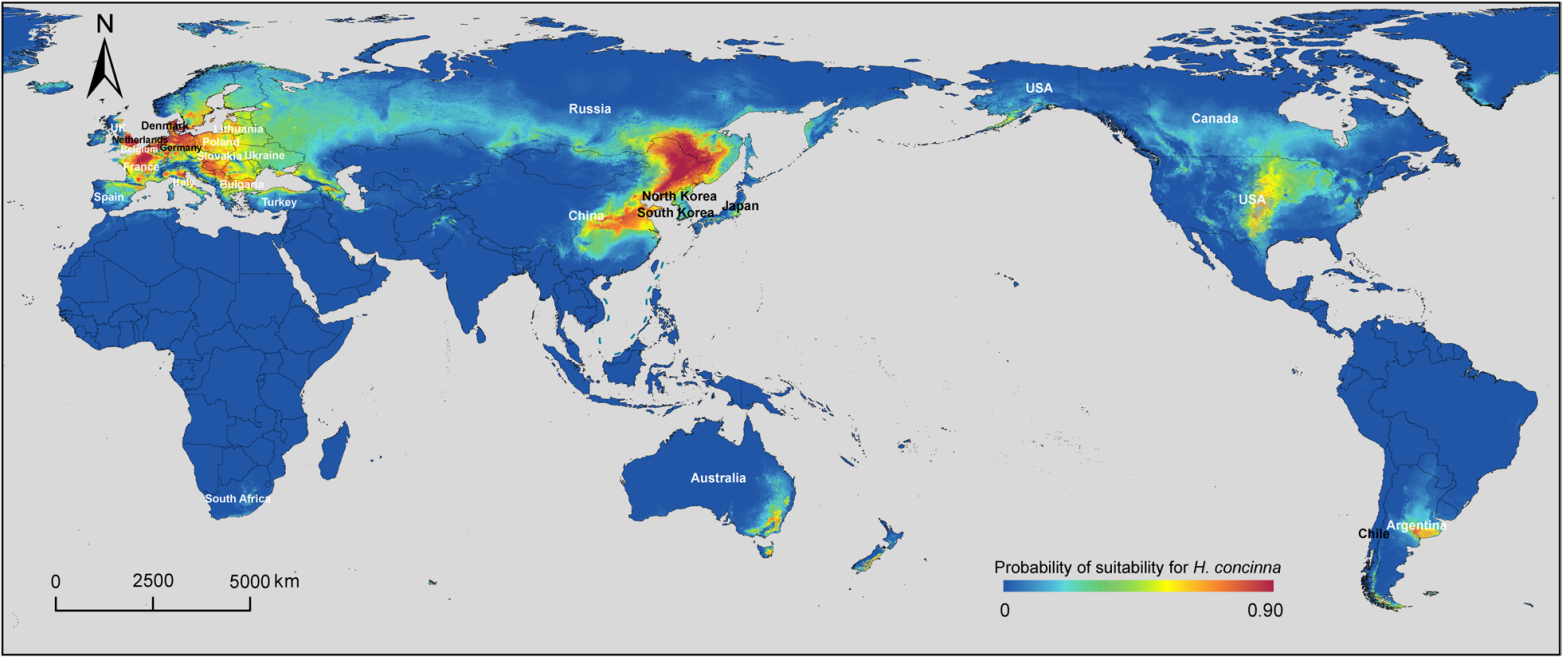
Map of the potential global distribution of *Haemaphysalis concinna*. The ecological niche modeling displays the predicted probability of *H. concinna* occurrence

According to the prediction model, the Eurasian continent remained the most suitable region for the survival of *H. concinna* (Fig. 6). In Asia, the most favorable regions for the tick species were Northeast China, North China, East China, Central China, North Korea, Turkey, Japan, and South Korea. In Europe, the most suitable habitats for *H. concinna* were located in Central and Western Europe, such as France, Belgium, The Netherlands, Hungary, Slovakia, Poland, and Germany. In addition, the southern region of Russia was also projected to be suitable for the tick species. Surprisingly, *H. concinna* had the potential to survive in other continents where the tick had never been recorded before, including southern Canada and central US. The prediction model also indicated that *H. concinna* might inhabit countries in the southern hemisphere, such as Argentina, Australia, Chile, and South Africa.

## Discussion

Our analysis of the integrated database suggested that *H. concinna* exhibits a wide geographical distribution, broad host spectrum, and high level of vector competence. The predictive model indicated that *H. concinna* has the potential to inhabit larger regions, which requires sustained vigilance and public health response.

Due to its high adaptability to the environment, *H. concinna* can be found in widely varying habitats across the Eurasian continent, causing extensive negative impacts on human and animal health [2]. In China, *H. concinna* is more prevalent in the northeastern region. However, both outbreaks of the tick species occurred in Sichuan Province in 1999 and 2000 [3, 4], where the tick reports were relatively limited. The possible reasons are as follows. Sichuan Province has a favorable climate and abundant resources, providing a suitable environment and enough forage for rabbit reproduction [18]. Rabbits can serve as an ideal host for *H. concinna*, as the tick species can complete its entire life within rabbits in a shorter time [19]. In addition, policies aimed at protecting hares were issued by the authorities in 1988 and 2000 [20], which may have substantially increased the population of hares and contributed to the subsequent outbreaks of *H. concinna*.

Our findings revealed that *H. concinna* may harbor at least 83 microbes (Additional file 9: Figs. S9, S10), with 40 being pathogenic to humans. This is a higher number than for *H. longicornis*, which carries 59 microbes [21], and *Ixodes persulcatus*, which carries 51 microbes [22]. Notably, *H. concinna* may carry a range of emerging viruses, including Alongshan virus [23], Beiji nairovirus, Dabieshan tick virus, Jingmen tick virus, severe fever with thrombocytopenia syndrome virus, and Songling virus, which has attracted public health attention globally. SFGR carried by *H. concinna* increases the risk of rickettsioses, with *R. japonica* being the most prevalent species and leading to fulminant purpura in humans [24]. *Rickettsia heilongjiangensis* is also commonly identified in *H. concinna*, contributing to the transmission of Far Eastern tick-borne spotted fever [25]. Additionally, six species in the family *Anaplasmataceae* carried by *H. concinna* can cause human anaplasmosis and ehrlichiosis [26]. Various *B. burgdorferi* s.l. species, such as *B. garinii*, have been detected in *H. concinna* and are responsible for Lyme disease in Eurasia [27]. *Haemaphysalis concinna* also carries a diversity of bacteria capable of infecting humans, such as *C. buenetii* and *F. tularensis*. Notably, some mentioned pathogens are biologically transmitted by *H. concinna*, while others may be accidental infections acquired from their hosts. Therefore, the role of *H. concinna* as a vector needs to be further studied.

*Haemaphysalis concinna* fed on 119 species of host animals, which is more than *H. longicornis* (77 host species) and *I. persulcatus* (46 host species) [21, 22]. Notably, birds can serve as hosts for *H. concinna* [7]. Previous studies primarily focused on the phylogenetic relationships among ticks or tick-borne microbes [9, 10, 28]. Our research directly involved avian carriers of microbes, indicating their intermediary role in the transmission of tick-borne microbes. Many of these birds migrate long distances, even crossing oceans, providing opportunities for the spread of *H. concinna* [8]. Additionally, birds can act as carriers of pathogens and potentially transfer them between ticks during co-feeding [29]. Therefore, the role of birds in spreading ticks and tick-borne pathogens should not be underestimated.

Recent observations have indicated the expansion of *H. concinna* in Europe, with documented occurrences in southwest Poland and Lithuania [5, 6]. Our predictive model also confirmed that the distribution of *H. concinna* is not confined to its current range and could potentially expand in the future. Several key variables, including BIO11, BIO12, BIO18, and land cover, contributed to 83.8% of the explanatory power in our model. Temperature and precipitation are identified as crucial factors that shape *H. concinna*'s life cycle and influence its distribution, which aligns with the findings of Hubalek's study [30]. The tick species generally survive in areas with moderate precipitation levels and temperature [31]. Additionally, *H. concinna* demonstrates adaptability to various land types. Previous studies have shown that the highly suitable habitats for *H. concinna* in China are primarily located in Northeast China and Northwest China, as determined by Boosted Regression Tree models [1]. However, our study predicted a wider distribution range, including North, East, and Central China. These disparities could be attributed to variances in data collection and modeling methods in different studies. Although there is limited research on predicting suitable habitats for *H. concinna* with inconsistent results, the existing studies indicate that *H. concinna* poses a potential risk of expansion and may impact larger regions.

The limitations of this study should be acknowledged. First, the inclusion of only Chinese and English literature may have resulted in a loss of relevant information from publications in other languages. Second, due to the lack of pathogen detection or low detection sensitivity in some areas, areas with no pathogen records currently may still have pathogens, which may lead to an underestimation of the distribution range and prevalence of *H. concinna*-associated pathogens. Third, the exploration of the role of birds in transmitting *H. concinna*-associated microbes is only dependent on conserved genes in this study, which may not fully represent the phylogeny of the entire genome. Future research with the exploration of the whole genome will be needed to gather more comprehensive evidence and confirm the role.

## Conclusions

*Haemaphysalis concinna* and associated pathogens pose a growing challenge to global public health as this tick species can inhabit a variety of countries and harbor various pathogens, negatively affecting multiple host animals, including humans. Avian hosts can facilitate the spread of pathogens over long distances, amplifying their adverse impacts. Furthermore, our predictive model indicated that *H. concinna* may affect even more geographical regions worldwide. To fight against *H. concinna* and associated diseases, high-risk countries should collaborate on monitoring and controlling ticks, identifying pathogens, and developing diagnostic tools. Further studies are warranted to better understand the mechanisms of *H. concinna* and avian hosts in transmitting these pathogens.

### Supplementary Information

**Additional file 1: Figure S1.** PRISMA flow diagram of study selection process. **Text S1. **References for *Haemaphysalis concinna*.

**Additional file 2: Table S1. **Environmental and meteorological variables downloaded for ecological modeling for *Haemaphysalis concinna.*

**Additional file 3: Table S2. **Known occurrence locations of *Haemaphysalis concinna *used for ecological niche modelling.

**Additional file 4: Text S2.** Basic characteristics of data collection.

**Additional file 5: Figure S2. **The distribution of pathogens in different countries.

**Additional file 6: Figure S3. **Phylogenetic analysis of *Haemaphysalis concinna*-associated microbes

**Additional file 7: Figure S4. **Migration routes of birds in Europe susceptible to parasitism by *Haemaphysalis concinna*. **Figure S5. **Migratory birds in China primarily exhibit three migration routes.

**Additional file 8: Figure S6.** The results of Maxent model for *Haemaphysalis concinna.* **Figure S7.** Jackknife plots of Maxent model for *Haemaphysalis concinna *prediction. **Table S3.** Relative contributions of the environmental and meteorological variables to the Maxent model. ** Figure S8. **Response curves of environmental variables to probability of *Haemaphysalis concinna *presence

**Additional file 9: Figure S9. **Prevalence of *Haemaphysalis concinna*-associated microbes. **Figure S10. **Meta-analysis of the prevalence of each *Haemaphysalis concinna*-associated microbes.

## Data Availability

The data supporting the findings of the study must be available within the article and/or its supplementary materials, or deposited in a publicly available database.
