# Supplementary material for: An integrated data analysis reveals distribution, hosts, and pathogen diversity of Haemaphysalis concinna
Source: Parasit Vectors. 2024 Feb 27;17:92. doi: 10.1186/s13071-024-06152-5 (PMC10900579; doi:10.1186/s13071-024-06152-5)
Supplement: Supplementary file 1 — Additional file 1: Figure S1. PRISMA flow diagram of study selection process. Text S1. References for Haemaphysalis concinna. [file 13071_2024_6152_MOESM1_ESM.pdf]

Figure S1: PRISMA flow diagram of study selection process.

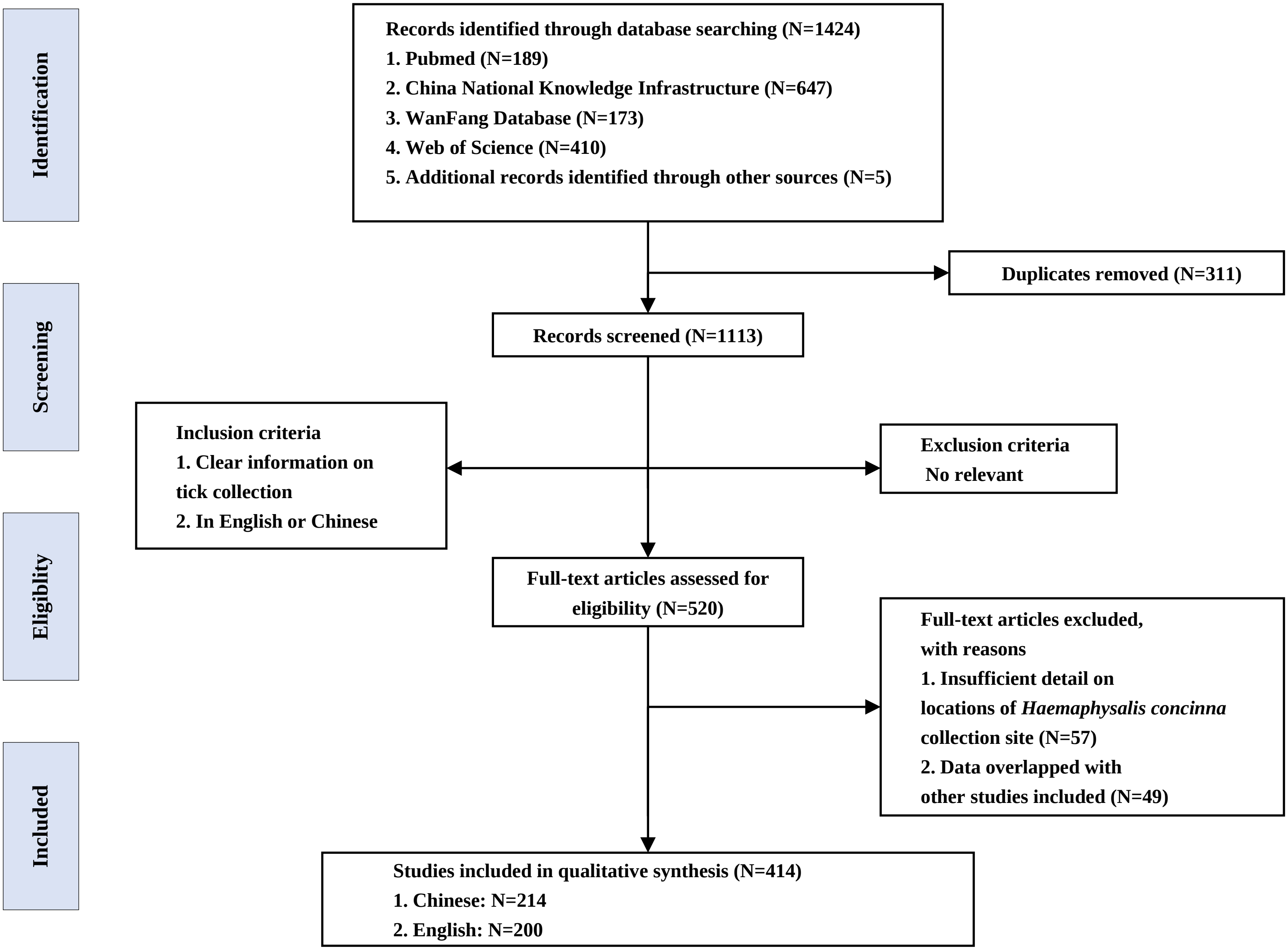

Text S1: References for *Haemaphysalis concinna*

We extract the following information from the publications below: first author, publication year, collection locations, number of *H. concinna* sample tested, number of samples positive for a specific pathogen, host animals.

1. Rudolf I, Mendel J, Sikutová S, et al. 16S rRNA gene-based identification of cultured bacterial flora from host-seeking Ixodes ricinus, Dermacentor reticulatus and *Haemaphysalis concinna* ticks, vectors of vertebrate pathogens. *Folia Microbiol (Praha)* 2009; **54**(5): 419-28.
2. Meng H, Xu S, Yu Z, et al. Abundance and seasonal activity of *Haemaphysalis concinna* (Acari: Ixodidae) at the border between China and Russia in Northern Inner Mongolia, China. *Parasit Vectors* 2016; **9**: 1.
3. Iori A, Di Paolo M. Acarological studies in two protected areas of Central Italy. *Parassitologia* 1999; **41 Suppl 1**: 53-55.
4. Hornok S. Allochronic seasonal peak activities of Dermacentor and Haemaphysalis spp. under continental climate in Hungary. *Vet Parasitol* 2009; **163**(4): 366-69.
5. Tomanović S, Chochlakis D, Radulović Z, et al. Analysis of pathogen co-occurrence in host-seeking adult hard ticks from Serbia. *Exp Appl Acarol* 2013; **59**(3): 367-76.
6. Marcutan I-D, Sandor AD, Kalmar Z, et al. Anaplasma phagocytophilum in ticks and tissues collected from wild birds in Romania. *Scientia Parasitologica* 2015; **16**(3): 103-11.
7. Jiang JF, Jiang BG, Yu JH, et al. Anaplasma phagocytophilum infection in ticks, China-Russia border. *Emerg Infect Dis* 2011; **17**(5): 932-34.
8. Svitálková Z, Haruštiaková D, Mahríková L, et al. Anaplasma phagocytophilum prevalence in ticks and rodents in an urban and natural habitat in South-Western Slovakia. *Parasit Vectors* 2015; **8**: 276.
9. Pazhoom F, Ebrahimzade E, Shayan P, Nabian S. Anaplasma spp. identification in hard ticks of Iran: First report of Anaplasma bovis in Haemaphysalis inermis. *Acarologia* 2016; **56**(4): 497-504.
10. Flaisz B, Sulyok KM, Kováts D, et al. Babesia genotypes in *Haemaphysalis concinna* collected from birds in Hungary reflect phylogeographic connections with Siberia and the Far East. *Ticks Tick Borne Dis* 2017; **8**(4): 666-70.
11. Blaschitz M, Narodoslavsky-Gföller M, Kanzler M, Stanek G, Walochnik J. Babesia species occurring in Austrian Ixodes ricinus ticks. *Appl Environ Microbiol* 2008; **74**(15): 4841-46.
12. Hamšíková Z, Kazimírová M, Haruštiaková D, et al. Babesia spp. in ticks and wildlife in different habitat types of Slovakia. *Parasit Vectors* 2016; **9**(1): 292.
13. Andersson MO, Tolf C, Tamba P, et al. Babesia, Theileria, and Hepatozoon species in ticks infesting animal hosts in Romania. *Parasitol Res* 2017; **116**(8): 2291-97.
14. Arzamani K, Saghafipour A, Hashemi SA, et al. Biodiversity Indices and Medically Importance of Ticks in North Khorasan Province, Northeast of Iran. *J Arthropod Borne Dis* 2021; **15**(2): 187-95.
15. Hoogstraal H. Biological observations on certain Turkish Haemaphysalis ticks (Ixodoidea, Ixodidae). *J Parasitol* 1959; **45**(2): 227-32.
16. Hornok S, Flaisz B, Takács N, et al. Bird ticks in Hungary reflect western, southern, eastern flyway connections and two genetic lineages of Ixodes frontalis and *Haemaphysalis concinna*. *Parasit Vectors* 2016; **9**: 101.
17. Hornok S, Kováts D, Csörgő T, et al. Birds as potential reservoirs of tick-borne pathogens: first evidence of bacteraemia with Rickettsia helvetica. *Parasit Vectors* 2014; **7**: 128.
18. Nebogatkin IV. BIRDS AS THE FEEDERS OF TICKS (ACARI, IXODIDA) IN MEGALOPOLIS OF KYIV. *Vestnik Zoologii* 2014; **48**(5): 467-70.

19. Blaschitz M, Narodoslavsky-Gfoeller M, Kanzler M, Walochnik J, Stanek G. Borrelia burgdorferi sensu lato genospecies in questing Ixodes ricinus ticks in Austria. *International Journal of Medical Microbiology* 2008; **298**: 168-76.
20. Espí A, Del Cerro A, Somoano A, et al. Borrelia burgdorferi sensu lato prevalence and diversity in ticks and small mammals in a Lyme borreliosis endemic Nature Reserve in North-Western Spain. Incidence in surrounding human populations. *Enferm Infecc Microbiol Clin* 2017; **35**(9): 563-8.
21. Jiang BG, Jia N, Jiang JF, et al. Borrelia miyamotoi Infections in Humans and Ticks, Northeastern China. *Emerg Infect Dis* 2018; **24**(2): 236-41.
22. Hubálek Z, Anderson JF, Halouzka J, Hájek V. Borreliae in immature Ixodes ricinus (Acari:Ixodidae) ticks parasitizing birds in the Czech Republic. *J Med Entomol* 1996; **33**(5): 766-71.
23. Szekeres S, Claudia Coipan E, Rigó K, et al. Candidatus Neoehrlichia mikurensis and Anaplasma phagocytophilum in natural rodent and tick communities in Southern Hungary. *Ticks Tick Borne Dis* 2015; **6**(2): 111-16.
24. Liu H, Li Q, Zhang X, et al. Characterization of rickettsiae in ticks in northeastern China. *Parasit Vectors* 2016; **9**(1): 498.
25. Hornok S, Kováts D, Horváth G, Kontschán J, Farkas R. Checklist of the hard tick (Acari: Ixodidae) fauna of Hungary with emphasis on host-associations and the emergence of Rhipicephalus sanguineus. *Exp Appl Acarol* 2020; **80**(3): 311-28.
26. Tekin S, Bursali A, Mutluay N, Keskin A, Dundar E. Crimean-Congo hemorrhagic fever virus in various ixodid tick species from a highly endemic area. *Vet Parasitol* 2012; **186**(3-4): 546-52.
27. Egyed L, Makrai L. Cultivable internal bacterial flora of ticks isolated in Hungary. *Exp Appl Acarol* 2014; **63**(1): 107-22.
28. Galuppi R, Aureli S, Bonoli C, Caffara M, Tampieri MP. Detection and molecular characterization of Theileria sp. in fallow deer (Dama dama) and ticks from an Italian natural preserve. *Res Vet Sci* 2011; **91**(1): 110-15.
29. Sréter-Lancz Z, Széll Z, Sréter T, Márialigeti K. Detection of a novel Francisella in Dermacentor reticulatus: a need for careful evaluation of PCR-based identification of Francisella tularensis in Eurasian ticks. *Vector Borne Zoonotic Dis* 2009; **9**(1): 123-26.
30. Shpynov S, Fournier PE, Rudakov N, Tankibaev M, Tarasevich I, Raoult D. Detection of a rickettsia closely related to Rickettsia aeschlimannii, "Rickettsia heilongjiangensis," Rickettsia sp. strain RpA4, and Ehrlichia muris in ticks collected in Russia and Kazakhstan. *J Clin Microbiol* 2004; **42**(5): 2221-23.
31. Rigó K, Gyuranecz M, Tóth AG, Földvári G. Detection of Borrelia burgdorferi Sensu Lato and Anaplasma phagocytophilum in small mammals and ectoparasites in Hungary. *Vector Borne Zoonotic Dis* 2011; **11**(11): 1499-501.
32. Spitalská E, Kocianová E. Detection of Coxiella burnetii in ticks collected in Slovakia and Hungary. *Eur J Epidemiol* 2003; **18**(3): 263-66.
33. Higgins JA, Hubalek Z, Halouzka J, et al. Detection of Francisella tularensis in infected mammals and vectors using a probe-based polymerase chain reaction. *Am J Trop Med Hyg* 2000; **62**(2): 310-18.
34. Kovalevskiy AV, Oplachko SS, Zubko KS, et al. Distribution and ecology of *Haemaphysalis concinna* (Parasitiformes, Ixodidae) in the Kuznetsk-Salair mountain area (Kemerovo region and adjacent regions, Russia). *Invertebrate Zoology* 2020; **17**(2): 133-44.
35. Shao JW, Zhang XL, Li WJ, Huang HL, Yan J. Distribution and molecular characterization of rickettsiae in ticks in Harbin area of Northeastern China. *PLoS Negl Trop Dis* 2020; **14**(6): e0008342.
36. García-Sanmartín J, Barandika JF, Juste RA, García-Pérez AL, Hurtado A. Distribution and molecular detection of Theileria and Babesia in questing ticks from northern Spain. *Med Vet Entomol* 2008; **22**(4): 318-25.

37. Lebedeva NN, Korenberg EI. Distribution of *Haemaphysalis concinna* Koch in the Soviet Union and some general features of its ecology. *Folia Parasitol (Praha)* 1981; **28**(3): 249-61.
38. Kazimírová M, Hamšíková Z, Špitalská E, et al. Diverse tick-borne microorganisms identified in free-living ungulates in Slovakia. *Parasit Vectors* 2018; **11**(1): 495.
39. Khasnatinov MA, Liapunov AV, Manzarova EL, Kulakova NV, Petrova IV, Danchinova GA. The diversity and prevalence of hard ticks attacking human hosts in Eastern Siberia (Russian Federation) with first description of invasion of non-endemic tick species. *Parasitol Res* 2016; **115**(2): 501-10.
40. Hornok S, Takács N, Kontschán J, et al. Diversity of Haemaphysalis-associated piroplasms of ruminants in Central-Eastern Europe, Hungary. *Parasit Vectors* 2015; **8**: 627.
41. Qin T, Shi M, Zhang M, Liu Z, Feng H, Sun Y. Diversity of RNA viruses of three dominant tick species in North China. *Front Vet Sci* 2022; **9**: 1057977.
42. Cheng C, Fu W, Ju W, et al. Diversity of spotted fever group Rickettsia infection in hard ticks from Suifenhe, Chinese-Russian border. *Ticks Tick Borne Dis* 2016; **7**(5): 715-19.
43. Hornok S, Szekeres S, Horváth G, et al. Diversity of tick species and associated pathogens on peri-urban wild boars - First report of the zoonotic Babesia cf. crassa from Hungary. *Ticks Tick Borne Dis* 2022; **13**(3): 101936.
44. Rataud A, Galon C, Bournez L, Henry PY, Marsot M, Moutailler S. Diversity of Tick-Borne Pathogens in Tick Larvae Feeding on Breeding Birds in France. *Pathogens* 2022; **11**(8).
45. Krčmar S. Diversity, ecology, and seasonality of hard ticks (Acari: Ixodidae) in eastern Croatia. *J Vector Ecol* 2019; **44**(1): 18-29.
46. Krčmar S, Klobučar A, Vucelja M, et al. DNA barcoding of hard ticks (Ixodidae), notes on distribution of vector species and new faunal record for Croatia. *Ticks Tick Borne Dis* 2022; **13**(3): 101920.
47. Szekeres S, Coipan EC, Rigó K, et al. Eco-epidemiology of Borrelia miyamotoi and Lyme borreliosis spirochetes in a popular hunting and recreational forest area in Hungary. *Parasit Vectors* 2015; **8**: 309.
48. Nosek J, Lichard M, Sztankay M. The ecology of ticks in the Tribec and Hronský Inovec Mountains. *Bull World Health Organ* 1967; **36 Suppl**(Suppl 1): 49-59.
49. Nosek J. The ecology, bionomics and behaviour of *Haemaphysalis (Haemaphysalis) concinna* tick. *Z Parasitenkd* 1971; **36**(3): 233-41.
50. Acici M, Bolukbas CS, Beyhan YE, Pekmezci GZ, Gurler AT, Umur S. Ectoparasites on roe deer (Capreolus capreolus) in Samsun, Turkey. *Turkish Journal of Veterinary & Animal Sciences* 2012; **36**(4): 456-9.
51. Vacek Z, Cukor J, Vacek S, et al. Effect of forest structures and tree species composition on common tick (Ixodes ricinus) abundance-Case study from Czechia. *Forest Ecology and Management* 2023; **529**.
52. Rahravani M, Moravedji M, Mostafavi E, et al. The epidemiological survey of Coxiella burnetii in small ruminants and their ticks in western Iran. *BMC Vet Res* 2022; **18**(1): 292.
53. Liu Z, Li L, Xu W, et al. Extensive diversity of RNA viruses in ticks revealed by metagenomics in northeastern China. *PLoS Negl Trop Dis* 2022; **16**(12): e0011017.
54. Rehácek J, Urvölgyi J, Kocianová E, Sekeyová Z, Vavreková M, Kovácová E. Extensive examination of different tick species for infestation with Coxiella burnetii in Slovakia. *Eur J Epidemiol* 1991; **7**(3): 299-303.
55. Mediannikov O, Sidelnikov Y, Ivanov L, Fournier PE, Tarasevich I, Raoult D. Far eastern tick-borne rickettsiosis: identification of two new cases and tick vector. *Ann N Y Acad Sci* 2006; **1078**: 80-88.
56. Krcmar S, Veres M, Trilar T. FAUNA OF HARD TICKS (ACARI: IXODIDAE) IN DIFFERENT HABITATS IN CROATIAN PART OF BARANJA. *Sumarski List* 2014; **138**(5-6): 309-14.
57. Slovak M. Finding of the endoparasitoid Ixodiphagus hookeri (Hymenoptera, Encyrtidae) in *Haemaphysalis concinna* ticks in Slovakia. *Biologia* 2003; **58**(5): 890.
58. Wang YN, Jiang RR, Ding H, et al. First Detection of Mukawa Virus in Ixodes persulcatus and *Haemaphysalis concinna* in China. *Front Microbiol* 2022; **13**: 791563.

59. Meng K, Sun W, Cheng Z, Guo H, Liu J, Chai T. First detection of severe fever with thrombocytopenia syndrome virus in the tick species *Haemaphysalis concinna* in Shandong Province, China. *Parasitol Res* 2015; **114**(12): 4703-07.
60. Radulović Z, Chochlakis D, Tomanović S, Milutinović M, Tselentis Y, Psaroulaki A. First detection of spotted fever group Rickettsiae in ticks in Serbia. *Vector Borne Zoonotic Dis* 2011; **11**(2): 111-15.
61. Rubel F, Brugger K, Monazahian M, et al. The first German map of georeferenced ixodid tick locations. *Parasit Vectors* 2014; **7**: 477.
62. Gurycová D. First isolation of Francisella tularensis subsp. tularensis in Europe. *Eur J Epidemiol* 1998; **14**(8): 797-802.
63. Sharifdini M, Norouzi B, Azari-Hamidian S, Karamzadeh N. The first record of ectoparasites of raccoons (Procyon lotor) (Carnivora, Procyonidae) in Iran. *Persian Journal of Acarology* 2021; **10**(1): 41-54.
64. Paulauskas A, Sakalauskas P, Kaminskienė E, Šimkevičius K, Kibiša A, Radzijevskaja J. First record of *Haemaphysalis concinna* (Acari: Ixodidae) in Lithuania. *Ticks Tick Borne Dis* 2020; **11**(5): 101460.
65. Kiewra D, Czułowska A, Dyczko D, Zieliński R, Plewa-Tutaj K. First record of *Haemaphysalis concinna* (Acari: Ixodidae) in Lower Silesia, SW Poland. *Exp Appl Acarol* 2019; **77**(3): 449-54.
66. Shemshad M, Shemshad K, Sedaghat MM, et al. First survey of hard ticks (Acari: Ixodidae) on cattle, sheep and goats in Boeen Zahra and Takistan counties, Iran. *Asian Pac J Trop Biomed* 2012; **2**(6): 489-92.
67. Briciu VT, Titilincu A, Tăţulescu DF, Cârstina D, Lefkaditis M, Mihalca AD. First survey on hard ticks (Ixodidae) collected from humans in Romania: possible risks for tick-borne diseases. *Exp Appl Acarol* 2011; **54**(2): 199-204.
68. Víchová B, Bona M, Miterpáková M, et al. Fleas and Ticks of Red Foxes as Vectors of Canine Bacterial and Parasitic Pathogens, in Slovakia, Central Europe. *Vector Borne Zoonotic Dis* 2018; **18**(11): 611-19.
69. Hubálek Z, Juricová Z, Halouzka J. Francisella tularensis from ixodid ticks in Czechoslovakia. *Folia Parasitol (Praha)* 1990; **37**(3): 255-60.
70. Hubálek Z, Treml F, Halouzka J, Juricová Z, Hunady M, Janík V. Frequent isolation of Francisella tularensis from Dermacentor reticulatus ticks in an enzootic focus of tularaemia. *Med Vet Entomol* 1996; **10**(3): 241-46.
71. Xia LY, Jiang BG, Yuan TT, et al. Genetic Diversity and Coexistence of Babesia in Ticks (Acari: Ixodidae) from Northeastern China. *Vector Borne Zoonotic Dis* 2020; **20**(11): 817-24.
72. Rar VA, Livanova NN, Panov VV, et al. Genetic diversity of Anaplasma and Ehrlichia in the Asian part of Russia. *Ticks Tick Borne Dis* 2010; **1**(1): 57-65.
73. Rar VA, Epikhina TI, Suntsova OV, et al. Genetic variability of Babesia parasites in Haemaphysalis spp. and Ixodes persulcatus ticks in the Baikal region and Far East of Russia. *Infect Genet Evol* 2014; **28**: 270-75.
74. Igolkina Y, Rar V, Vysochina N, et al. Genetic variability of Rickettsia spp. in Dermacentor and Haemaphysalis ticks from the Russian Far East. *Ticks Tick Borne Dis* 2018; **9**(6): 1594-603.
75. Kasama K, Fujita H, Yamamoto S, et al. Genomic Features of Rickettsia heilongjiangensis Revealed by Intraspecies Comparison and Detailed Comparison With Rickettsia japonica. *Front Microbiol* 2019; **10**: 2787.
76. Kholodilov IS, Belova OA, Morozkin ES, et al. Geographical and Tick-Dependent Distribution of Flavi-Like Alongshan and Yanggou Tick Viruses in Russia. *Viruses* 2021; **13**(3).
77. Aydin L, Bakirci S. Geographical distribution of ticks in Turkey. *Parasitol Res* 2007; **101 Suppl 2**: S163-66.
78. Rubel F, Brugger K, Walter M, et al. Geographical distribution, climate adaptation and vector competence of the Eurasian hard tick *Haemaphysalis concinna*. *Ticks Tick Borne Dis* 2018; **9**(5): 1080-89.
79. Duscher GG, Kübber-Heiss A, Richter B, Suchentrunk F. A golden jackal (Canis aureus) from Austria bearing Hepatozoon canis--import due to immigration into a non-endemic area? *Ticks Tick Borne Dis* 2013; **4**(1-2): 133-37.

80. Sukara R, Chochlakis D, Ćirović D, et al. Golden jackals (Canis aureus) as hosts for ticks and tick-borne pathogens in Serbia. *Ticks Tick Borne Dis* 2018; **9**(5): 1090-97.
81. Kovacevic J, Bucanovic T, Krcmar S. HARD TICK FAUNA (ACARI: IXODIDAE) IN DIFFERENT TYPES OF HABITATS IN THE CITY OF OSIJEK (EASTERN CROATIA). *Natura Croatica* 2020; **29**(1): 63-71.
82. Krčmar S. Hard ticks (Acari, Ixodidae) of Croatia. *Zookeys* 2012; (234): 19-57.
83. Keve G, Sándor AD, Hornok S. Hard ticks (Acari: Ixodidae) associated with birds in Europe: Review of literature data. *Front Vet Sci* 2022; **9**: 928756.
84. Foeldvari G, Marialigeti M, Solymosi N, et al. Hard ticks infesting dogs in Hungary and their infection with Babesia and Borrelia species. *Parasitology Research* 2007; **101**: S25-S34.
85. Radulovic Z, Mihaljica D, Cosic N, et al. Hard Ticks Parasitizing European Ground Squirrel, Spermophilus citellus (L.,1766) (Rodentia: Sciuridae) in Serbia. *Acta Zoologica Bulgarica* 2017; **69**(4): 547-53.
86. Borşan SD, Ionică AM, Galon C, et al. High Diversity, Prevalence, and Co-infection Rates of Tick-Borne Pathogens in Ticks and Wildlife Hosts in an Urban Area in Romania. *Front Microbiol* 2021; **12**: 645002.
87. Hornok S, Tánczos B, Fernández de Mera IG, de la Fuente J, Hofmann-Lehmann R, Farkas R. High prevalence of Hepatozoon-infection among shepherd dogs in a region considered to be free of Rhipicephalus sanguineus. *Vet Parasitol* 2013; **196**(1-2): 189-93.
88. Jia N, Zheng YC, Jiang JF, et al. Human Babesiosis Caused by a Babesia crassa-Like Pathogen: A Case Series. *Clin Infect Dis* 2018; **67**(7): 1110-19.
89. Li H, Jiang JF, Liu W, et al. Human infection with Candidatus Neoehrlichia mikurensis, China. *Emerg Infect Dis* 2012; **18**(10): 1636-39.
90. Ando S, Kurosawa M, Sakata A, et al. Human Rickettsia heilongjiangensis infection, Japan. *Emerg Infect Dis* 2010; **16**(8): 1306-08.
91. Faulde MK, Rutenfranz M, Hepke J, Rogge M, Görner A, Keth A. Human tick infestation pattern, tick-bite rate, and associated Borrelia burgdorferi s.l. infection risk during occupational tick exposure at the Seedorf military training area, northwestern Germany. *Ticks Tick Borne Dis* 2014; **5**(5): 594-99.
92. Egyed L, Rónai Z, Dán Á. Hungarian tick-borne encephalitis viruses isolated from a 0.5-ha focus are closely related to Finnish strains. *Ticks Tick Borne Dis* 2018; **9**(5): 1064-68.
93. Gaye M, Amanzougaghene N, Laidoudi Y, et al. Hymenopteran Parasitoids of Hard Ticks in Western Africa and the Russian Far East. *Microorganisms* 2020; **8**(12).
94. Ma J, Lv XL, Zhang X, et al. Identification of a new orthonairovirus associated with human febrile illness in China. *Nat Med* 2021; **27**(3): 434-39.
95. Pintér R, Madai M, Vadkerti E, et al. Identification of tick-borne encephalitis virus in ticks collected in southeastern Hungary. *Ticks Tick Borne Dis* 2013; **4**(5): 427-31.
96. Hornok S, Mulvihill M, Szőke K, et al. Impact of a freeway on the dispersal of ticks and Ixodes ricinus-borne pathogens: forested resting areas may become Lyme disease hotspots. *Acta Vet Hung* 2017; **65**(2): 242-52.
97. Spitalská E, Literák I, Kocianová E, Taragel'ová V. The importance of Ixodes arboricola in transmission of Rickettsia spp., Anaplasma phagocytophilum, and Borrelia burgdorferi sensu lato in the Czech Republic, Central Europe. *Vector Borne Zoonotic Dis* 2011; **11**(9): 1235-41.
98. Shchuchinova LD, Kozlova IV, Zlobin VI. Influence of altitude on tick-borne encephalitis infection risk in the natural foci of the Altai Republic, Southern Siberia. *Ticks Tick Borne Dis* 2015; **6**(3): 322-29.
99. Hornok S, Farkas R. Influence of biotope on the distribution and peak activity of questing ixodid ticks in Hungary. *Med Vet Entomol* 2009; **23**(1): 41-46.

100. Dyczko D, Kiewra D, Kolanek A, Błażej P. The influence of local environmental factors in southwestern Poland on the abundance of Ixodes ricinus and prevalence of infection with Borrelia burgdorferi s.l. and B. miyamotoi. *Parasitol Res* 2022; **121**(6): 1575-85.
101. Liu S, Yuan C, Cui YF, Li BX, Wu LJ, Liu Y. Investigation of Borrelia spp. in ticks (Acari: Ixodidae) at the border crossings between China and Russia in Heilongjiang Province, China. *Asian Pac J Trop Med* 2012; **5**(6): 459-64.
102. Hubálek Z, Halouzka J, Juricová Z. Investigation of haematophagous arthropods for borreliae--summarized data, 1988-1996. *Folia Parasitol (Praha)* 1998; **45**(1): 67-72.
103. Gyuranecz M, Rigó K, Dán A, et al. Investigation of the ecology of Francisella tularensis during an inter-epizootic period. *Vector Borne Zoonotic Dis* 2011; **11**(8): 1031-35.
104. Mediannikov O, Makarova V, Tarasevich I, Sidelnikov Y, Raoult D. Isolation of Rickettsia heilongjiangensis strains from humans and ticks and its multispacer typing. *Clin Microbiol Infect* 2009; **15 Suppl 2**: 288-89.
105. Thu MJ, Qiu Y, Kataoka-Nakamura C, et al. Isolation of Rickettsia, Rickettsiella, and Spiroplasma from Questing Ticks in Japan Using Arthropod Cells. *Vector Borne Zoonotic Dis* 2019; **19**(7): 474-85.
106. Mihalca AD, Gherman CM, Magdaş C, et al. Ixodes ricinus is the dominant questing tick in forest habitats in Romania: the results from a countrywide dragging campaign. *Exp Appl Acarol* 2012; **58**(2): 175-82.
107. Földvári G, Farkas R. Ixodid tick species attaching to dogs in Hungary. *Vet Parasitol* 2005; **129**(1-2): 125-31.
108. Bursali A, Tekin S, Orhan M, Keskin A, Ozkan M. Ixodid ticks (Acari: Ixodidae) infesting humans in Tokat Province of Turkey: species diversity and seasonal activity. *J Vector Ecol* 2010; **35**(1): 180-86.
109. Hornok S, Horváth G, Jongejan F, Farkas R. Ixodid ticks on ruminants, with on-host initiated moulting (apolysis) of Ixodes, Haemaphysalis and Dermacentor larvae. *Vet Parasitol* 2012; **187**(1-2): 350-53.
110. D'Amico G, Dumitrache MO, Matei IA, et al. Ixodid ticks parasitizing wild carnivores in Romania. *Exp Appl Acarol* 2017; **71**(2): 139-49.
111. Buczek A, Buczek W, Bartosik K, Kulisz J, Stanko M. Ixodiphagus hookeri wasps (Hymenoptera: Encyrtidae) in two sympatric tick species Ixodes ricinus and *Haemaphysalis concinna* (Ixodida: Ixodidae) in the Slovak Karst (Slovakia): ecological and biological considerations. *Sci Rep* 2021; **11**(1): 11310.
112. Cui Y, Yang G-Y, Bian Y. Karyotypic analyses of the bisexual and parthenogenetic tick (*Haemaphysalis concinna*). *International Journal of Acarology* 2012; **38**(3): 206-13.
113. Vogelgesang JR, Walter M, Kahl O, Rubel F, Brugger K. Long-term monitoring of the seasonal density of questing ixodid ticks in Vienna (Austria): setup and first results. *Exp Appl Acarol* 2020; **81**(3): 409-20.
114. Takada N, Ishiguro F, Fujita H, Wang HP, Wang JC, Masuzawa T. Lyme disease spirochetes in ticks from northeastern China. *J Parasitol* 1998; **84**(3): 499-504.
115. Wei F, Song M, Liu H, et al. Molecular Detection and Characterization of Zoonotic and Veterinary Pathogens in Ticks from Northeastern China. *Front Microbiol* 2016; **7**: 1913.
116. Wang J, Yang J, Liu J, et al. Molecular detection and genetic diversity of Theileria orientalis in cattle in China. *Parasitol Res* 2018; **117**(12): 3689-94.
117. Seo HJ, Noh J, Kim HC, et al. Molecular Detection and Phylogenetic Analysis of Anaplasma and Borrelia Species in Ticks Collected from Migratory Birds at Heuksan, Hong, and Nan Islands, Republic of Korea. *Vector Borne Zoonotic Dis* 2021; **21**(1): 20-31.
118. Mihaljica D, Radulovic Z, Tomanovic S, Cakic S, Penezic A, Milutinovic M. MOLECULAR DETECTION OF BABESIA SPP. IN TICKS IN NORTHERN SERBIA. *Archives of Biological Sciences* 2012; **64**(4): 1591-98.
119. Rahravani M, Moravedji M, Mostafavi E, et al. Molecular detection of Francisella tularensis in small ruminants and their ticks in western Iran. *Comp Immunol Microbiol Infect Dis* 2022; **83**: 101779.

120. Vrbová M, Belvončíková P, Kovaľová A, Matúšková R, Slovák M, Kúdelová M. Molecular detection of murine gammaherpesvirus 68 (MHV-68) in *Haemaphysalis concinna* ticks collected in Slovakia. *Acta Virol* 2016; **60**(4): 426-28.
121. Wang B, Sun LD, Liu HH, et al. Molecular detection of novel circoviruses in ticks in northeastern China. *Ticks Tick Borne Dis* 2018; **9**(4): 836-39.
122. Fuehrer HP, Biro N, Harl J, et al. Molecular detection of Theileria sp. ZS TO4 in red deer (Cervus elaphus) and questing *Haemaphysalis concinna* ticks in Eastern Austria. *Vet Parasitol* 2013; **197**(3-4): 653-57.
123. Hornok S, Boldogh SA, Takács N, et al. Molecular epidemiological study on ticks and tick-borne protozoan parasites (Apicomplexa: Cytauxzoon and Hepatozoon spp.) from wild cats (Felis silvestris), Mustelidae and red squirrels (Sciurus vulgaris) in central Europe, Hungary. *Parasit Vectors* 2022; **15**(1): 174.
124. Hornok S, Horváth G, Takács N, Farkas R, Szőke K, Kontschán J. Molecular evidence of a badger-associated Ehrlichia sp., a Candidatus Neoehrlichia lotoris-like genotype and Anaplasma marginale in dogs. *Ticks Tick Borne Dis* 2018; **9**(5): 1302-09.
125. Hornok S, Fuente J, Horváth G, et al. Molecular evidence of Ehrlichia canis and Rickettsia massiliae in ixodid ticks of carnivores from South Hungary. *Acta Vet Hung* 2013; **61**(1): 42-50.
126. Minichová L, Hamšíková Z, Mahríková L, et al. Molecular evidence of Rickettsia spp. in ixodid ticks and rodents in suburban, natural and rural habitats in Slovakia. *Parasit Vectors* 2017; **10**(1): 158.
127. Shpynov SN, Fournier PE, Rudakov NV, et al. Molecular identification of a collection of spotted Fever group rickettsiae obtained from patients and ticks from Russia. *Am J Trop Med Hyg* 2006; **74**(3): 440-43.
128. Del Cerro A, Oleaga A, Somoano A, Barandika JF, García-Pérez AL, Espí A. Molecular identification of tick-borne pathogens (Rickettsia spp., Anaplasma phagocytophilum, Borrelia burgdorferi sensu lato, Coxiella burnetii and piroplasms) in questing and feeding hard ticks from North-Western Spain. *Ticks Tick Borne Dis* 2022; **13**(4): 101961.
129. Hornok S, Meli ML, Perreten A, et al. Molecular investigation of hard ticks (Acari: Ixodidae) and fleas (Siphonaptera: Pulicidae) as potential vectors of rickettsial and mycoplasmal agents. *Vet Microbiol* 2010; **140**(1-2): 98-104.
130. Zöldi V, Reiczigel J, Egyed L. Monitoring the diel activity of Ixodes ricinus ticks in Hungary over three seasons. *Exp Appl Acarol* 2013; **61**(4): 509-17.
131. Hubálek Z, Halouzka J. Mosquitoes (Diptera: Culicidae), in contrast to ticks (Acari: Ixodidae), do not carry Francisella tularensis in a natural focus of tularemia in the Czech Republic. *J Med Entomol* 1997; **34**(6): 660-63.
132. Sparagano O, Foldvari G, Derdakova M, Kazimirova M. New challenges posed by ticks and tick-borne diseases. *Biologia* 2022; **77**(6): 1497-501.
133. Dwużnik D, Mierzejewska EJ, Alsarraf M, Bajer A. A new focus of the tick *Haemaphysalis concinna* in Western Poland. *Exp Appl Acarol* 2019; **78**(1): 93-112.
134. Wijnveld M, Schötta AM, Stelzer T, et al. Novel Protozoans in Austria Revealed through the Use of Dogs as Sentinels for Ticks and Tick-Borne Pathogens. *Microorganisms* 2021; **9**(7).
135. Rybarova M, Siroky P. Occurrence of Anaplasma phagocytophilum in three sympatric tick species in the South Moravia, Czech Republic. *Biologia* 2017; **72**(4): 365-69.
136. Dwużnik-Szarek D, Mierzejewska EJ, Bajer A. Occurrence of juvenile Dermacentor reticulatus ticks in three regions in Poland: the final evidence of the conquest. *Parasit Vectors* 2021; **14**(1): 536.
137. Hornok S, Meli ML, Gönczi E, et al. Occurrence of ticks and prevalence of Anaplasma phagocytophilum and Borrelia burgdorferi s.l. in three types of urban biotopes: forests, parks and cemeteries. *Ticks Tick Borne Dis* 2014; **5**(6): 785-89.
138. Špitalská E, Boldišová E, Štefanidesová K, et al. Pathogenic microorganisms in ticks removed from Slovakian residents over the years 2008-2018. *Ticks Tick Borne Dis* 2021; **12**(2): 101626.

139. Dwużnik-Szarek D, Mierzejewska EJ, Alsarraf M, Alsarraf M, Bajer A. Pathogens detected in the tick *Haemaphysalis concinna* in Western Poland: known and unknown threats. *Exp Appl Acarol* 2021; **84**(4): 769-83.
140. Hayasaka D, Suzuki Y, Kariwa H, et al. Phylogenetic and virulence analysis of tick-borne encephalitis viruses from Japan and far-Eastern Russia. *J Gen Virol* 1999; **80 (Pt 12)**: 3127-35.
141. Uspensky I, Ioffe-Uspensky I. Potential risk of pathogen transmission by acaricide-poisoned ticks. *Int J Med Microbiol* 2006; **296 Suppl 40**: 217-24.
142. Keskin A, Erciyas-Yavuz K. A Preliminary Investigation on Ticks (Acari: Ixodidae) Infesting Birds in Kızılırmak Delta, Turkey. *J Med Entomol* 2016; **53**(1): 217-20.
143. Chu CY, Jiang BG, Liu W, et al. Presence of pathogenic Borrelia burgdorferi sensu lato in ticks and rodents in Zhejiang, south-east China. *J Med Microbiol* 2008; **57**(Pt 8): 980-85.
144. Ma C, Zhang R, Zhou H, et al. Prevalence and genetic diversity of Dabieshan tick virus in Shandong Province, China. *J Infect* 2022; **85**(1): 90-122.
145. Wang Y, Li S, Wang Z, Zhang L, Cai Y, Liu Q. Prevalence and Identification of Borrelia burgdorferi Sensu Lato Genospecies in Ticks from Northeastern China. *Vector Borne Zoonotic Dis* 2019; **19**(5): 309-15.
146. Veronesi F, Galuppi R, Tampieri MP, Bonoli C, Mammoli R, Piergili Fioretti D. Prevalence of Anaplasma phagocytophilum in fallow deer (Dama dama) and feeding ticks from an Italy preserve. *Res Vet Sci* 2011; **90**(1): 40-43.
147. Pukhovskaya NM, Morozova OV, Vysochina NP, Belozerova NB, Ivanov LI. Prevalence of Borrelia burgdorferi sensu lato and Borrelia miyamotoi in ixodid ticks in the Far East of Russia. *Int J Parasitol Parasites Wildl* 2019; **8**: 192-202.
148. Hubálek Z, Stünzner D, Halouzka J, et al. Prevalence of borreliae in ixodid ticks from a floodplain forest ecosystem. *Wien Klin Wochenschr* 2003; **115**(3-4): 121-24.
149. Gyuranecz M, Dénes B, Hornok S, et al. Prevalence of Coxiella burnetii in Hungary: screening of dairy cows, sheep, commercial milk samples, and ticks. *Vector Borne Zoonotic Dis* 2012; **12**(8): 650-3.
150. Kreizinger Z, Hornok S, Dán A, et al. Prevalence of Francisella tularensis and Francisella-like endosymbionts in the tick population of Hungary and the genetic variability of Francisella-like agents. *Vector Borne Zoonotic Dis* 2013; **13**(3): 160-63.
151. Barandika JF, Hurtado A, García-Sanmartín J, Juste RA, Anda P, García-Pérez AL. Prevalence of tick-borne zoonotic bacteria in questing adult ticks from northern Spain. *Vector Borne Zoonotic Dis* 2008; **8**(6): 829-35.
152. Gurycová D, Kocianová E, Výrosteková V, Rehácek J. Prevalence of ticks infected with Francisella tularensis in natural foci of tularemia in western Slovakia. *Eur J Epidemiol* 1995; **11**(4): 469-74.
153. Rahbari S, Nabian S, Shayan P. Primary report on distribution of tick fauna in Iran. *Parasitol Res* 2007; **101 Suppl 2**: S175-7.
154. Kazimírová M, Hamšíková Z, Kocianová E, et al. Relative density of host-seeking ticks in different habitat types of south-western Slovakia. *Exp Appl Acarol* 2016; **69**(2): 205-24.
155. Chitimia-Dobler L, Lemhöfer G, Król N, Bestehorn M, Dobler G, Pfeffer M. Repeated isolation of tick-borne encephalitis virus from adult Dermacentor reticulatus ticks in an endemic area in Germany. *Parasit Vectors* 2019; **12**(1): 90.
156. Sréter-Lancz Z, Széll Z, Kovács G, Egyed L, Márialigeti K, Sréter T. Rickettsiae of the spotted-fever group in ixodid ticks from Hungary: identification of a new genotype ('Candidatus Rickettsia kotlanii'). *Ann Trop Med Parasitol* 2006; **100**(3): 229-36.
157. Barandika JF, Berriatua E, Barral M, Juste RA, Anda P, García-Pérez AL. Risk factors associated with ixodid tick species distributions in the Basque region in Spain. *Med Vet Entomol* 2006; **20**(2): 177-88.
158. Bellato A, Pintore MD, Catelan D, et al. Risk of tick-borne zoonoses in urban green areas: A case study from Turin, northwestern Italy. *Urban Forestry & Urban Greening* 2021; **64**.

159. Flaisz B, Hornok S. The role of birds in the eco-epidemiology of tick-borne pathogens. *Magyar Allatorvosok Lapja* 2017; **139**(8): 489-97.
160. Lak SS, Vatandoost H, Telmadarraiy Z, Mahdi RE, Kia EB. Seasonal Activity of Ticks and their Importance in Tick-Borne Infectious Diseases in West Azerbaijan, Iran. *Iranian Journal of Arthropod-Borne Diseases* 2008; **2**(2): 28-34.
161. Duscher GG, Feiler A, Leschnik M, Joachim A. Seasonal and spatial distribution of ixodid tick species feeding on naturally infested dogs from Eastern Austria and the influence of acaricides/repellents on these parameters. *Parasit Vectors* 2013; **6**: 76.
162. Davari B, Alam FN, Nasirian H, Nazari M, Abdigoudarzi M, Salehzadeh A. Seasonal distribution and faunistic of ticks in the Alashtar county (Lorestan Province), Iran. *Pan Afr Med J* 2017; **27**: 284.
163. Peňazziová K, Korytár Ľ, Cingeľová Maruščáková I, et al. Serologic Investigation on Tick-Borne Encephalitis Virus, Kemerovo Virus and Tribeč Virus Infections in Wild Birds. *Microorganisms* 2022; **10**(12).
164. Tian H, Yu P, Chowell G, et al. Severe Fever with Thrombocytopenia Syndrome Virus in Humans, Domesticated Animals, Ticks, and Mosquitoes, Shaanxi Province, China. *Am J Trop Med Hyg* 2017; **96**(6): 1346-49.
165. Reynolds C, Kontschán J, Takács N, et al. Shift in the seasonality of ixodid ticks after a warm winter in an urban habitat with notes on morphotypes of Ixodes ricinus and data in support of cryptic species within Ixodes frontalis. *Exp Appl Acarol* 2022; **88**(1): 127-38.
166. Dantas-Torres F, Otranto D. Species diversity and abundance of ticks in three habitats in southern Italy. *Ticks Tick Borne Dis* 2013; **4**(3): 251-55.
167. Leschnik MW, Khanakah G, Duscher G, et al. Species, developmental stage and infection with microbial pathogens of engorged ticks removed from dogs and questing ticks. *Med Vet Entomol* 2012; **26**(4): 440-46.
168. Mărcuţan ID, Kalmár Z, Ionică AM, et al. Spotted fever group rickettsiae in ticks of migratory birds in Romania. *Parasit Vectors* 2016; **9**(1): 294.
169. Rahbari S, Nabian S, Shayan P, Haddadzadeh HR. Status of Haemaphysalis tick infestation in domestic ruminants in Iran. *Korean J Parasitol* 2007; **45**(2): 129-32.
170. Dautel H, Knülle W. The supercooling ability of ticks (Acari, Ixodoidea). *J Comp Physiol B* 1996; **166**(8): 517-24.
171. Andoh M, Andoh R, Teramoto K, et al. Survey of Coxiella burnetii in ticks collected from dogs in Japan. *J Vet Med Sci* 2013; **75**(8): 1115-17.
172. Li J, Zhang S, Liang W, et al. Survey of tick species and molecular detection of selected tick-borne pathogens in Yanbian, China. *Parasite* 2022; **29**: 38.
173. Răileanu C, Tauchmann O, Silaghi C. Sympatric occurrence of Ixodes ricinus with Dermacentor reticulatus and *Haemaphysalis concinna* and the associated tick-borne pathogens near the German Baltic coast. *Parasit Vectors* 2022; **15**(1): 65.
174. Svehlová A, Berthová L, Sallay B, Boldiš V, Sparagano OA, Spitalská E. Sympatric occurrence of Ixodes ricinus, Dermacentor reticulatus and *Haemaphysalis concinna* ticks and Rickettsia and Babesia species in Slovakia. *Ticks Tick Borne Dis* 2014; **5**(5): 600-05.
175. Mihalca AD, Dumitrache MO, Magdaş C, et al. Synopsis of the hard ticks (Acari: Ixodidae) of Romania with update on host associations and geographical distribution. *Exp Appl Acarol* 2012; **58**(2): 183-206.
176. Karatas A. A systematic investigation on the ticks (Acari: Ixodida) of the domestic sheep in Nide Province, Turkey. *Journal of Wildlife and Biodiversity* 2020; **4**(4): 31-40.
177. Weiler M, Duscher GG, Wetscher M, Walochnik J. Tick abundance: a one year study on the impact of flood events along the banks of the river Danube, Austria. *Exp Appl Acarol* 2017; **71**(2): 151-57.
178. Sheng J, Jiang M, Yang M, et al. Tick distribution in border regions of Northwestern China. *Ticks Tick Borne Dis* 2019; **10**(3): 665-69.

179. Polat E, Altinkum SM, Bagdatli Y, Baykara O. The tick fauna in Istanbul, Turkey, from 2013 to 2017 and identification of their pathogens by multiplex PCR: an epidemiological study. *Exp Appl Acarol* 2021; **84**(4): 825-34.
180. Kahl O, Janetzki C, Gray JS, Stein J, Bauch RJ. Tick infection rates with Borrelia: Ixodes ricinus versus *Haemaphysalis concinna* and Dermacentor reticulatus in two locations in eastern Germany. *Med Vet Entomol* 1992; **6**(4): 363-66.
181. Aydin MF, Aktas M, Dumanli N. Tick Infestations on Sheep and Goats in the Black Sea Region of Turkiye. *Kafkas Universitesi Veteriner Fakultesi Dergisi* 2012; **18**: A17-A22.
182. Pukhovskaya NM, Morozova OV, Vysochina NP, et al. Tick-borne encephalitis virus in arthropod vectors in the Far East of Russia. *Ticks Tick Borne Dis* 2018; **9**(4): 824-33.
183. Andersson MO, Marga G, Banu T, Dobler G, Chitimia-Dobler L. Tick-borne pathogens in tick species infesting humans in Sibiu County, central Romania. *Parasitol Res* 2018; **117**(5): 1591-97.
184. Shpynov S, Fournier PE, Rudakov N, et al. Tick-borne rickettsiosis in the Altay region of Russia. *Clin Microbiol Infect* 2009; **15 Suppl 2**: 313-14.
185. Barandika JF, Hurtado A, García-Esteban C, et al. Tick-borne zoonotic bacteria in wild and domestic small mammals in northern Spain. *Appl Environ Microbiol* 2007; **73**(19): 6166-71.
186. Keskin A, Erciyas-Yavuz K. Ticks (Acari: Ixodidae) Parasitizing Passerine Birds in Turkey With New Records and New Tick-Host Associations. *J Med Entomol* 2019; **56**(1): 156-61.
187. Karbowiak G, Stanko M, Miterpaková M, Hurníková Z, Víchová B. Ticks (Acari: Ixodidae) Parasitizing Red Foxes (Vulpes vulpes) in Slovakia and New Data About Subgenus Pholeoixodes Occurrence. *Acta Parasitol* 2020; **65**(3): 636-43.
188. Kaiser MN, Hoogstra.H, Watson GE. TICKS (IXODOIDEA) ON MIGRATING BIRDS IN CYPRUS, FALL 1967 AND SPRING 1968, AND EPIDEMIOLOGICAL CONSIDERATIONS. *Bulletin of Entomological Research* 1974; **64**(1): 97-110.
189. Hassl AR. Ticks and mites parasitizing free-ranging reptiles in Austria with an identification key to Central European herpetophagous Acarina. *Herpetozoa* 2016; **29**(1-2): 77-83.
190. Stanko M, Derdáková M, Špitalská E, Kazimírová M. Ticks and their epidemiological role in Slovakia: from the past till present. *Biologia (Bratisl)* 2022; **77**(6): 1575-610.
191. Choi CY, Kang CW, Kim EM, et al. Ticks collected from migratory birds, including a new record of Haemaphysalis formosensis, on Jeju Island, Korea. *Exp Appl Acarol* 2014; **62**(4): 557-66.
192. Rubel F, Dautel H, Nijhof AM, Kahl O. Ticks in the metropolitan area of Berlin, Germany. *Ticks Tick Borne Dis* 2022; **13**(6): 102029.
193. Ciebiera O, Łopińska A, Gabryś G. Ticks on game animals in the fragmented agricultural landscape of western Poland. *Parasitol Res* 2021; **120**(5): 1781-88.
194. Rasulov I. Ticks status in Central Asia with a special emphasis on Uzbekistan. *Parasitol Res* 2007; **101 Suppl 2**: S183-86.
195. Heglasová I, Rudenko N, Golovchenko M, Zubriková D, Miklisová D, Stanko M. Ticks, fleas and rodent-hosts analyzed for the presence of Borrelia miyamotoi in Slovakia: the first record of Borrelia miyamotoi in a Haemaphysalis inermis tick. *Ticks Tick Borne Dis* 2020; **11**(5): 101456.
196. Sándor AD, Kalmár Z, Matei I, Ionică AM, Mărcuţan ID. Urban Breeding Corvids as Disseminators of Ticks and Emerging Tick-Borne Pathogens. *Vector Borne Zoonotic Dis* 2017; **17**(2): 152-54.
197. Rybarova M, Honsova M, Papousek I, Siroky P. Variability of species of Babesia Starcovici, 1893 in three sympatric ticks (Ixodes ricinus, Dermacentor reticulatus and *Haemaphysalis concinna*) at the edge of Pannonia in the Czech Republic and Slovakia. *Folia Parasitol (Praha)* 2017; **64**.
198. Sonnberger BW, Wortha LN, Rackl D, Obwaller AG, Joachim A, Fuehrer HP. Vector Surveillance and Pathogen Detection in the Working Areas of Military Working Dogs in Eastern Austria. *Pathogens* 2022; **11**(5).

199. Cai X, Cai X, Xu Y, et al. Virome analysis of ticks and tick-borne viruses in Heilongjiang and Jilin Provinces, China. *Virus Res* 2022; **323**: 199006.
200. Sameroff S, Tokarz R, Vucelja M, et al. Virome of Ixodes ricinus, Dermacentor reticulatus, and *Haemaphysalis concinna* Ticks from Croatia. *Viruses* 2022; **14**(5).

201. 邓绍生. 2008 年苍溪县嗜群血蜱流行情况调查. *华南预防医学* 2009; **35**(03): 48-9+51.
202. 韩辉, 吴海磊, 胡学锋, 宋亚京, 徐宝梁. 2012—2014 年黑龙江口岸蜱携带病原体调查. *中国国境卫生检疫杂志* 2016; **39**(06): 413-6.
203. 朱金国, 徐连贵, 陈美超, 等. 2015 年额尔古纳口岸及毗邻林区蜱类调查. *中国国境卫生检疫杂志* 2016; **39**(05): 336-8.
204. 崔云虹, 刘丹, 乌兰图雅, 等. 2016—2019 年内蒙古大兴安岭林区立克次体关联细菌群监测. *现代预防医学* 2021; **48**(15): 2850-6.
205. 常瑾. 埃里希氏体和伯氏疏螺旋体双重荧光定量 PCR 检测方法的建立和初步应用. 吉林大学; 2021.
206. 杨银书, 王祥生, 李德昌. 安徽省首次发现嗜群血蜱. *兽医大学学报* 1990; (01): 24.
207. 鲜升文, 陈大林, 文兴诚, 罗北平, 谭永孝. 巴中市嗜群血蜱疫情调查. *中国兽医杂志* 2002; (02): 24-5.
208. 马博, 唐莉娟, 刘丹, 卜三平. 巴州部分地区羊体表蜱类调查及防控措施. *黑龙江畜牧兽医* 2021; (03): 99-102.
209. 蒋玉曦. 巴州塔里木马鹿蜱种类鉴定及其携带部分病原检测. 塔里木大学; 2020.
210. 娄丹, 吴益民, 王冰, 等. 斑点热立克次体的一个新成员——黑龙江立克次体的分离和鉴定. *中华微生物学和免疫学杂志* 1985; **05**(4): 250-3.
211. 张健之，范明远，毕德增，宋秀萍. 斑点热群立克次体新种──虎林－93 株的分离和鉴定. *中国人兽共患病杂志* 1996; (05): 2-8.
212. 胡晓倩, 曾令霞, 田辉, 等. 宝鸡市新型布尼亚病毒感染状况调查. *西安交通大学学报(医学版)* 2015; **36**(04): 571-3.
213. 王燕春. 北极内罗病毒的发现及流行病学研究. 吉林农业大学; 2021.
214. 李建民, 曹务春, 张习坦, 等. 北京林区蜱类及鼠类调查. *中国媒介生物学及控制杂志* 2002; (03): 165-8.
215. 吴益民, 刘国平, 魏安明, 等. 部分地区蜱传斑点热自然疫源地调查. *中国公共卫生* 2003; (09): 25-6.
216. 于昊江. 昌吉部分地区牛感染蜱传绵羊无浆体的情况调查. 新疆农业大学; 2019.
217. 陈联宏, 阿依肯, 赵明. 昌吉州蜱类群落组成和分布. *中华卫生杀虫药械* 2017; **23**(04): 376-7.
218. 李志清, 刘增加, 上官改珍, 等. 巢式-PCR 检测蜱体中土拉弗氏菌的 fopA 基因及血清学调查. *寄生虫与医学昆虫学报* 2014; **21**(3): 160-4.
219. 王春仁. 春季放牧家畜应预防硬蜱侵袭. *中国畜牧兽医文摘* 2005; (02): 28+7.
220. 窦桂兰, 姚逸红, 张哲夫, 曹伯良, 万康林. 从嗜群血蜱观察到莱姆螺旋体. *中国鼠类防制杂志* 1988; (01): 18.
221. 蹇锐, 温博海, 张有植, 陈荣. 从西藏微小牛蜱检出类查菲埃立克体和边缘无形体 16S rDNA. *中国人兽共患病杂志* 2002; (03): 39-41+85.
222. 孙林山. 大兴安岭地区蜱携带巴贝斯虫及人感染巴贝斯虫病调查研究. 内蒙古民族大学; 2019.
223. 褚宸一, 何静, 王剑波, 等. 大兴安岭林区蜱和鼠中莱姆病螺旋体感染及其基因分型研究. *中华流行病学杂志* 2006; (08): 681-4.
224. 刘国平, 杨国平, 任清明, 王峰, 武春光. 东北边境地区鼠体外寄生蜱螨的种类组成研究. *蛛形学报* 2008; (02): 106-10.
225. 魏安明, 胡玲美, 吴益民, 等. 东北部分地区蜱传埃立克体 DNA 的检测. *解放军预防医学杂志* 2004; (06): 430-3.

226. 吴琼, 冯立, 王洪军, 等. 东北部分地区蜱携带几种病原立克次体的分子检测. *中国预防医学杂志* 2012; **13**(12): 892-4.
227. 王卓, 王建伟, 于淼, 等. 东北地区蜱传斑点热群立克次体的分子流行病学研究. *中国媒介生物学及控制杂志* 2018; **29**(04): 344-7.
228. 李忠宇, 刘欢欢, 刘全, 马宏宇, 魏峰. 东北地区蜱携带的肝原虫分子检测. *中国兽医学报* 2018; **38**(09): 1720-4.
229. 马宏宇. 东北地区蜱携带新型病毒的发现及鉴定. 吉林农业大学; 2017.
230. 蔡增林，鲁志新，胡玲美，赵占林，金显涛，何亦祥. 东北三省部份地区森林脑炎疫源地流行病学调查. *微生物学杂志* 1996; (01): 19-22.
231. 刘国平, 任清明, 王峰, 孙薇, 韩晓娜. 东北中朝俄边境地区蜱的生态及防制对策研究. *中华卫生杀虫药械* 2006; (03): 208-10.
232. 刘国平, 任清明, 王峰. 东北中俄边境地区黑线姬鼠及体外寄生虫的调查研究. *中华卫生杀虫药械* 2006; (06): 465-7.
233. 张健之，范明远，毕德增，孙福祥，程昭祥，兰福春，崔文富，孙秀英，只相国，林碧瑚. 多聚酶链反应技术在斑点热群立克次体流行病学调查中的应用. *中华流行病学杂志* 1995; (01): 25-8.
234. 李志锋. 发热伴血小板减少综合征病毒传播机制及炎症小体和细胞焦亡在其感染过程中的作用研究. 南京大学; 2020.
235. 庄麟春，吴丽骠，徐洪业，张德才，张仲秋，孙广久. 抚顺县莱姆病流行病学调查. *中国公共卫生学报* 1994; (05): 259.
236. 孟菲. 广西蝙蝠和大兴安岭蜱虫的宏病毒组学研究. 广西大学; 2019.
237. 刘联坤, 郭建军. 贵州禽畜寄生蜱种类及优势种. *西南师范大学学报(自然科学版)* 2011; **36**(01): 98-101.
238. 刘联坤. 贵州省畜禽寄生蜱分类研究. 贵州大学; 2010.
239. 华满堂, 金兆清, 宫占威, 王志亭, 何成. 哈巴河铁热克提林牧区常见蜱类携带伯氏疏螺旋体调查. *中国人兽共患病杂志* 2000; (01): 110-05.
240. 刘国平, 年春志, 贺顺喜, 等. 哈尔巴岭重要医学动物及防制对策. *中华卫生杀虫药械* 2005; (05): 323-5.
241. 宋春玲. 哈尔滨五个自然风景区蜱及蜱传病原的调查. 黑龙江八一农垦大学; 2018.
242. 贾月萍, 周国萍, 张晓芳, 等. 黑河地区小兴安岭林场首次分离培养出伯氏疏螺旋体. *中国预防医学杂志* 2003; (01): 57-8.
243. 叶楠楠, 裴希超, 孙善华. 黑河口岸地区蜱类调查及研究. *中国人兽共患病学报* 2018; **34**(08): 761-7.
244. 蔡祥龙. 黑龙江、吉林省部分地区蜱传病毒分子流行病学研究. 东北农业大学; 2020.
245. 付雪, 宋春玲, 常巧呈, 王春仁. 黑龙江不同地点嗜群血蜱核糖体和线粒体基因遗传变异分析. *黑龙江八一农垦大学学报* 2018; **30**(02): 25-9+60.
246. 范东辉, 呼满霞, 何浩, 等. 黑龙江东部七口岸蜱种群特征及携带莱姆病病原体的研究. *口岸卫生控制* 2006; (05): 40-2.
247. 鞠文东, 王艳梅, 徐宁, 等. 黑龙江黑河口岸鼠类及蜱类携带巴尔通体的调查. *口岸卫生控制* 2018; **23**(02): 60-3.
248. 孙秀峰, 丁淑丽, 呼满霞, 郭晓明, 赵刚. 黑龙江口岸蜱类斑点热群立克次体感染状况初探. *中国国境卫生检疫杂志* 2007; (03): 154-6.
249. 杨丽炜, 侯咏, 李明, 温占清, 杨军, 范东辉. 黑龙江口岸蜱类及其携带病原体状况的初步研究. *中国国境卫生检疫杂志* 2007; (02): 77-82.
250. 杨丽炜, 侯咏, 石杨, 等. 黑龙江口岸蜱类及其携带病原体状况研究初报. *中国国境卫生检疫杂志* 2009; **32**(05): 354-62.
251. 程成. 黑龙江口岸蜱媒病原的多样性及其复合感染调查. 中国人民解放军军事医学科学院; 2017.

252. 程成, 鞠文东, 王艳梅, 等. 黑龙江口岸蜱携带斑点热群立克次体及嗜吞噬细胞无形体复合感染调查. *口岸卫生控制* 2019; **24**(02): 53-9.
253. 程成, 鞠文东, 焦丹, 等. 黑龙江口岸新发蜱传疾病的调查. *中国国境卫生检疫杂志* 2015; **38**(03): 176-81.
254. 唐琨. 黑龙江林区蜱和鼠中三种病原的携带状况研究. 中南大学; 2012.
255. 程成, 鞠文东, 付维明, 等. 黑龙江密山口岸三种蜱媒传染病在媒介蜱中复合感染的调查. *中国公共卫生管理* 2015; **31**(03): 361-3.
256. 杨丽炜, 郭晓明, 李国军, 等. 黑龙江省 11 个边境口岸蜱类调查. *中国国境卫生检疫杂志* 2006; (05): 295-7.
257. 何浩, 郝琴, 呼满霞, 等. 黑龙江省东部边境口岸莱姆病生物媒介调查及病原分型研究. *中华流行病学杂志* 2007; **28**(1): 70-3.
258. 蔡增林. 黑龙江省东宁县蜱传斑点热自然疫源地调查报告. *中国人兽共患病杂志* 1986; (02): 35-7.
259. 陈侠, 牛磊, 梁慧杰, 裴立君, 栾鹏, 孙天航. 黑龙江省抚远口岸 2019—2021 年蜱类及其携带病原体调查. *中国国境卫生检疫杂志* 2022; **45**(05): 357-60.
260. 李樊, 刘凤明, 冯盼盼, 等. 黑龙江省桦南县 2020 年蜱媒病毒调查. *中国媒介生物学及控制杂志* 2022; **33**(05): 637-41.
261. 付维明, 丁大伟, 李明, 崔忠起, 赵伟, 崔永民. 黑龙江省口岸地区蜱类感染嗜吞噬细胞无形体 Anaplasma phagocytophila 的初步调查. *寄生虫与医学昆虫学报* 2010; **17**(3): 152-6.
262. 唐琨, 左双燕, 李颖, 等. 黑龙江省旅游区蜱伯氏疏螺旋体和斑点热群立克次体复合感染的动态调查. *中华流行病学杂志* 2012; **33**(5): 513-6.
263. 王晓宇, 马玉杰, 孙兆丹, 等. 黑龙江省蜱虫分布及蜱传脑炎病毒携带情况调查. *中国公共卫生管理* 2020; **36**(05): 750-2.
264. 罗金, 李静, 张高峰, 等. 黑龙江省三地蜱体内 Q 热病原的感染情况和分子特征. *中国兽医科学* 2021; **51**(11): 1385-91.
265. 惠国隆. 黑龙江省小兴安岭莱姆病调查研究. *哈尔滨医药* 1997; (01): 24-6.
266. 梁慧杰, 王玉梅, 程成, 王思哲, 石洪兴. 黑龙江省中俄边境口岸 2019—2020 年蜱类及其携带病原体监测. *中国口岸科学技术* 2021; **3**(10): 17-22.
267. 崔文富, 张少华, 呼满霞, 等. 黑龙江省中俄边境七个口岸蜱类调查. *中国媒介生物学及控制杂志* 2003; (05): 380.
268. 呼满霞, 井源, 张爽, 张兆鑫, 范东辉. 黑龙江绥芬河口岸重要媒介生物监测. *中国国境卫生检疫杂志* 2015; **38**(06): 386-9.
269. 蔡祥龙, 闫晓敏, 董馨, 何彪, 朱妍. 黑龙江与吉林的部分地区蜱传黄病毒的检测与鉴定. *中国预防兽医学报* 2020; **42**(11): 1116-22.
270. 付维明, 温占清, 杨军, 鞠文东. 黑龙江中俄边境地区莱姆病螺旋体的主要动物宿主及媒介生物调查. *口岸卫生控制* 2009; **14**(01): 28-35.
271. 何浩, 张国斌, 呼满霞, 鞠文东. 黑龙江中俄边境地区莱姆病螺旋体基因型调查及病原分型研究. 第二届媒介生物可持续控制国际论坛; 2008; 中国北京; 2008. p. 301-5.
272. 富英群, 卢婷婷, 侯咏, 等. 黑瞎子岛地区蜱及其携带病原体调查研究. *中国国境卫生检疫杂志* 2015; **38**(02): 119-23.
273. 赵晓东, 李世林, 何小强, 等. 红原县龙日坝地区蜱类调查研究. *草学* 2021; (05): 73-5.
274. 李枝金, 刘亦仁, 刘立屏, 董美阶, 向恒阳. 湖北宜昌市蜱类调查及区系研究. *中国媒介生物学及控制杂志* 2002; (03): 200-1.
275. 穆翠英, 赵娜, 罗公军, 等. 珲春口岸地区鼠蚊蜱的调查. *中国国境卫生检疫杂志* 2015; **38**(S1): 30-3.
276. 王峰, 刘国平, 任清明, 等. 吉林敦化林区蜱类调查. *中华卫生杀虫药械* 2005; (05): 321-2.
277. 浦昀, 李娟, 宋秀环, 等. 吉林口岸地区主要蜱类及蜱媒病原检测分析. *寄生虫与医学昆虫学报* 2009; **16**(4): 228-32.

278. 任清明, 王峰, 王旭, 刘国平, 邵锴. 吉林省敦化市山林地区重要医学节肢动物的调查研究. *中国媒介生物学及控制杂志* 2015; **26**(05): 516-8.
279. 陶增珧, 杨成君, 刘希真, 等. 吉林省抚松、珲春地区莱姆病调查. *中国公共卫生学报* 1991; (01): 15-7.
280. 刘欢欢. 吉林省和黑龙江省蜱种分布及 3 种重要蜱传微生物分子流行病学调查. 吉林农业大学; 2017.
281. 杨修军, 万康林, 王春生, 等. 吉林省莱姆病病原学实验室研究. *中国卫生检验杂志* 2006; (11): 1355-6+62.
282. 李基旭, Cho S-H, Kim S-Y, 等. 吉林省图们江流域常见蜱类调查研究. *中国媒介生物学及控制杂志* 2020; **31**(04): 446-50.
283. 李基旭, YUN S-M, KIM S-Y, 等. 吉林省延边地区蜱类分布及其携带发热伴血小板减少综合征病毒调查. *寄生虫与医学昆虫学报* 2017; **24**(02): 132-40.
284. 郝永建, 曹务春, 高淑萍, 等. 吉林省长白山区斑点热立克次体自然疫源地调查. *中华流行病学杂志* 2003; (12): 62-4.
285. 沈博, 李基旭, 黄飚, 等. 吉林省长白山区蜱类调查研究. *中国媒介生物学及控制杂志* 2020; **31**(06): 667-71.
286. 李基旭, 朴文, 金永善, 等. 吉林延边地区边境县蜱和牛携带东方泰勒虫感染调查. *寄生虫与医学昆虫学报* 2022; **29**(02): 67-71+97.
287. 李基旭, 赵鑫, 金光星, 等. 吉林延边地区蜱复合感染新塔拉塞维奇立克次体及 SFTSV 调查研究. *中华疾病控制杂志* 2019; **23**(05): 561-6+72.
288. 韩茜, 许红彬, 冯云, 等. 江西省赣州市蜱携带立克次体和埃立克体的调研. *中国人兽共患病学报* 2019; **35**(06): 518-24.
289. 李连峰. 荆门蜱病毒分子流行病学调查和经媒介蜱传播规律的研究. 广西医科大学; 2020.
290. 刘明社, 黄克峻, 赵中夫, 张联珠, 郭佩如. 九省区蜱类区系初步调查. *长治医学院学报* 2005; (04): 249-50.
291. 牟路萌. 莱姆病、布鲁菌病诊断方法的建立和流行病学调查. 石河子大学; 2016.
292. 邵建伟. 两类重要人兽共患病病原体在主要动物宿主中的分布、传播风险及其分子特征研究. 浙江大学; 2020.
293. 黎浩. 两种虫媒传染病自然疫源地调查与病原特征研究. 中国人民解放军军事医学科学院; 2013.
294. 张德才, 洪源, 张仲秋, 等. 辽宁从蜱、鼠中分离到伯氏疏螺旋体. *中国媒介生物学及控制杂志* 1992; (01): 19.
295. 于东海. 辽宁省部分地区蜱种分布及携带主要病原的调查研究. 沈阳农业大学; 2020.
296. 刘明社. 鹿泉沟自然保护区蜱类及蜱叮人情况初步调查. *长治医学院学报* 1995; (03): 199-200.
297. 褚宸一. 媒介和宿主中伯氏疏螺旋体感染调查及基因分型研究. 中国人民解放军军事医学科学院; 2009.
298. 魏然. 绵羊无形体自然疫源地调查及其对人致病性研究. 中国人民解放军军事医学科学院; 2017.
299. 郝永建, 曹务春, 高淑萍, 等. 某部驻区蜱传斑点热自然疫源地调查. *疾病控制杂志* 2005; (03): 243-5.
300. 郝永建, 宋世佩, 高淑萍, 李旭升. 某部驻区蜱类和鼠类调查. *医学动物防制* 2002; (12): 672-4.
301. 韩华, 石华, 王玥, 等. 牡丹江镜泊湖区蜱中伯氏疏螺旋体与嗜吞噬细胞无形体感染的研究. *中华卫生杀虫药械* 2015; **21**(02): 154-7.
302. 于红敏, 南晓伟, 司晓艳, 等. 内蒙古边境口岸地区 2019 年蜱种调查. *中国媒介生物学及控制杂志* 2022; **33**(02): 289-92.
303. 高东旗, 曹务春, 张习坦, 赵秋敏, 朱建华. 内蒙古大兴安岭林区人埃立克体病自然疫源地的调查. *传染病信息* 2001; (04): 168-70.
304. 李华, 张晓光. 内蒙古大兴安岭林区森林脑炎流行现状. *中华预防医学杂志* 1999; (03): 21.

305. 王淑英，李炎光，任国松，阎大成，. 内蒙古大兴安岭林区森林脑炎自然疫源地调查报告. *地方病通报* 1995; (02): 72-4.
306. 阎大成，李炎光，王淑英，陈露菲，任国松，刘彦成. 内蒙古大兴安岭林区首次分离出森林脑炎病毒. *内蒙古医学杂志* 1996; (02): 65-7.
307. 刘丹, 乌兰图雅, 殷旭红, 等. 内蒙古大兴安岭林区牙克石段的蜱种及蜱携病原体感染调查. *中国人兽共患病学报* 2021; **37**(09): 845-50+57.
308. 刘丹, 乌兰图雅, 殷旭红, 等. 内蒙古地区2015—2019年不同寄生蜱的种群分布及病原体多样性分析. *现代预防医学* 2021; **48**(08): 1345-9+98.
309. 田雪斌, 阮青青, 朱金贵, 徐连贵, 陈美超, 许士奇. 内蒙古额尔古纳国家级自然保护区蜱类种群调查. *兽医导刊* 2019; (2): 38-9.
310. 周磊. 内蒙古林区媒介蜱携带病原体的调查研究. 中国人民解放军军事医学科学院; 2017.
311. 盛悦. 内蒙古蜱传斑点热群立克次体分子流行特征研究. 承德医学院; 2021.
312. 汤芳, 周磊, 蒋兰芬, 栾进, 浮飞翔. 内蒙古奇乾地区媒介蜱携带伯氏疏螺旋体的调查研究. *传染病信息* 2018; **31**(01): 31-3.
313. 周磊, 汤芳, 栾进, 刘玮. 内蒙古奇乾地区蜱携带斑点热立克次体的调查. *中国国境卫生检疫杂志* 2017; **40**(02): 96-9.
314. 路瑾萍，叶长香，杨秀峰，李庆安，韩效忠，张哲夫，王宏英，侯学霞，郑理，赵燕平，宋瑞，张国华，岳凤芹. 内蒙古锡盟宝格达山林区人群莱姆病调查. *中国人兽共患病杂志* 1996; (01): 59.
315. 秦文浩, 李玉芝, 宋彩华. 内蒙古牙克石地区蜱消长调查. *中国人兽共患病杂志* 2005; (11): 11.
316. 阮青青, 田雪斌, 朱金国, 徐连贵, 陈美超, 许士奇. 内蒙古中俄边境口岸地区蜱类调查. *寄生虫与医学昆虫学报* 2019; **26**(01): 65-8.
317. 李志刚, 马福海, 吴美云, 等. 宁夏蜱类种群及分布. *中国媒介生物学及控制杂志* 1998; (01): 52-3.
318. 郭雨微. 蜱传病中 Q 热和北亚热检测方法建立及东北内蒙地区疫病调查. 吉林农业大学; 2013.
319. 魏嘉良. 蜱传媒病中鼠疫、野兔热病原检测方法的建立及疫病情况调查. 吉林农业大学; 2013.
320. 刘洪波. 蜱感染病原体的分离鉴定与监测研究. 军事科学院; 2018.
321. 张雅明. 蜱媒疾病的影响因素调查及其综合评价研究. 哈尔滨医科大学; 2014.
322. 石华, 王玥, 韩华, 等. 蜱媒疾病风险评估中标本采集方法的探讨. *中华卫生杀虫药械* 2013; **19**(04): 308-10.
323. 张志强, 吴益民, 冯立, 等. 蜱中人粒细胞埃立克体 DNA 的 PCR 检测及序列分析. *中国人兽共患病学报* 2007; (03): 266-8.
324. 孙家刚, 李开均, 魏洪, 何忠武. 禽等动物感染嗜群血蜱的研究. 自主创新与持续增长第十一届中国科协年会; 2009; 中国重庆; 2009. p. 363-8.
325. 薛晓宁, 侯伟, 孙宝杰. 青岛机场口岸医学媒介生物本底调查. *中国国境卫生检疫杂志* 2009; **32**(01): 22-6+34.
326. 孙宝杰, 薛晓宁, 侯伟. 青岛流亭国际机场蜱、螨本底调查报告. *口岸卫生控制* 2009; **14**(06): 39-41.
327. 蔡东浩，何亚轩，王金生，傅有为，于德泗，才运亨，张德才，张仲秋，孙广久. 清原县莱姆病自然疫源地调查. *中国媒介生物学及控制杂志* 1994; (05): 382-3.
328. 魏然, 江佳富, 蒋宝贵, 等. 全沟硬蜱、森林革蜱及嗜群血蜱经卵传播新塔拉塞维奇立克次体的检测研究. *寄生虫与医学昆虫学报* 2017; **24**(01): 19-24.
329. 万康林, 张哲夫, 窦桂兰, 等. 全沟硬蜱是我国北方地区莱姆病螺旋体的主要传播媒介. *实用预防医学* 1998; (06): 3-6.
330. 高杨, 许士奇, 王丽娜, 郭文平, 刘铭. 塞罕坝自然保护区蜱媒斑点热群立克次体检测及遗传进化分析. *中国媒介生物学及控制杂志* 2022; **33**(02): 252-7.
331. 高杨, 许士奇, 李丽静, 王丽娜, 刘铭. 塞罕坝自然保护区硬蜱调查及分子鉴定. *中国病原生物学杂志* 2019; **14**(09): 1054-7+75.
332. 高旭, 许应天. 瑟氏泰勒虫病诊断技术的研究进展. *延边大学农学学报* 2005; (02): 147-52.

333. 张丽娟, 崔峰, 王玲, 等. 山东省沂源县无形体病实验室调查分析. *传染病信息* 2009; **22**(01): 21-5.
334. 侯学霞, 张琳, 李国华, 郝琴. 山西省吕梁地区莱姆病自然疫源地调查研究. *中国媒介生物学及控制杂志* 2017; **28**(06): 550-2.
335. 田庆云. 山西省血蜱新记录——嗜群血蜱. *山西医学院学报* 1989; (02): 128.
336. 郑龙. 陕西省部分地区土拉弗朗西斯菌病自然疫源地调查研究. *西北国防医学杂志* 2018; **39**(07): 426-31.
337. 杨银书, 曹健, 赵红斌, 张继军, 第五进学, 高小莉. 陕西省蜱的种类与自然地理分布. *中华卫生杀虫药械* 2008; (02): 97-9.
338. 李志清, 刘增加, 宫占威, 等. 陕西省蜱莱姆病螺旋体分子流行病学研究. *寄生虫与医学昆虫学报* 2010; **17**(3): 148-51.
339. 刘钊. 嗜群血蜱共生菌的研究. 河北师范大学; 2014.
340. 古小彬, 余增莹, 杨光友, 等. 嗜群血蜱和长角血蜱 ITS-2、COI和 COII基因序列变异与亲缘关系分析. *畜牧兽医学报* 2010; **41**(06): 746-54.
341. 孟浩. 嗜群血蜱种群生态及美洲花蜱防治的研究. 河北师范大学; 2015.
342. 任国松, 李炎光, 阎大成, 等. 首次从全沟硬蜱中分离 3 株森林脑炎病毒. *医学动物防制* 1994; (02): 71-3.
343. 范东辉, 李明, 徐翮飞, 呼满霞, 张箭, 孙毅. 鼠与蜱感染人致病性巴贝虫状况的初步研究. *中华卫生杀虫药械* 2012; **18**(01): 48-50.
344. 陈东平, 周曼珠, 周忆昭. 四川巴中市嗜群血蜱发生特点及防制措施. *医学动物防制* 2001; (12): 623-4.
345. 鲜升文, 陈大林, 文兴诚, 谭永孝, 罗兆平, 张太秀. 四川省巴中市嗜群血蜱的调查与防治. *中国兽医科技* 2000; (10): 22-4.
346. 孙家刚, 李开均, 魏洪, 何忠武. 四川省苍溪县暴发嗜群血蜱的研究. *中国兽医科学* 2006; (09): 719-23.
347. 周勇志, 周金林, 曹杰, 龚海燕. 四种硬蜱的实验室人工饲养和部分生物学特性的观察. *中国兽医寄生虫病* 2003; (02): 23-5.
348. 鞠文东, 徐宁, 耿聪, 等. 绥芬河口岸媒介蜱携带埃立克体及无形体感染的调查. *口岸卫生控制* 2022; **27**(04): 58-62.
349. 呼满霞. 绥芬河口岸蜱类调查报告. *口岸卫生控制* 2014; **19**(02): 44-5.
350. 崔文富. 绥芬河市郊区蜱的生态调查. *中国国境卫生检疫杂志* 1989; (05): 326-7+32.
351. 李基旭, Roh JY, Park Wl, 等. 图们江流域边境地区蜱携带斑点热群立克次体调查研究. *中华预防医学杂志* 2019; **53**(11): 1130-5.
352. 李基旭, Cho S-H, 朴文, 等. 图们江流域中朝俄边境地区长角血蜱中 SFTS 病毒监测分析. *中华疾病控制杂志* 2019; **23**(02): 201-6+11-12.
353. 宋春玲, 李烨, 李琦, 等. 完达山地区蜱种调查及生态学研究. 第五届全国人畜共患病学术研讨会; 2017; 中国江苏南京; 2017. p. 152-3.
354. 陈香蕊, 崔红. 我国斑点热病原学研究进展. *中国人兽共患病杂志* 1999; (06): 81-4.
355. 张楠. 我国北方部分地区蜱传无形体病的分子流行病学调查. 吉林农业大学; 2021.
356. 王亚伟. 我国北方地区家畜和媒介蜱携带新型无形体的调查. 中国人民解放军军事医学科学院; 2016.
357. 高东旗, 曹务春, 赵秋敏, 张习坦, 方立群. 我国北方蜱中人粒细胞埃立克体 16SrRNA 基因的检测. *寄生虫与医学昆虫学报* 2000; (02): 103-8.
358. 高燕. 我国伯氏疏螺旋体分离株基因型及致病性研究. 中国人民解放军军事医学科学院; 2006.
359. 李洁, 王倩, 夏珞媛, 潘育生, 贾娜, 曹务春. 我国不同地域蜱类自然感染MIV-2 病毒的调查. *寄生虫与医学昆虫学报* 2021; **28**(02): 99-103.

360. 王宏伟, 何静, 褚宸一, 等. 我国部分地区蜱中莱姆病螺旋体的检测与基因分型研究. *中国病原生物学杂志* 2006; (02): 81-5.
361. 卢志宇. 我国部分地区硬蜱携带立克次体的分子鉴定. 军事科学院; 2021.
362. 韩华. 我国部分自然风景区蜱媒传染病病原体初步研究. 中国人民解放军军事医学科学院; 2015.
363. 王迪, 姬洪卫, 王泽东, 等. 我国东北部分地区蜱携带森林脑炎病毒的分离鉴定. *中国兽医学报* 2021; **41**(03): 469-74.
364. 王迪. 我国东北地区蜱传脑炎病毒（TBEV）流行病学调查与分析. 吉林农业大学; 2020.
365. 张志强, 魏安明, 吴益民, 胡玲美, 李红兵. 我国东北地区蜱中检出蜱传埃立克体 DNA. *微生物学杂志* 2005; (06): 63-5.
366. 刘国平, 任清明, 贺顺喜, 王峰, 杨军. 我国东北三省蜱类的分布及医学重要性. *中华卫生杀虫药械* 2008; (01): 39-42.
367. 张园. 我国黑龙江林区蜱中巴贝西虫感染调查与分离鉴定. 中南大学; 2013.
368. 张哲夫, 窦桂兰. 我国莱姆病螺旋体生物媒介的初步调查. *中华流行病学杂志* 1992; **013**(5): 271-4.
369. 王宏伟. 我国蜱中莱姆病螺旋体的分离、检测和基因分型研究. 中国人民解放军军事医学科学院; 2005.
370. 孙毅, 许荣满, 张泮河, 郭天宇, 曹务春. 我国四种常见蜱感染和经期传播莱姆病螺旋体的实验研究. 中国昆虫学会 2001 年学术年会; 2001; 中国四川成都; 2001. p. 443-5.
371. 张芳, 刘增加. 我国西北部分地区 Q 热分子流行病学调查. *中国病原生物学杂志* 2011; **6**(03): 183-5+235.
372. 贾蕾, 张芳, 刘增加. 我国西北地区蜱土拉弗菌感染的检测与基因分型研究. *中国病原生物学杂志* 2011; **6**(03): 179-82.
373. 孙毅, 许荣满, 张泮河, 郭天宇, 曹务春. 我国一些常见蜱种莱姆病螺旋体的分离与鉴定. *寄生虫与医学昆虫学报* 2002; (02): 114-9.
374. 江瑞若. 新巴贝西虫病的发现及其病原体鉴定. 中国人民解放军军事医学科学院; 2016.
375. 潘育生. 新发蜱传洱海立克次体的分离鉴定及现场流行病学调查研究. 军事科学院; 2021.
376. 张明珠. 新发山丘立克次体的流行病学、生物学和基因组学特征分析. 军事科学院; 2022.
377. 庞道毛, 艾承绪, 陈国仕. 新疆北部蜱类的区系及垂直分布. *四川动物* 1985; (03): 15-8.
378. 张璘. 新疆北疆地区蜱种分布及蜱源性病原检测方法的建立. 石河子大学; 2014.
379. 于心, 叶瑞玉, 张自建, 陈欣如. 新疆博尔塔拉和伊犁地区蜱类区系考察. *地方病通报* 1993; (02): 39-43.
380. 张桂林, 郑重, 孙响, 刘晓明, 刘然, 李海龙. 新疆地区常见蜱种生态景观分布调查. *中国媒介生物学及控制杂志* 2016; **27**(05): 432-5.
381. 廖秋萍, 王时伟. 新疆喀什市周边地区绵羊寄生蜱种类调查. *塔里木大学学报* 2005; (04): 42-3.
382. 贵有军, 史深, 罗勇军, 等. 新疆蜱传疾病及蜱媒防制. *中国动物传染病学报*: 1-8.
383. 陈秋语, 罗金, 陈泽, 等. 新疆伊犁哈萨克自治州蜱及牛羊蜱传病分子流行病学调查. *中国动物传染病学报* 2016; **24**(03): 72-7.
384. 陈秋语. 新疆伊犁州动物蜱传疾病分子流行病学调查. 中国农业科学院; 2016.
385. 张桂林, 孙响, 刘然, 郑重, 刘晓明, 伊小平. 新疆中哈边境地区分离到远东型和西伯利亚型蜱传脑炎病毒. *中国人兽共患病学报* 2017; **33**(04): 312-5.
386. 付维明, 焦丹, 丁淑丽, 等. 逊克县边疆俄罗斯民族村蜱携带斑点热群立克次体的调查. *口岸卫生控制* 2021; **26**(04): 60-3.
387. 杨军, 王艳梅, 鞠文东, 等. 逊克县火山熔岩地区蜱携带新发蜱传病原体及复合感染调查. *中国媒介生物学及控制杂志* 2016; **27**(04): 341-4.
388. 闵鹏飞. 延边地区蜱虫种类及蜱传疾病分子流行病学调查. 延边大学; 2022.
389. 贺晓燕, 李志清, 张军民. 野兔寄生蜱自然感染土拉弗氏菌的初步研究. *中华卫生杀虫药械* 2014; **20**(04): 376-8+81.

390. 高远. 一种蜱源新病毒的发现、分离鉴定及检测方法建立与初步流行调查. 东北农业大学; 2021.
391. 高东旗, 曹务春, 张习坦, 赵秋敏, 朱建华, 方立群. 应用半巢式 PCR 检测我国北方一些蜱种中的查菲埃立克体. *中国媒介生物学及控制杂志* 2000; (03): 220-4.
392. 王春生, 万康林, 郭建华, 等. 应用直接免疫荧光抗体法(DFA)对媒介蜱、动物伯氏疏螺旋体的检测. *中国卫生检验杂志* 2008; (03): 495-6.
393. 谷存国, 李雅江, 潘宝山. 应用 ELISA 技术检测黑河林区人群莱姆病螺旋体抗体. *黑龙江医药科学* 2003; (01): 63.
394. 毕德增, 张健之, 范明远. 用鸡胚卵黄囊分离斑点热群立克次体的研究. *中国媒介生物学及控制杂志* 1997; (01): 47-9.
395. 陈敏, 范明远, 徐国民, 等. 用 PCR/RFLP 技术对黑龙江沿岸蜱类和鼠类中北亚蜱传斑点热的检测. *中华流行病学杂志* 1997; (01): 5-7.
396. 王倩. 云南蜱病毒的发现及其复合感染的流行病学调查研究. 山东大学; 2021.
397. 胡玲美, 魏安明, 吴益民, 张志强, 李红兵, 鲁志新. 长白山地区几种蜱携带埃立克体的调查. *中国公共卫生* 2004; (03): 96-7.
398. 王卓, 吴琼, 杨慎江, 等. 长白山地区蜱类和鼠类中查菲埃立克体感染调查及 16S rRNA 序列分析. *中国病原生物学杂志* 2017; **12**(10): 971-4+8.
399. 王春生, 杨修军, 王艳华, 全毓文, 刘福才, 柳跃远. 长白山莱姆病自然疫源地及宿主感染状况分析. *职业与健康* 2001; (04): 87.
400. 孙毅, 许荣满, 张泮河, 郭天宇, 曹务春. 长角血蜱、嗜群血蜱经期传播莱姆病螺旋体 Borrelia garinii 的实验研究. *中国人兽共患病杂志* 2003; (01): 37-41.
401. 姜理平, 莫世华, 程苏云, 孟真, 罗芸, 叶菊莲. 浙江省蜱媒疾病检验方法建立应用. *现代实用医学* 2006; (11): 847-8.
402. 关浩. 中朝边境地区蜱种及蜱传立克次体的调查研究. 沈阳农业大学; 2019.
403. 洪晓坤, 刘阳, 徐宝梁, 等. 中朝边境长白口岸蜱类携带病原体情况研究. *口岸卫生控制* 2016; **21**(04): 57-9.
404. 王卓, 吴琼, 黄冠鹏, 等. 中朝中俄边境地区蜱携带土拉弗菌的调查. *中华卫生杀虫药械* 2022; **28**(05): 447-50.
405. 华满堂, 金兆清, 林涛, 宫占威, 何成, 刘长林. 中俄、中哈边境地区莱姆病自然疫源地调查研究. *解放军预防医学杂志* 1999; (06): 402-5.
406. 单林, 李滨, 任丽. 中俄边境口岸莱姆病调查分析报告. *中国公共卫生管理* 2006; (04): 340-1.
407. 冯盼盼. 中俄部分边境地区蜱媒病毒调查. 中国疾病预防控制中心; 2021.
408. 梁慧杰, 郭天宇, 黄洋. 中国 20 个边境口岸蜱类调查. *中华卫生杀虫药械* 2019; **25**(05): 478-80.
409. 高旭婷. 中国东北地区蜱虫携带新型圆环病毒的分子检测. 内蒙古民族大学; 2018.
410. 杨军, 徐文兴, 白阳森, 刘国平. 中国逊克和俄罗斯波亚尔科沃边境蜱类监测分析. *中华卫生杀虫药械* 2013; **19**(04): 329-31.
411. 程成, 王艳梅, 翟琪安, 等. DNA 条形码技术在黑瞎子岛蜱类鉴别中的应用. *中国公共卫生管理* 2020; **36**(05): 675-8.
412. 王艳梅, 程成, 鞠文东, 等. DNA 条形码技术在鉴定绥芬河口岸日本血蜱和嗜群血蜱中的应用. *口岸卫生控制* 2019; **24**(03): 51-4.
413. 陈岩, 陈雷. PCR/SSCP 技术检测蜱体内斑点热群立克次体. *中国卫生检验杂志* 2000; (03): 350-1.
414. 魏安明, 吴益民, 胡玲美, 张志强, 冯立, 刘华. PCR 检测东北地区部分蜱种中埃立克体 DNA. *解放军预防医学杂志* 2001; (02): 149.
