# Supplementary material for: An integrated data analysis reveals distribution, hosts, and pathogen diversity of Haemaphysalis concinna
Source: Parasit Vectors. 2024 Feb 27;17:92. doi: 10.1186/s13071-024-06152-5 (PMC10900579; doi:10.1186/s13071-024-06152-5)
Supplement: Supplementary file 2 — Additional file 2: Table S1. Environmental and meteorological variables downloaded for ecological modeling for Haemaphysalis concinna. [file 13071_2024_6152_MOESM2_ESM.pdf]

Table S1: Environmental and meteorological variables downloaded for ecological modeling for *Haemaphysalis concinna*

Variable	Description	Source
BIO1	Annual mean temperature (°C)	WorldClim database
BIO2	Mean diurnal range (Mean of monthly (max temp-min temp)) (°C)	
BIO3	Isothermality (BIO2/BIO7)(*100)	
BIO4	Temperature seasonality (standard deviation*100)	
BIO5	Max temperature of warmest month (°C)	
BIO6	Min temperature of coldest month (°C)	
BIO7	Annual range of temperature (BIO5- BIO6) (°C)	
BIO8	Mean temperature of wettest quarter (°C)	
BIO9	Mean temperature of driest quarter (°C)	
BIO10	Mean temperature of warmest quarter (°C)	
BIO11	Mean temperature of coldest quarter (°C)	
BIO12	Annual precipitation (mm)	
BIO13	Precipitation of wettest month (mm)	
BIO14	Precipitation of driest month (mm)	
BIO15	Precipitation seasonality(Coefficient of variation)	
BIO16	Precipitation of wettest quarter (mm)	
BIO17	Precipitation of driest quarter (mm)	
BIO18	Precipitation of warmest quarter (mm)	
BIO19	Precipitation of coldest quarter (mm)	
Elevation	Elevation	
Slope	Slope	
Aspect	Aspect	
Percent tree cover	Percent tree cover	Geospatial Information Authority of Japan
Land cover	Land cover	

Legend for Land Cover type

Code	Class Name
1	Broadleaf Evergreen Forest
2	Broadleaf Deciduous Forest
3	Needleleaf Evergreen Forest
4	Needleleaf Deciduous Forest
5	Mixed Forest
6	Tree Open
7	Shrub
8	Herbaceous
9	Herbaceous with Sparse Tree/Shrub
10	Sparse vegetation
11	Cropland
12	Paddy field
13	Cropland/Other Vegetation Mosaic
14	Mangrove
15	Wetland
16	Bare area, consolidated (gravel, rock)
17	Bare area, unconsolidated (sand)
18	Urban
19	Snow/Ice
20	Water bodies
