# Supplementary material for: An integrated data analysis reveals distribution, hosts, and pathogen diversity of Haemaphysalis concinna
Source: Parasit Vectors. 2024 Feb 27;17:92. doi: 10.1186/s13071-024-06152-5 (PMC10900579; doi:10.1186/s13071-024-06152-5)
Supplement: Supplementary file 3 — Additional file 3: Table S2. Known occurrence locations of Haemaphysalis concinna used for ecological niche modelling. [file 13071_2024_6152_MOESM3_ESM.pdf]

Table S2: Known occurrence locations of *Haemaphysalis concinna* used for ecological niche modeling*

Country	Region	Longitude	Latitude
Austria	Burgenland	16.613889	47.917222
		16.316667	47.066667
		16.848889	47.864167
		16.878617	47.747217
	Lower Austria	16.702500	48.115000
		16.830278	48.298889
		15.703018	48.490251
		16.846942	48.691283
		16.280000	48.130000
		16.291740	48.287610
		16.316667	48.300000
	Vienna	16.400000	48.200000
		16.326250	48.277030
		16.442400	48.194260
		16.483611	48.195278
Croatia	Bjelovar-Bilogora	16.878500	45.786100
	Brod-Posavina	18.297222	45.188611
		18.394444	45.155833
	County of Osijek-Baranja	18.773889	45.663333
		18.024444	45.473333
		18.399167	45.254444
		18.472222	45.403889
		18.611667	45.740833
		18.214400	45.332100
		18.708333	45.564722
		18.283056	45.545556
	County of Poževsko-Slavonska	17.662500	45.455556
		17.537100	45.426400
	County of Virovitica-Podravina	17.864722	45.515278
	County of Zagreb	16.336400	45.744100
	Vukovar-Sirmium	19.092222	45.232500
Democratic People's Republic of Korea	Ryanggang Province	128.166667	41.350000
France	Centre-Val de Loire	2.522820	46.708300
		2.150420	47.203400
		2.206690	47.225110
		2.207620	47.225280
		2.217430	47.246500
		2.231200	47.266630
		2.227550	47.269390
		2.273640	47.273860

France	Centre-Val de Loire	2.117840	47.276500
		2.282750	47.322800
		2.283810	47.323290
		2.227170	47.345990
		2.362920	47.378430
		1.002140	46.672400
		1.046500	46.684260
		1.635450	47.767220
		2.420770	47.831380
		2.339360	47.883140
		2.343620	47.886290
		2.248430	48.062820
		2.604330	48.071460
		2.070000	47.460000
		1.474210	47.316800
		1.367490	47.322230
		1.452630	47.326990
		1.958670	47.378700
		1.553840	47.403100
		1.337180	47.408250
		1.743170	47.411440
		1.256440	47.479660
		1.257050	47.479860
		1.257180	47.479920
		1.865170	47.557290
		1.331300	47.558860
		0.662340	47.749160
		1.304080	47.887080
Germany	Brandenburg	13.372477	52.699025
		14.566040	52.258650
	Lower Saxony	10.824600	53.112800
		8.252500	52.943500
		9.260000	53.300000
		9.261667	53.304444
	Mecklenburg-Vorpommern	14.034056	53.643406
		14.043847	53.733800
	Saxony	12.959484	51.263582
		12.734131	51.552537
		12.674038	51.627761
Hungary	Bekes County	20.933333	47.083333
	Borsod-Abauj-Zemplen	20.738500	48.293400
		20.856000	47.805000

Hungary	Fejér	18.358000	47.395000
	Gyor-Moson-Sopron	17.400000	47.950000
	Pest megye	19.210100	47.296700
	Tolna megye	18.766667	46.116667
	Zala	17.242700	46.708800
Iran	Lorestan Province	48.240000	33.850000
Italy	Basilicata	16.154167	40.544444
	Emilia-Romagna	12.250000	44.866667
Japan	Hokkaido	145.100000	43.670000
		145.089996	43.639999
Lithuania	Kaunas	22.924028	55.464083
Poland	Greater Poland	16.114098	52.148505
	Lower Silesian	17.213433	51.411425
		16.643100	51.205400
		16.695100	51.221100
		16.711900	51.228400
		16.766939	51.239125
		17.063428	51.509058
Romania	Alba	23.562736	45.948229
	Tulcea	28.939243	44.695848
Russia	Altai	86.057400	51.884760
	Altai Krai	85.978280	52.148210
		85.915660	52.312870
	Amur Oblast	127.700000	50.666667
		129.950000	49.616667
		126.133333	53.366667
		128.366667	51.400000
		129.883333	52.333333
		127.400000	52.316667
		128.333333	51.883333
	Irkutsk Oblast	104.783333	52.416667
	Jewish Autonomous Oblast	132.800000	48.683333
		132.650000	48.066667
		131.983333	49.016667
	Jewish Autonomous Oblast	131.050000	47.916667
	Kamchatka Krai	158.730000	53.030000
	Khabarovsk	134.333333	47.133333
		135.000000	48.000000
		134.816667	47.983333
		135.016667	48.250000
		135.533333	48.600000
		135.933333	48.850000

Russia	Khabarovsk	135.000000	48.250000
		136.900000	50.583333
		136.866667	50.400000
		136.966667	50.433333
		136.600000	49.366667
		135.083611	48.482500
	Kirov Oblast	50.000000	58.000000
	Novosibirsk Oblast	82.900000	55.000000
	Primorye	131.966667	45.270833
	Zabaykalsky Krai	120.759702	52.184681
	Kemerovo Oblast	87.950000	55.883333
		87.600000	53.700000
		85.583333	55.033333
	Tomsk Oblast	84.983333	56.483333
Slovakia	Košický kraj	20.916667	48.616667
		20.779050	48.581650
	Trnava	17.816667	48.250000
		17.583333	47.900000
South Korea	Jeju	126.533000	33.367000
Spain	Asturias	-5.173370	43.440600
		-5.870000	43.310000
		-5.242222	43.480000
		-5.161390	43.426000
		-5.229830	43.426100
The Czech Republic	South Moravian	16.966028	48.642203
		16.969444	48.716667
		16.490278	48.851944
		16.933333	48.666667
		16.783333	48.750000
		16.806389	48.818889
		15.996956	48.818508
Turkey	Samsun	36.083333	41.633333
China	Fujian	117.999884	27.745091
	Heilongjiang	130.773333	43.886667
		129.041389	43.947778
		129.035278	43.953611
		130.738633	44.355000
		131.150000	44.383333
		130.570000	44.520000
		127.483614	44.759365
		129.693662	44.773595
		129.500000	44.916667

China	Heilongjiang	129.186111	44.975556
		129.187083	44.976667
		129.450000	45.030694
		129.458611	45.035278
		128.733333	45.516667
		126.964357	45.554275
		127.416667	45.733333
		128.816667	45.833333
		133.150000	45.846667
		128.016667	45.933333
		130.916667	46.383333
		133.200000	46.716667
		133.985000	46.823333
		128.383333	47.400000
		130.253333	47.575000
		129.194722	47.686389
		131.471667	47.706667
		133.483333	47.716667
		128.870000	47.820278
		132.852222	47.870833
		130.656944	48.013889
		134.336667	48.300000
		129.936389	48.665000
		130.058333	48.873333
		128.470000	49.570000
		127.438333	50.235000
		124.270000	50.576667
		124.275278	50.645833
		124.219435	52.322473
	Liaoning	123.435598	41.841465
	Jilin	128.124888	42.428170
		130.483800	42.652459
		129.500000	42.900000
		129.509995	42.900002
		127.973988	43.164721
		128.029023	43.182640
		129.821269	43.299591
		129.237709	43.317402
		129.765416	43.324037
		128.231100	43.372780
		129.940763	43.530406
		127.957286	43.939706

China	Jilin	128.601389	43.951250
		127.703194	43.962139
		125.894583	44.126944
		126.050000	44.983333
		125.949799	41.951398
	Inner Mongolia	118.464768	41.416567
		119.742465	49.218216
		123.537728	49.430144
		117.548910	49.533591
		121.383333	49.566667
		121.616314	50.026567
		119.374214	50.210000
		119.604157	50.234194
		120.186634	50.248132
		119.887458	50.336617
		120.824648	50.374854
		119.397449	50.628733
		120.072904	50.746316
		121.498897	50.760825
		120.172581	50.787206
		120.792440	51.261409
		120.611714	51.277176
		119.901328	51.341450
		120.330713	51.365404
		120.163558	51.512158
		120.131014	51.515333
		120.772617	52.195917
	Shaanxi	107.150000	32.600000
		106.866667	34.750000
		108.850000	36.050000
	Sichuan	105.960000	31.750000
		102.693333	32.918056
	Xinjiang Uygur Autonomous Region	80.900000	44.616667
		82.983811	46.750703
		86.716667	48.491667

*Geographic locations listed here were all actual known locations with exact longitude and latitude collected from field survey, reference book, literature search , Global Biodiversity Information Facility and GenBank.
