# Supplementary material for: An integrated data analysis reveals distribution, hosts, and pathogen diversity of Haemaphysalis concinna
Source: Parasit Vectors. 2024 Feb 27;17:92. doi: 10.1186/s13071-024-06152-5 (PMC10900579; doi:10.1186/s13071-024-06152-5)
Supplement: Supplementary file 4 — Additional file 4: Text S2. Basic characteristics of data collection. [file 13071_2024_6152_MOESM4_ESM.pdf]

Text S2: Basic characteristics of data collection

Data sources include field surveys, reference books, literature review, Global Biodiversity Information Facility, and GenBank. In our field survey, we collected *H. concinna* from 36 locations in five provinces in China. we extracted relevant information on *H. concinna*, including host species and geographic distribution, from the reference book entitled "Fauna Sinica-Arachnida Ixodida." Through the literature review, we identified 1424 relevant studies, eliminated 311 duplicate records, and further analyzed the studies that met the inclusion and exclusion criteria after reading the full text. Finally, we included 414 articles and extracted their location information, host information, and pathogen information. From the GBIF website, we obtained 45 valid coordinate points, and from the GenBank website, we extracted geographic and pathogen information of *H. concinna.*

By sorting and cleaning the above information, we have obtained 703 unique geographic information records in 34 countries, including China (319 records), Russia (68 records), Hungary (61 records), Slovakia (43 records), France (36 records), Croatia (26 records), Austria (21 records), Czech Republic(17 records), Turkey (17 records), Germany (14 records), Iran (13 records), Poland (12 records), Serbia (ten records), Romania (seven records), Spain (seven records), Italy (six records), Japan (five records), Greece (three records), South Korea (two records), Ukraine (two records), Azerbaijan (one record), Belarus (one record), Bosnia and Herzegovina (one record), Bulgaria (one record), Cyprus (one record), North Korea (one record), Georgia (one record), Kazakhstan (one record), Kyrgyzstan (one record), Lithuania (one record), Moldova (one record), Armenia (one record), Turkmenistan (one record), Uzbekistan (one record).

Our research identified a total of 83 different microbial species present in *H. concinna*, including 40 human pathogens, 8 animal pathogens, and 35 microbes with unknown pathogenicity risks. Among them, 80 microbes can be used to conduct a meta-analysis. The remaining three species, namely Jingmen tick virus, Dabieshan tick virus, and *R. argasii*, had no detectable positive rates.

A total of 439 records of host information were obtained, including Bovidae (61 records), Turdidae (45 records), Sylviidae (37 records), Canidae (33 records), Fringillidae (30 records), Muridae (28 records), Cricetidae (21 records), Cervidae (19 records), Locustellidae (16 records), Leporidae (13 records), Paridae (13 records), Passeridae (11 records), Corvidae (nine records), Lacertidae (nine records), Mustelidae (eight records), Sciuridae (eight records), Erinaceidae (seven records), Soricidae (seven records), Hominidae (six records), Felidae (six records), Phasianidae (six records), Motacillidae (six records), Anatidae (five records), Equidae (four records), Sturnidae (four records), Laniidae (three records), Muscicapidae (three records), Phylloscopidae (three records), Sittidae (three records), Talpidae (three records), Accipitridae (two records), Certhiidae (two records), Charadriidae (one record), Colubridae (one record), Camelidae (one record), Procyonidae (one record), Scolopacidae (one record), Suidae (one record), Upupidae (one record), Viverridae (one record). From a national perspective, Hungary boasted the highest number of host animal species, with 42 different species recorded. Slovakia followed closely with 37 species, while China, Turkey, and Russia had 23, 21, and 16 recorded species respectively.
