# Supplementary material for: An integrated data analysis reveals distribution, hosts, and pathogen diversity of Haemaphysalis concinna
Source: Parasit Vectors. 2024 Feb 27;17:92. doi: 10.1186/s13071-024-06152-5 (PMC10900579; doi:10.1186/s13071-024-06152-5)

# Figure S2: The distribution of pathogens in different countries

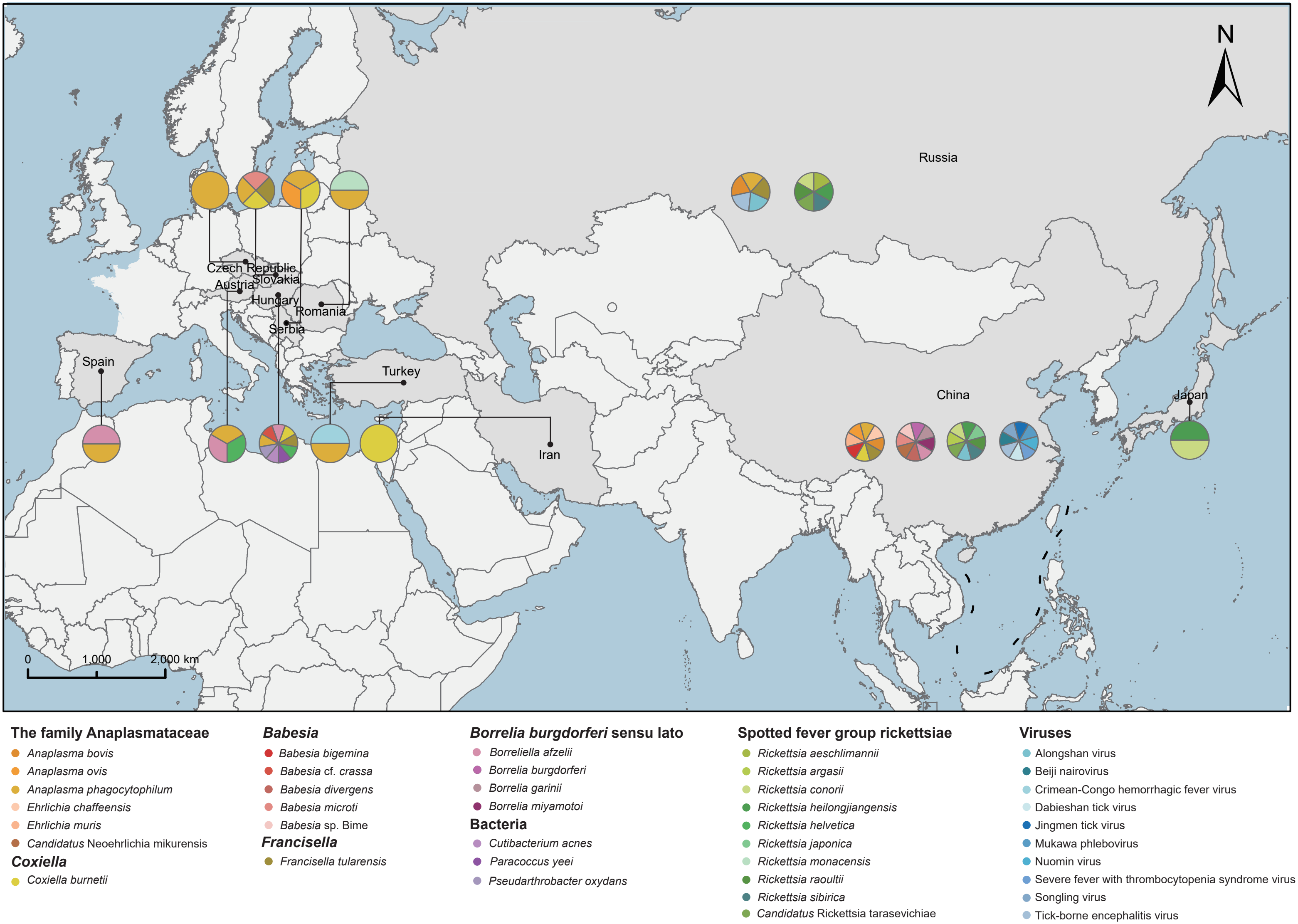

Supplement: Supplementary file 5 — Additional file 5: Figure S2. The distribution of pathogens in different countries. [file 13071_2024_6152_MOESM5_ESM.pdf]
