# Supplementary material for: An integrated data analysis reveals distribution, hosts, and pathogen diversity of Haemaphysalis concinna
Source: Parasit Vectors. 2024 Feb 27;17:92. doi: 10.1186/s13071-024-06152-5 (PMC10900579; doi:10.1186/s13071-024-06152-5)

Figure S3: Phylogenetic analysis of *Haemaphysalis concinna*-associated microbes

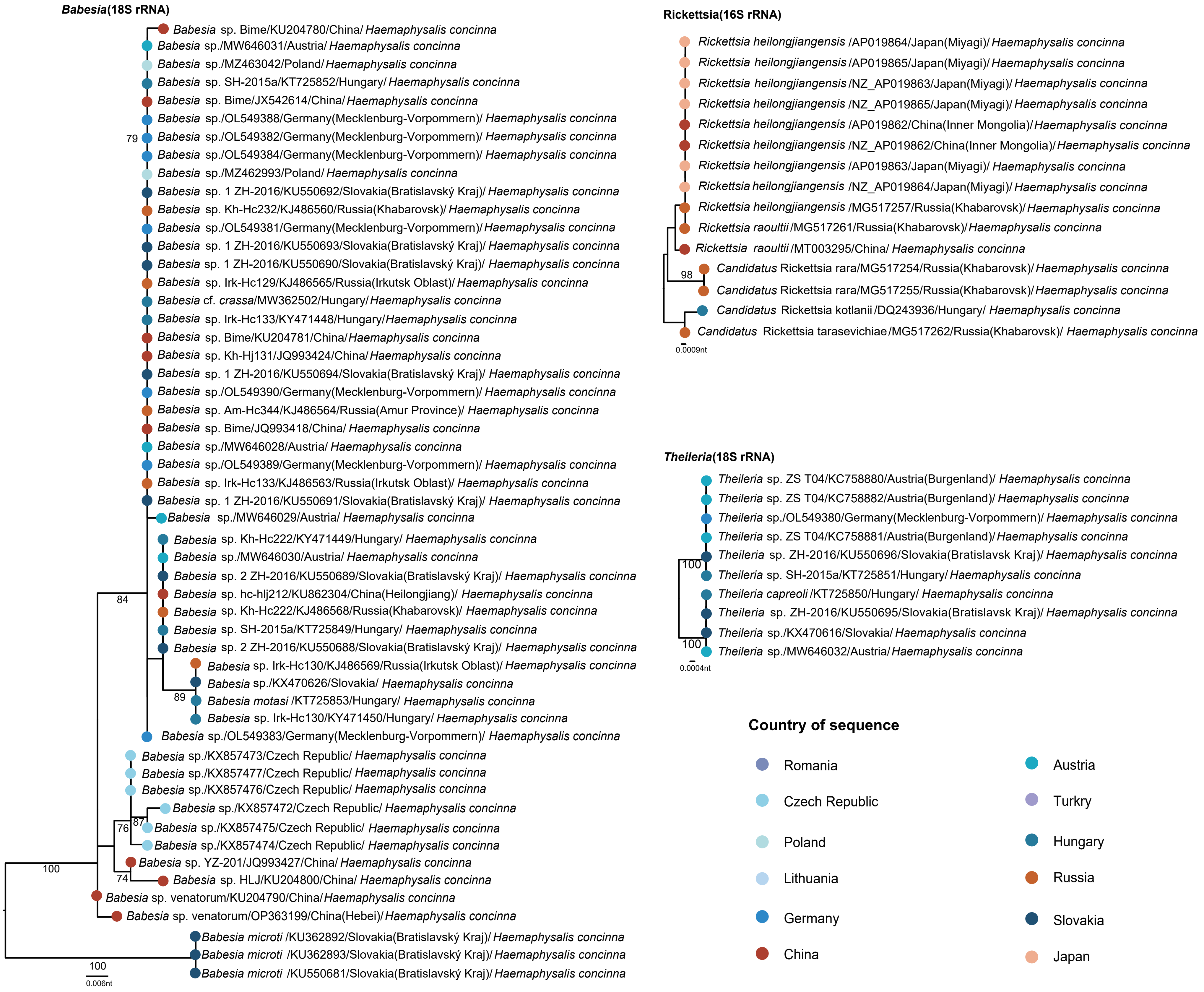

Supplement: Supplementary file 6 — Additional file 6: Figure S3. Phylogenetic analysis of Haemaphysalis concinna-associated microbes [file 13071_2024_6152_MOESM6_ESM.pdf]
