# Supplementary material for: An integrated data analysis reveals distribution, hosts, and pathogen diversity of Haemaphysalis concinna
Source: Parasit Vectors. 2024 Feb 27;17:92. doi: 10.1186/s13071-024-06152-5 (PMC10900579; doi:10.1186/s13071-024-06152-5)

Figure S4: Migration routes of birds in Europe susceptible to parasitism by *Haemaphysalis concinna*

Recorded locations of *H. concinna.* Closed circles represent the county-level regions, open circles represent the prefecture-level regions. The two segments of lines, matching the color of the bird species, delineate the activity range of the respective bird species

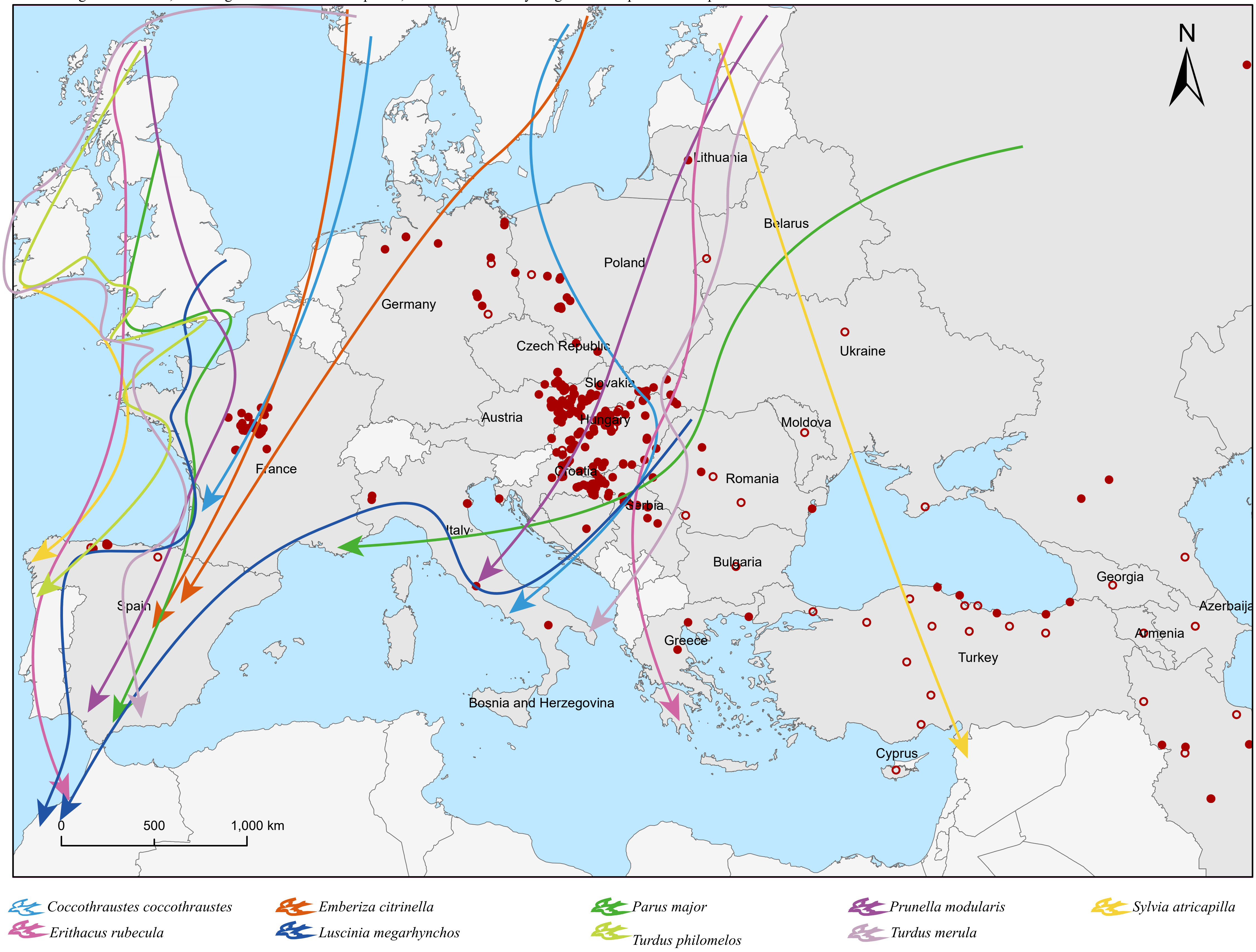

Supplementary Figure S5: Migratory birds in China primarily exhibit three migration routes

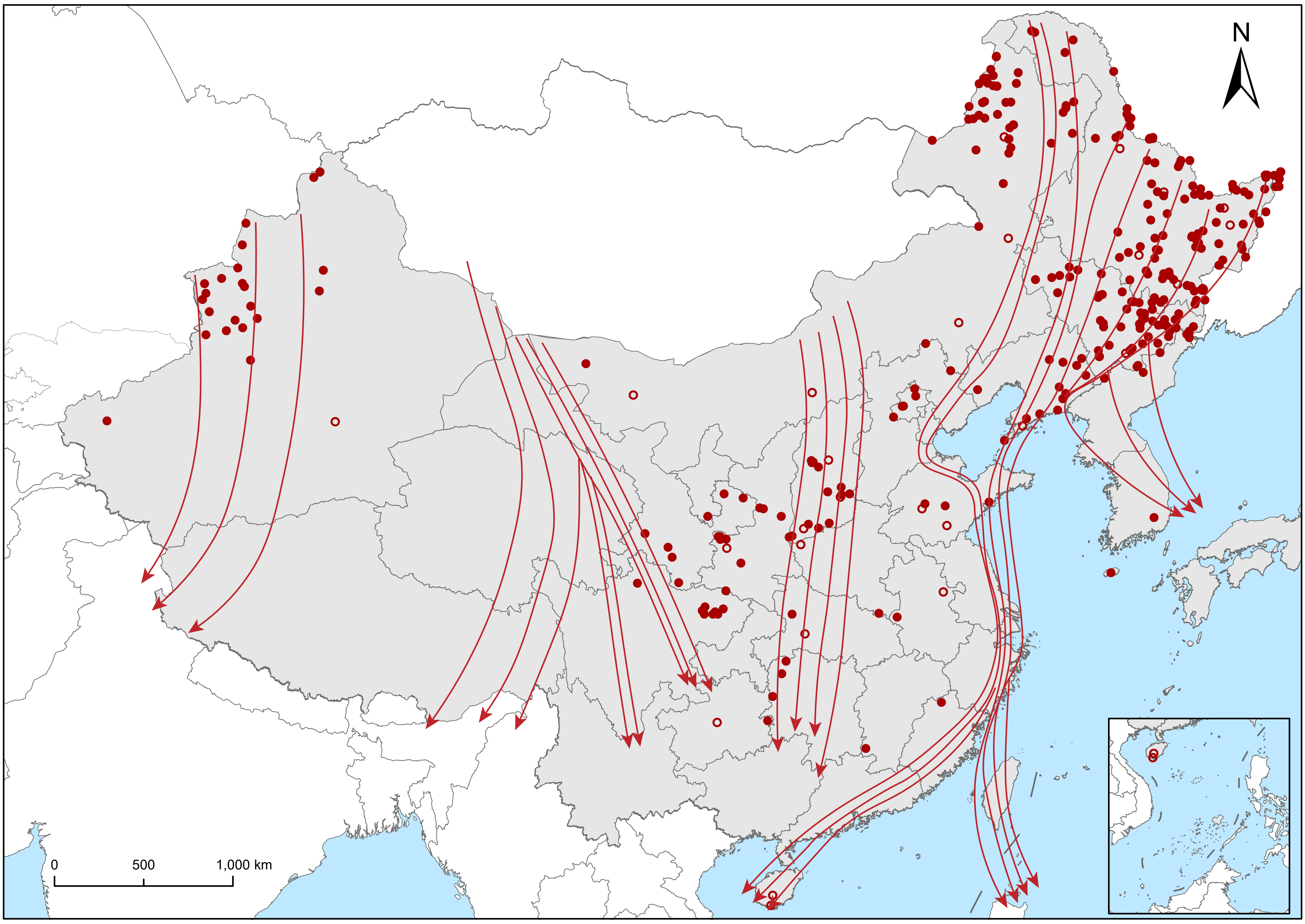

Supplement: Supplementary file 7 — Additional file 7: Figure S4. Migration routes of birds in Europe susceptible to parasitism by Haemaphysalis concinna. Figure S5. Migratory birds in China primarily exhibit three migration routes. [file 13071_2024_6152_MOESM7_ESM.pdf]
