# Supplementary material for: An integrated data analysis reveals distribution, hosts, and pathogen diversity of Haemaphysalis concinna
Source: Parasit Vectors. 2024 Feb 27;17:92. doi: 10.1186/s13071-024-06152-5 (PMC10900579; doi:10.1186/s13071-024-06152-5)
Supplement: Supplementary file 8 — Additional file 8: Figure S6. The results of Maxent model for Haemaphysalis concinna. Figure S7. Jackknife plots of Maxent model for Haemaphysalis concinna prediction. Table S3. Relative contributions of the environmental and meteorological variables to the Maxent model. Figure S8. Response curves of environmental variables to probability of Haemaphysalis concinna presence [file 13071_2024_6152_MOESM8_ESM.pdf]

Figure S6: The results of Maxent model for *Haemaphysalis concinna*

Receiver operating characteristic (ROC) curve of the Maxent model for *Haemaphysalis concinna*

The ROC curve averaged over the 25 replicate runs. The specificity is defined using predicted area

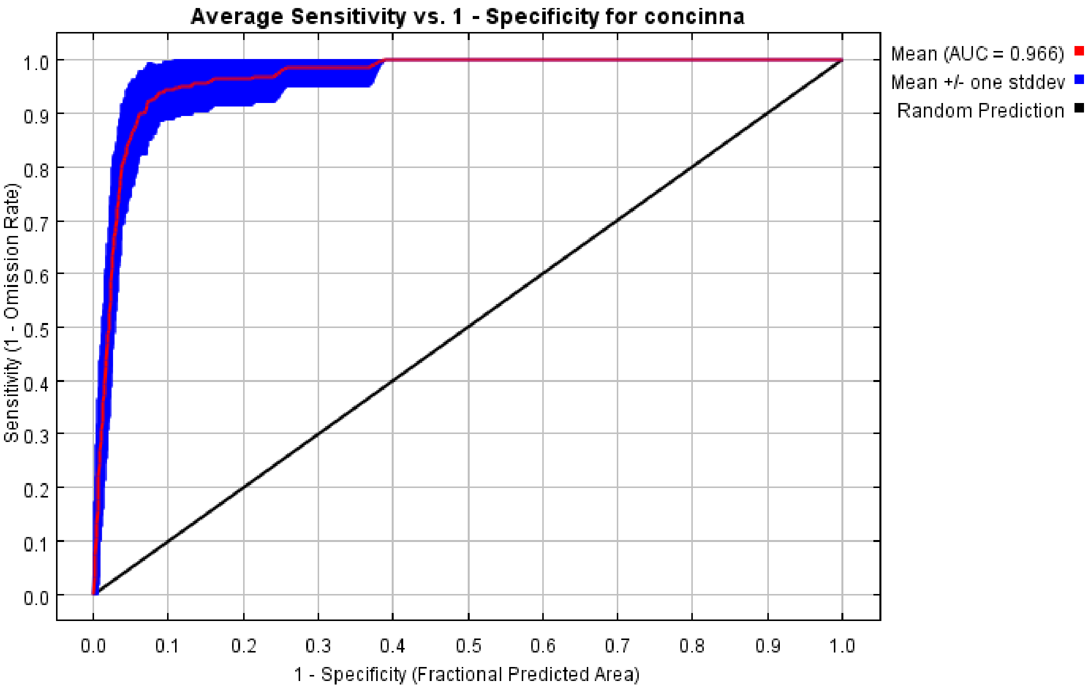

Figure S7: Jackknife plots of Maxent model for *Haemaphysalis concinna* prediction

A. Training gain plot B. test gain plot C. AUC plot

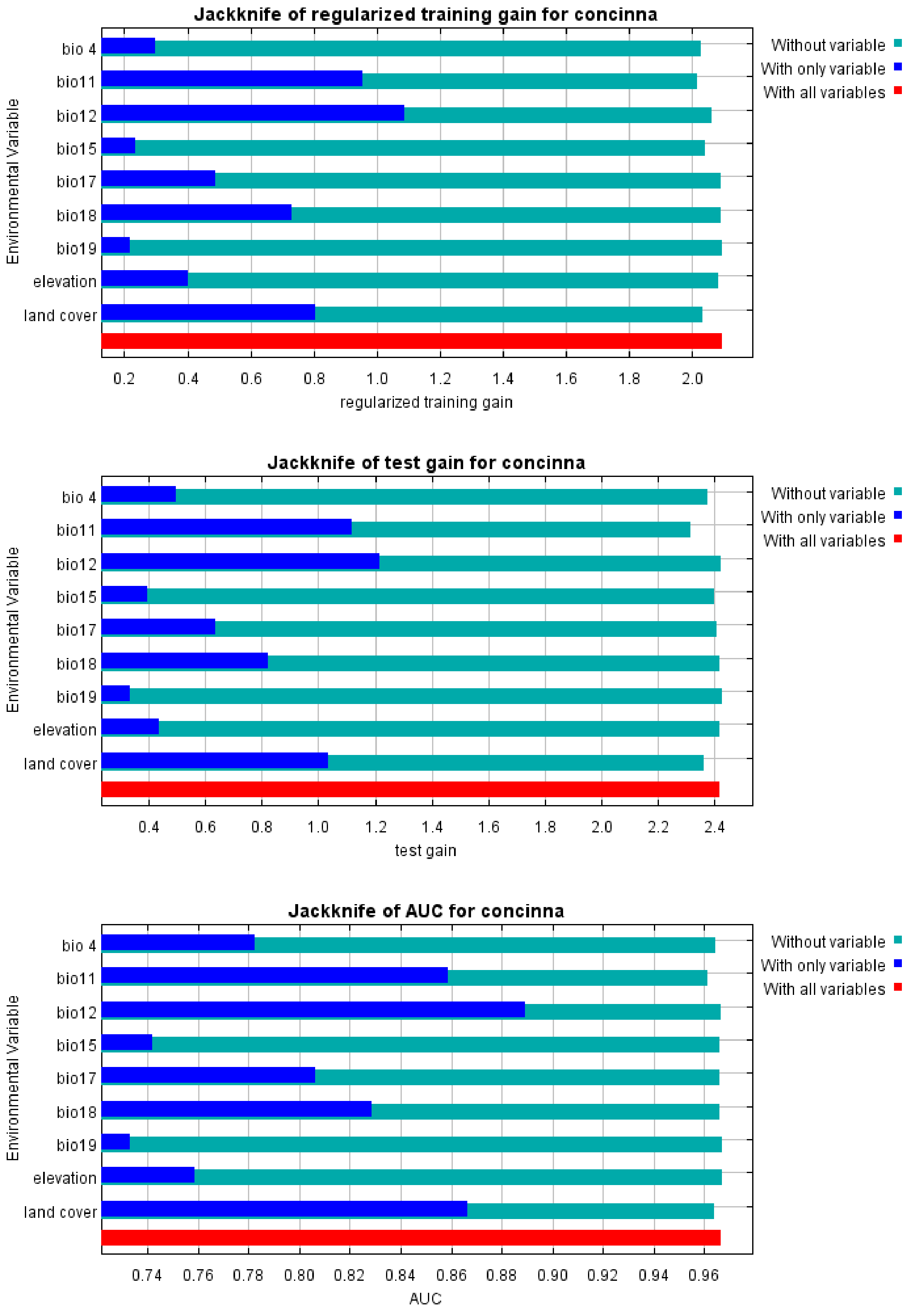

Table S3: Relative contributions of the environmental and meteorological variables to the Maxent model

Variable	Percent contribution	Permutation importance
BIO11	30.4	25.7
BIO12	26.2	19
BIO18	15.7	4
Land cover	11.5	2.9
BIO4	5.2	24.4
Elevation	5	1.9
BIO15	4.4	9.5
BIO19	1.1	4.7
BIO17	0.5	7.9

Figure S8: Response curves of environmental variables to probability of *Haemaphysalis concinna* presence

The curves show the mean response of 25 replicate MaxEnt runs (red line) and standard deviation (blue shades)

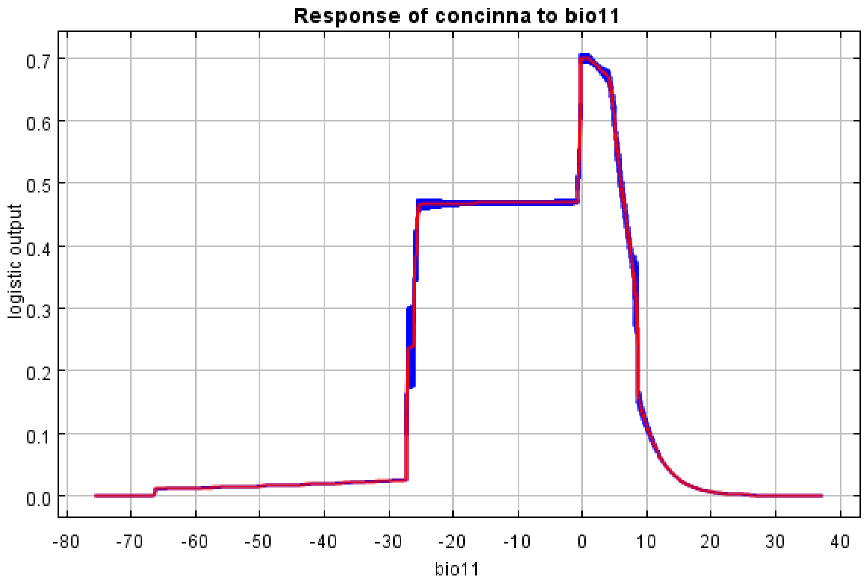

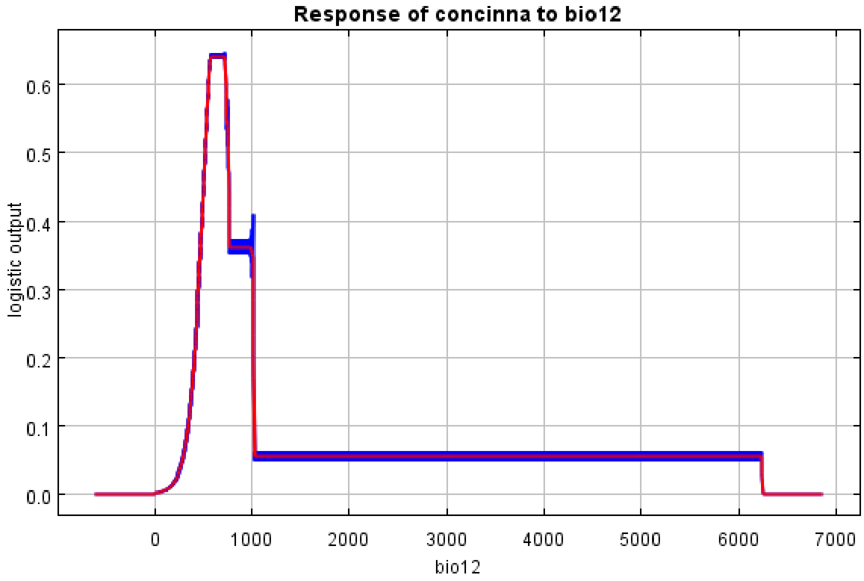

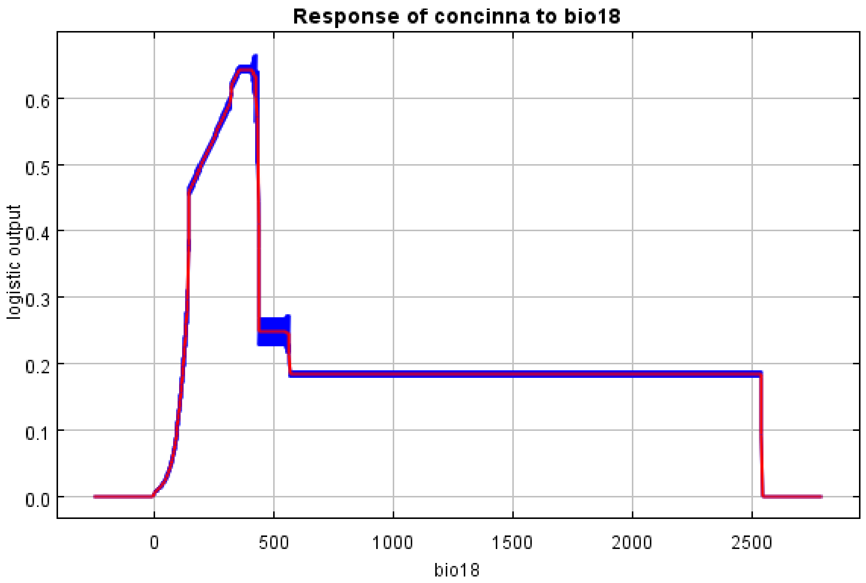

Response of concinna to bio18
logistic output
0.0
0.1
0.2
0.3
0.4
0.5
0.6
0
500
1000
1500
2000
2500
bio18

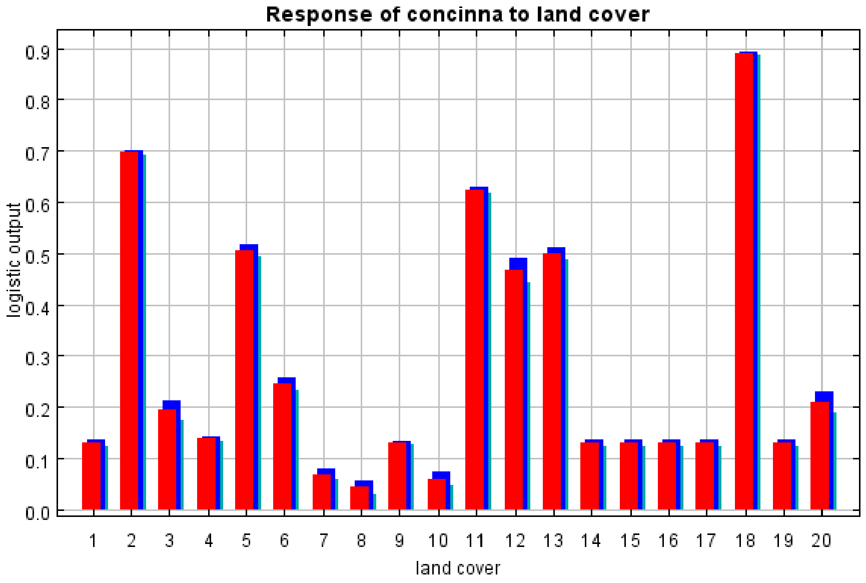

Response of concinna to land cover
logistic output
0.0
0.1
0.2
0.3
0.4
0.5
0.6
0.7
0.8
0.9
1
2
3
4
5
6
7
8
9
10
11
12
13
14
15
16
17
18
19
20
land cover

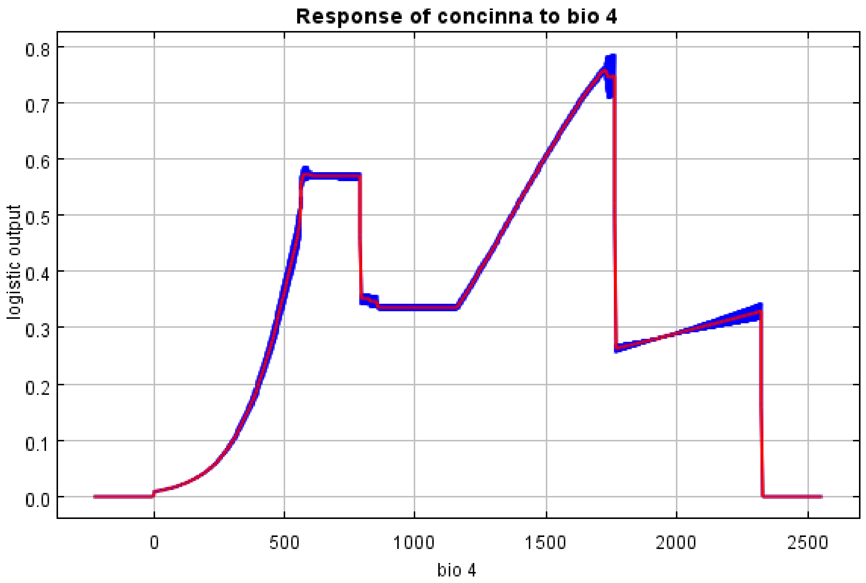
Response of concinna to bio 4
logistic output
0.0
0.1
0.2
0.3
0.4
0.5
0.6
0.7
0.8
0
500
1000
1500
2000
2500
bio 4

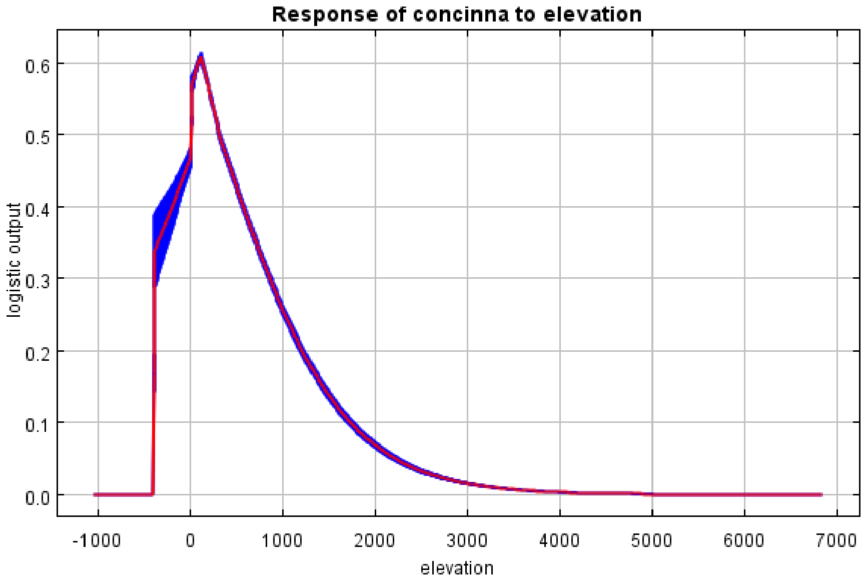
Response of concinna to elevation
logistic output
0.0
0.1
0.2
0.3
0.4
0.5
0.6
-1000
0
1000
2000
3000
4000
5000
6000
7000
elevation

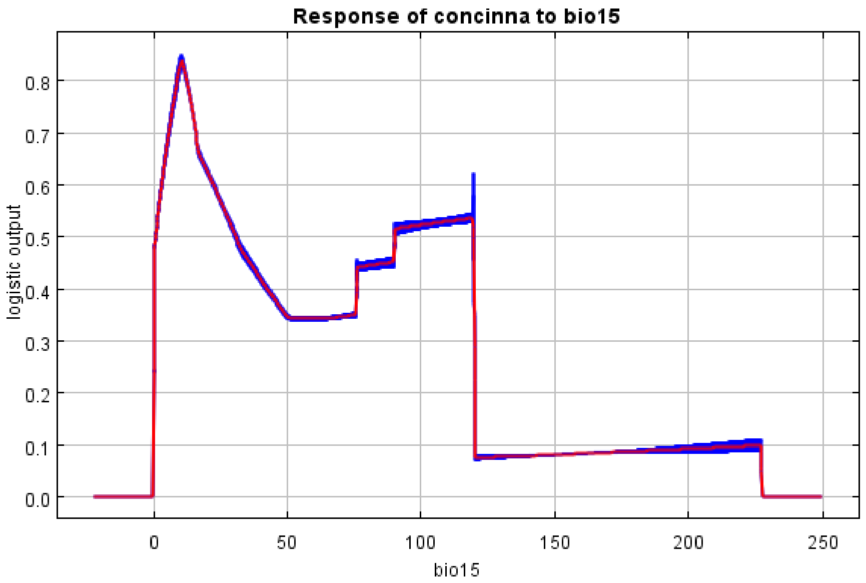
Response of concinna to bio15
logistic output
0.0
0.1
0.2
0.3
0.4
0.5
0.6
0.7
0.8
0
50
100
150
200
250
bio15

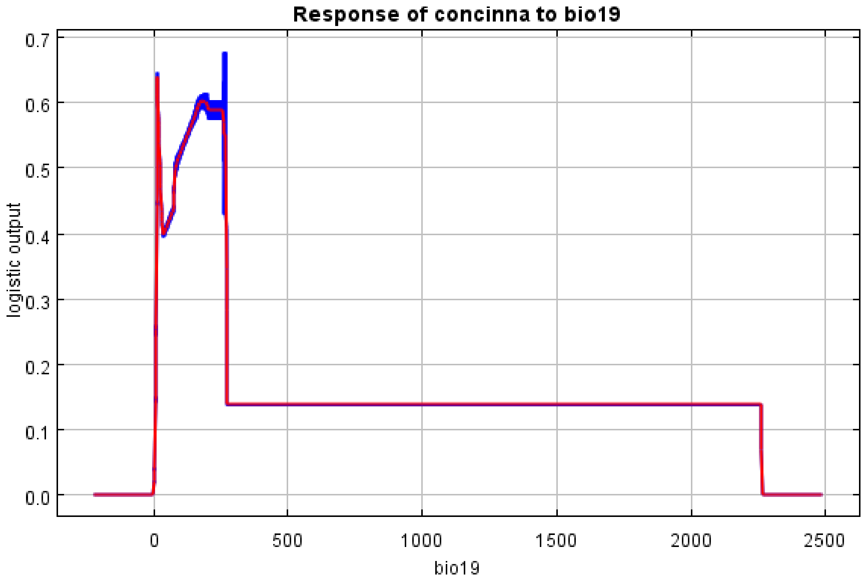
Response of concinna to bio19
logistic output
0.0
0.1
0.2
0.3
0.4
0.5
0.6
0.7
0
500
1000
1500
2000
2500
bio19

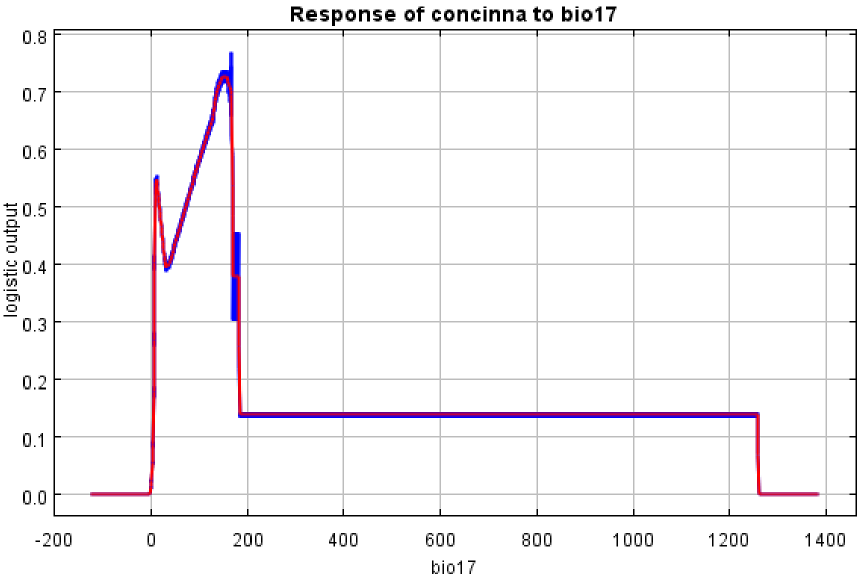
Response of concinna to bio17
logistic output
0.8
0.7
0.6
0.5
0.4
0.3
0.2
0.1
0.0
-200
0
200
400
600
800
1000
1200
1400
bio17
