# Supplementary material for: An integrated data analysis reveals distribution, hosts, and pathogen diversity of Haemaphysalis concinna
Source: Parasit Vectors. 2024 Feb 27;17:92. doi: 10.1186/s13071-024-06152-5 (PMC10900579; doi:10.1186/s13071-024-06152-5)
Supplement: Supplementary file 9 — Additional file 9: Figure S9. Prevalence of Haemaphysalis concinna-associated microbes. Figure S10. Meta-analysis of the prevalence of each Haemaphysalis concinna-associated microbes. [file 13071_2024_6152_MOESM9_ESM.pdf]

Figure S9: Prevalence of *Haemaphysalis concinna*-associated microbes

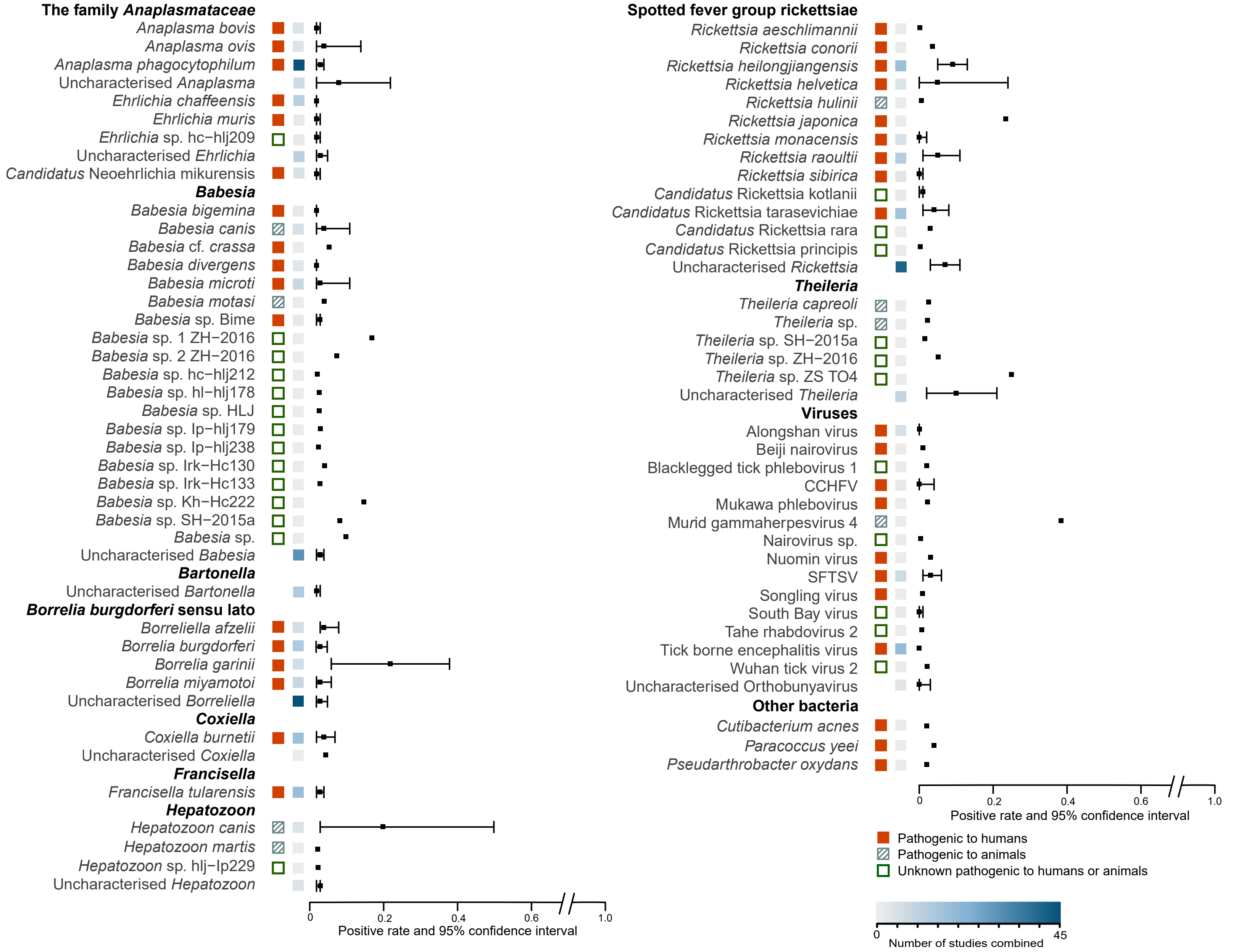

Figure S10: Meta-analysis of the prevalence of each *Haemaphysalis concinna*-associated microbes

The heterogeneity of combined studies was quantified by I^2 statistic. The fixed effect model would be applied if I^2 <50%; Otherwise, the random effect model would be applied.

Anaplasma bovis

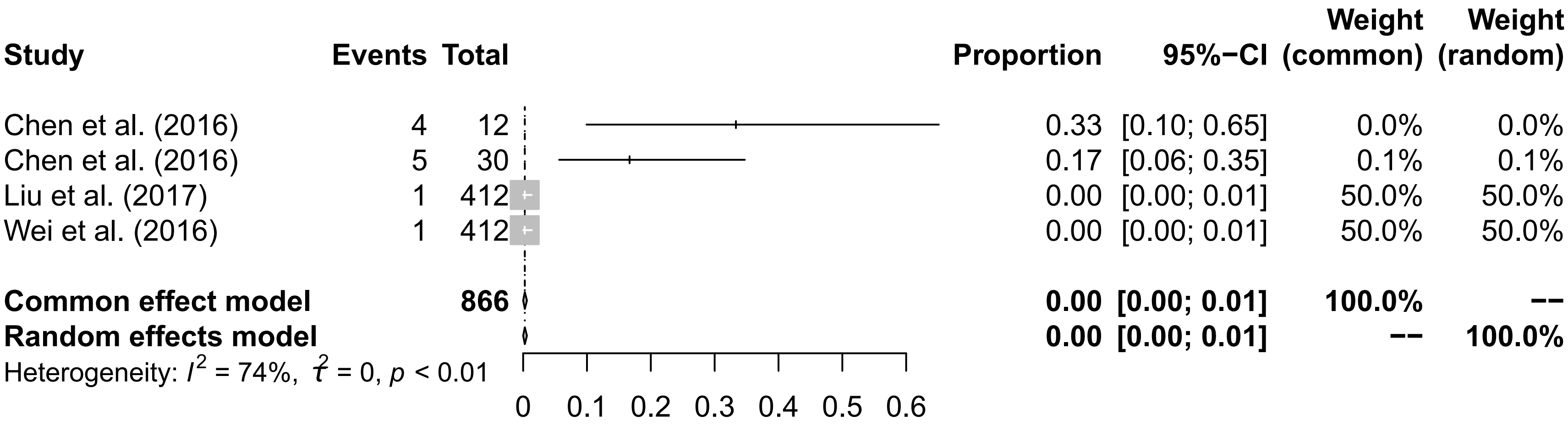

Anaplasma ovis

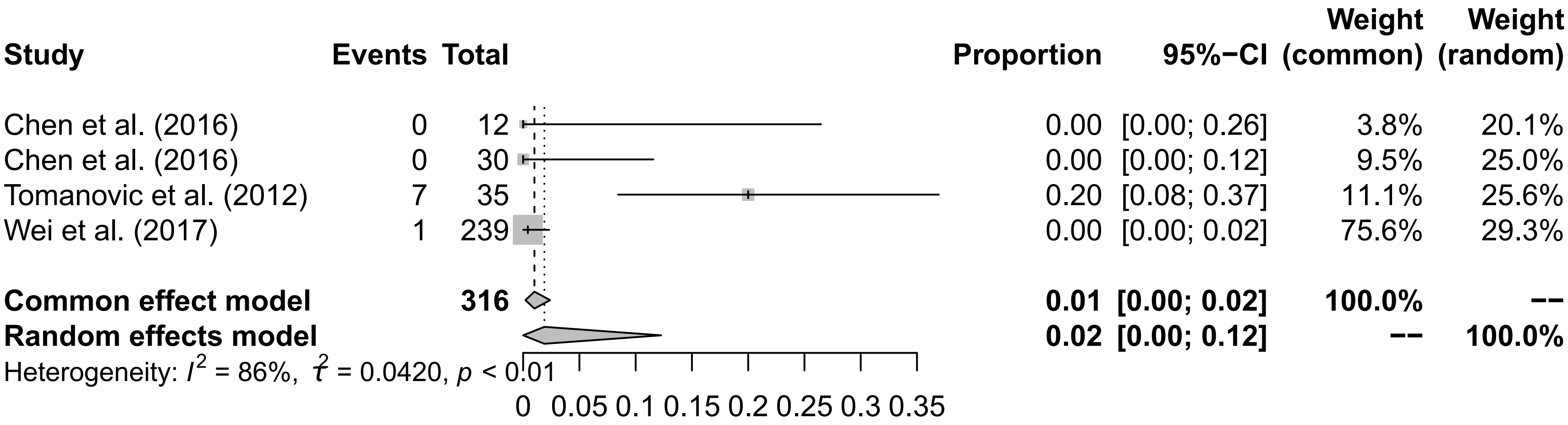

Anaplasma phagocytophilum

Study	Events	Total	Proportion	95%−CI	Weight (common)	Weight (random)
Barandika et al. (2008)	2	52	0.04	[0.00; 0.13]	0.6%	2.3%
Borsan et al. (2021)	0	6	0.00	[0.00; 0.46]	0.1%	0.7%
Cerro et al. (2022)	0	53	0.00	[0.00; 0.07]	0.6%	2.3%
Chen et al. (2016)	0	12	0.00	[0.00; 0.26]	0.1%	1.1%
Chen et al. (2016)	0	30	0.00	[0.00; 0.12]	0.3%	1.9%
Cheng et al. (2019)	5	318	0.02	[0.01; 0.04]	3.6%	3.2%
Cheng et al. (2017)	5	308	0.02	[0.01; 0.04]	3.5%	3.1%
Cheng et al. (2015)	1	49	0.02	[0.00; 0.11]	0.6%	2.3%
Cheng et al. (2015)	0	34	0.00	[0.00; 0.10]	0.4%	2.0%
Fu et al. (2010)	12	713	0.02	[0.01; 0.03]	8.0%	3.3%
Fu et al. (2015)	0	38	0.00	[0.00; 0.09]	0.4%	2.1%
Gao et al. (2000)	1	50	0.02	[0.00; 0.11]	0.6%	2.3%
Gao et al. (2001)	1	50	0.02	[0.00; 0.11]	0.6%	2.3%
Han et al. (2015)	0	573	0.00	[0.00; 0.01]	6.5%	3.3%
Han et al. (2015)	0	781	0.00	[0.00; 0.00]	8.8%	3.3%
Hornok et al. (2014)	0	18	0.00	[0.00; 0.19]	0.2%	1.5%
Hornok et al. (2022)	3	29	0.10	[0.02; 0.27]	0.3%	1.9%
Hou et al. (2009)	3	476	0.01	[0.00; 0.02]	5.4%	3.2%
Daniel et al. (2015)	1	11	0.09	[0.00; 0.41]	0.1%	1.1%
Jiang et al. (2011)	43	960	0.04	[0.03; 0.06]	10.8%	3.3%
Ju et al. (2022)	0	84	0.00	[0.00; 0.04]	0.9%	2.6%
Kazimirova et al. (2018)	5	19	0.26	[0.09; 0.51]	0.2%	1.5%
Leschnik et al. (2012)	2	30	0.07	[0.01; 0.22]	0.3%	1.9%
Liang et al. (2021)	13	1359	0.01	[0.01; 0.02]	15.3%	3.3%
Liu et al. (2017)	18	412	0.04	[0.03; 0.07]	4.6%	3.2%
Polat et al. (2021)	0	63	0.00	[0.00; 0.06]	0.7%	2.5%
Pu et al. (2009)	0	727	0.00	[0.00; 0.01]	8.2%	3.3%
Raileanu et al. (2022)	0	88	0.00	[0.00; 0.04]	1.0%	2.7%
Rataud et al. (2022)	0	11	0.00	[0.00; 0.28]	0.1%	1.1%
Rigo et al. (2011)	0	28	0.00	[0.00; 0.12]	0.3%	1.8%
Rybarova et al. (2017)	11	37	0.30	[0.16; 0.47]	0.4%	2.1%
Sandor et al. (2017)	1	9	0.11	[0.00; 0.48]	0.1%	0.9%
Seo et al. (2021)	0	1	0.00	[0.00; 0.97]	0.0%	0.1%
Spitalska et al. (2021)	0	1	0.00	[0.00; 0.97]	0.0%	0.1%
Svitalkova et al. (2015)	0	60	0.00	[0.00; 0.06]	0.7%	2.4%
Szekeres et al. (2015)	2	80	0.02	[0.00; 0.09]	0.9%	2.6%
Tang et al. (2012)	14	409	0.03	[0.02; 0.06]	4.6%	3.2%
Tomanovic et al. (2012)	1	35	0.03	[0.00; 0.15]	0.4%	2.0%
Veronesi et al. (2011)	0	8	0.00	[0.00; 0.37]	0.1%	0.9%
Vichova et al. (2018)	0	36	0.00	[0.00; 0.10]	0.4%	2.0%
Wei et al. (2016)	18	412	0.04	[0.03; 0.07]	4.6%	3.2%
Wijnveld et al. (2021)	0	44	0.00	[0.00; 0.08]	0.5%	2.2%
Yang et al. (2016)	1	106	0.01	[0.00; 0.05]	1.2%	2.8%
Ye et al. (2018)	0	41	0.00	[0.00; 0.09]	0.5%	2.1%
Zhang et al. (2007)	0	220	0.00	[0.00; 0.02]	2.5%	3.1%
**Common effect model**		**8881**	**0.01**	**[0.01; 0.01]**	**100.0%**	**--**
**Random effects model**			**0.01**	**[0.00; 0.02]**	**--**	**100.0%**

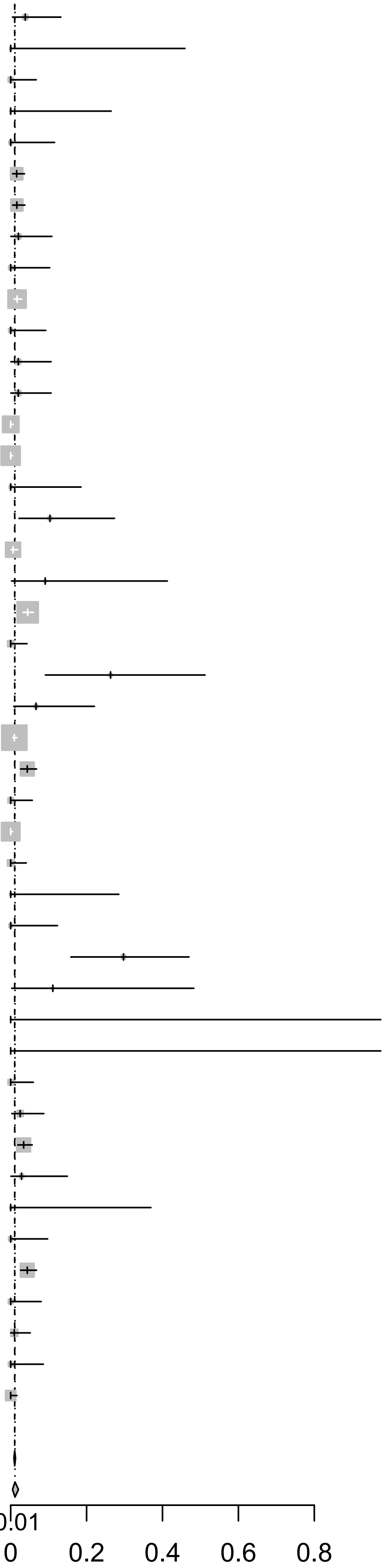

Heterogeneity: $I^2 = 85\%$, $\tau^2 = 0.0105$, $p < 0.01$

Uncharacterised *Anaplasma*

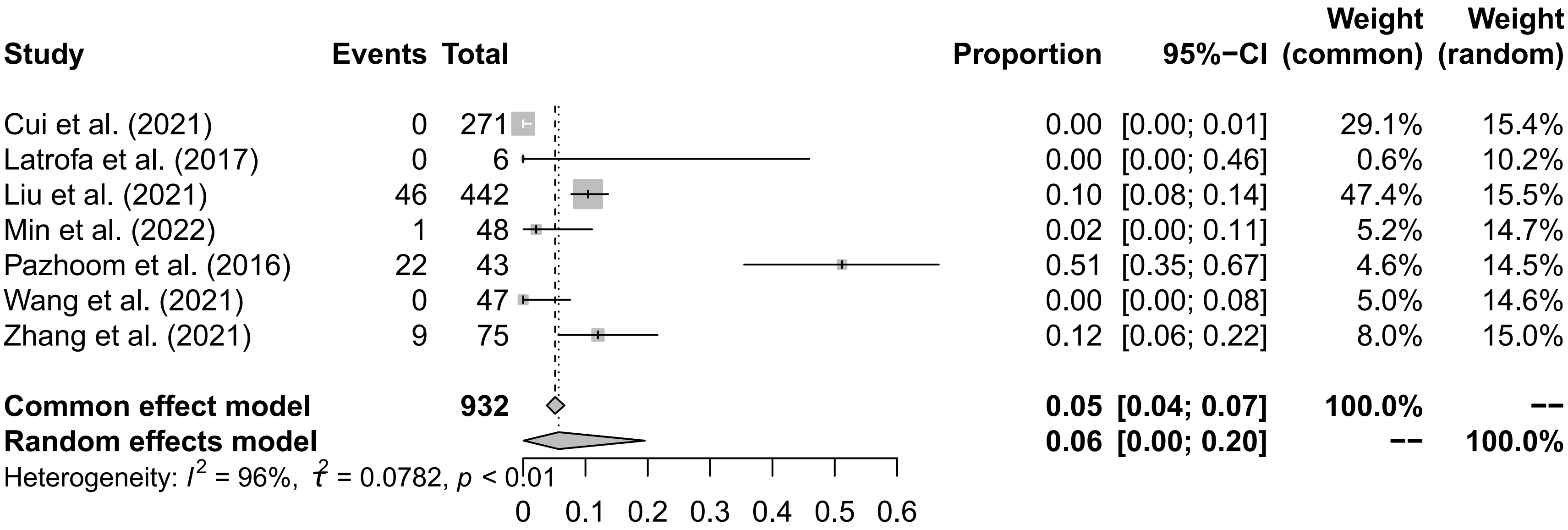

Ehrlichia chaffeensis

Study	Events	Total	Proportion	95%−CI	Weight (common)	Weight (random)
Gao et al. (2000)	0	50	0.00	[0.00; 0.07]	2.7%	4.7%
Hornok et al. (2010)	0	257	0.00	[0.00; 0.01]	13.9%	16.2%
Hou et al. (2009)	3	476	0.01	[0.00; 0.02]	25.8%	22.2%
Hu et al. (2004)	0	70	0.00	[0.00; 0.05]	3.8%	6.3%
Pu et al. (2009)	0	727	0.00	[0.00; 0.01]	39.4%	26.2%
Rataud et al. (2022)	0	11	0.00	[0.00; 0.28]	0.6%	1.1%
Seo et al. (2021)	0	1	0.00	[0.00; 0.97]	0.1%	0.1%
Wei et al. (2004)	0	70	0.00	[0.00; 0.05]	3.8%	6.3%
Wu et al. (2012)	0	74	0.00	[0.00; 0.05]	4.0%	6.6%
Ye et al. (2018)	0	41	0.00	[0.00; 0.09]	2.2%	3.9%
Zhang et al. (2005)	0	70	0.00	[0.00; 0.05]	3.8%	6.3%
**Common effect model**		**1847**	**0.00**	**[0.00; 0.00]**	**100.0%**	**--**
**Random effects model**			**0.00**	**[0.00; 0.00]**	**--**	**100.0%**

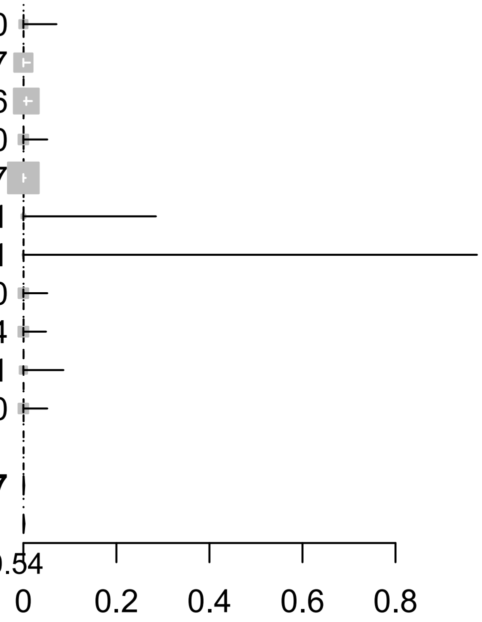

Heterogeneity: $I^2 = 0\%$, $\tau^2 = 0.0007$, $p = 0.54$

Ehrlichia muris

Study	Events	Total	Proportion	95%−CI	Weight (common)	Weight (random)
Liu et al. (2017)	1	412	0.00	[0.00; 0.01]	50.0%	50.0%
Wei et al. (2016)	1	412	0.00	[0.00; 0.01]	50.0%	50.0%
**Common effect model**		**824**	**0.00**	**[0.00; 0.01]**	**100.0%**	**--**
**Random effects model**			**0.00**	**[0.00; 0.01]**	**--**	**100.0%**

Heterogeneity: $I^2 = 0\%$, $\tau^2 = 0$, $p = 1.00$

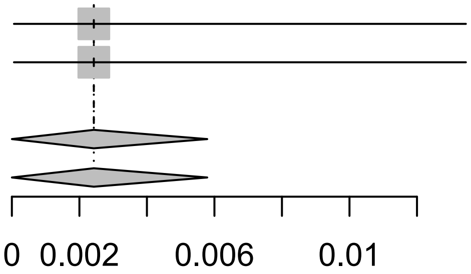

Ehrlichia sp. hc hlj209

Study	Events	Total	Proportion	95%−CI	Weight (common)	Weight (random)
Liu et al. (2017)	1	412	0.00	[0.00; 0.01]	50.0%	50.0%
Wei et al. (2016)	1	412	0.00	[0.00; 0.01]	50.0%	50.0%
**Common effect model**		**824**	**0.00**	**[0.00; 0.01]**	**100.0%**	**−−**
**Random effects model**			**0.00**	**[0.00; 0.01]**	**−−**	**100.0%**

Heterogeneity: $I^2 = 0\%$, $\tau^2 = 0$, $p = 1.00$

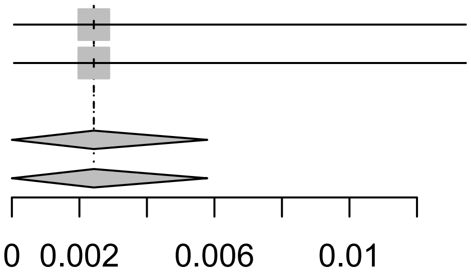

Uncharacterised *Ehrlichia*

Study	Events	Total	Proportion	95%−CI	Weight (common)	Weight (random)
Chang et al. (2021)	0	35	0.00	[0.00; 0.10]	1.4%	7.7%
Cui et al. (2021)	23	271	0.08	[0.05; 0.12]	10.8%	10.9%
Fu et al. (2015)	0	38	0.00	[0.00; 0.09]	1.5%	7.9%
Han et al. (2019)	0	18	0.00	[0.00; 0.19]	0.7%	5.9%
Jian et al. (2002)	0	32	0.00	[0.00; 0.11]	1.3%	7.5%
Ju et al. (2022)	0	84	0.00	[0.00; 0.04]	3.4%	9.6%
Liang et al. (2021)	3	1359	0.00	[0.00; 0.01]	54.3%	11.5%
Liu et al. (2021)	34	442	0.08	[0.05; 0.11]	17.7%	11.2%
Wang et al. (2021)	2	47	0.04	[0.01; 0.15]	1.9%	8.5%
Wei et al. (2001)	0	70	0.00	[0.00; 0.05]	2.8%	9.3%
Yang et al. (2016)	0	106	0.00	[0.00; 0.03]	4.2%	10.0%
**Common effect model**		**2502**	**0.01**	**[0.01; 0.02]**	**100.0%**	**−−**
**Random effects model**			**0.01**	**[0.00; 0.03]**	**−−**	**100.0%**

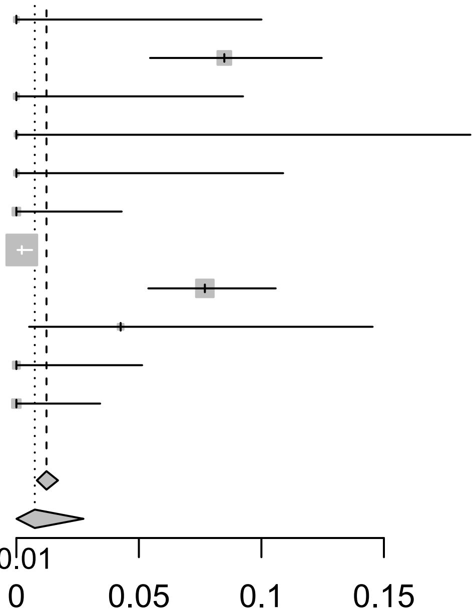

Heterogeneity: $I^2 = 92\%$, $\tau^2 = 0.0141$, $p < 0.01$

Candidatus Neoehrlichia mikurensis

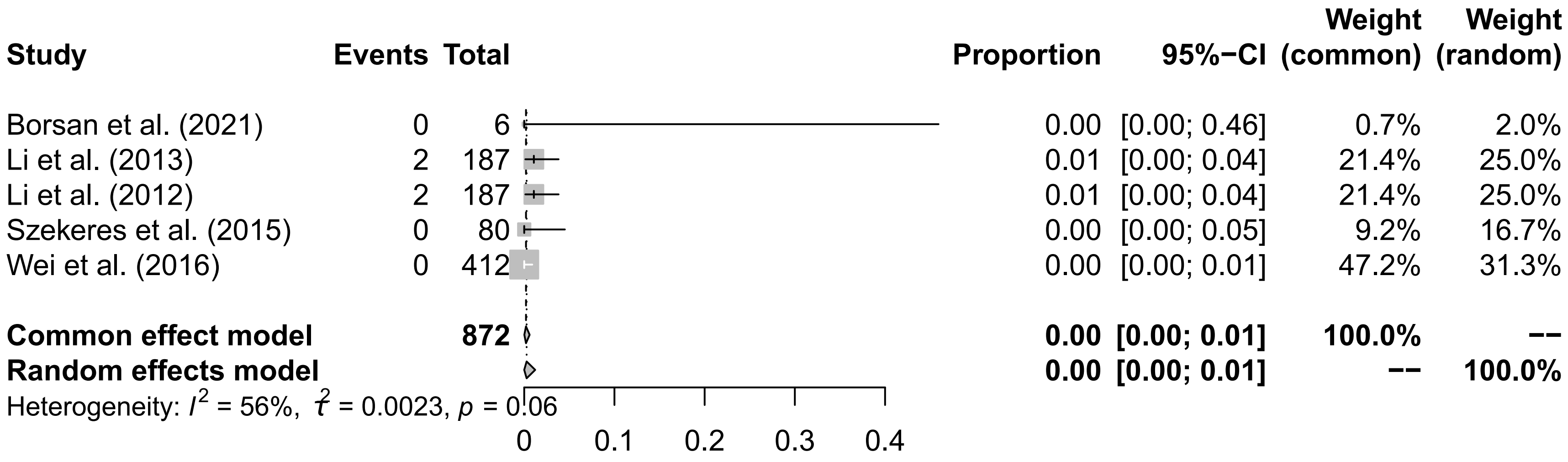

Babesia bigemina

Study	Events	Total	Proportion	95%−CI	Weight (common)	Weight (random)
Jiang et al. (2016)	1	553	0.00	[0.00; 0.01]	67.5%	67.5%
Xia et al. (2020)	1	384	0.00	[0.00; 0.01]	32.5%	32.5%
**Common effect model**		**937**	**0.00**	**[0.00; 0.00]**	**100.0%**	**--**
**Random effects model**			**0.00**	**[0.00; 0.00]**	**--**	**100.0%**

Heterogeneity: $I^2 = 0\%$, $\tau^2 = 0$, $p = 0.80$

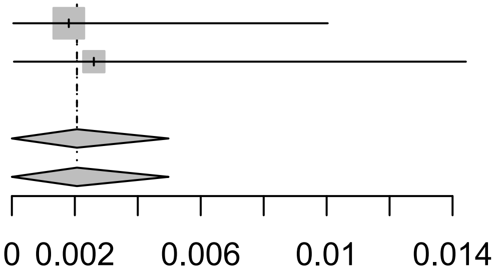

Babesia canis

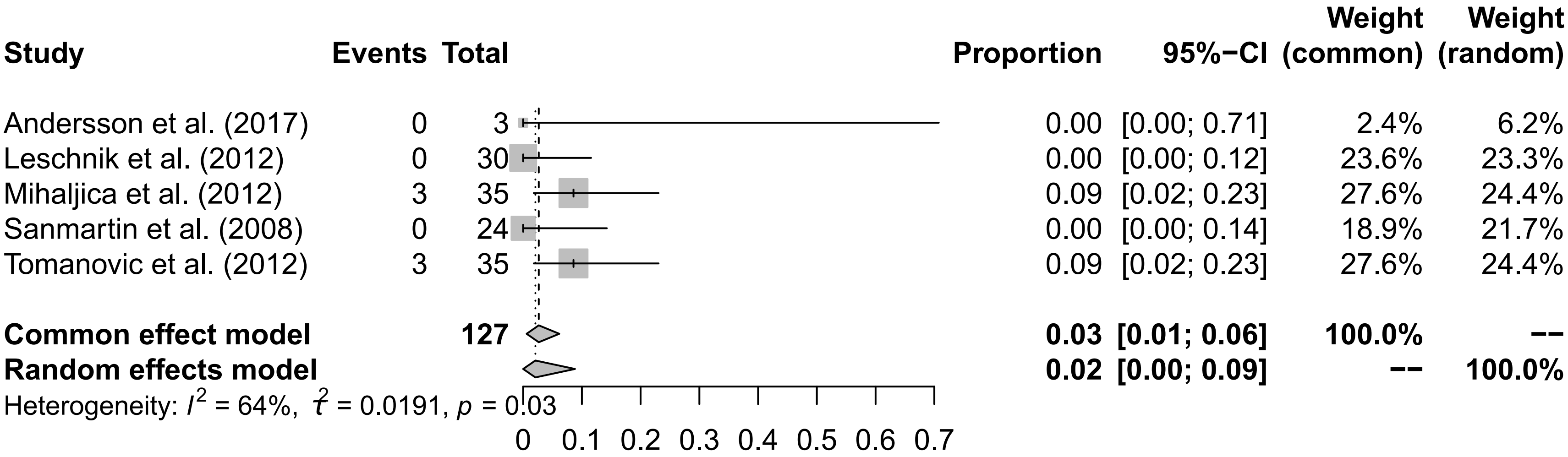

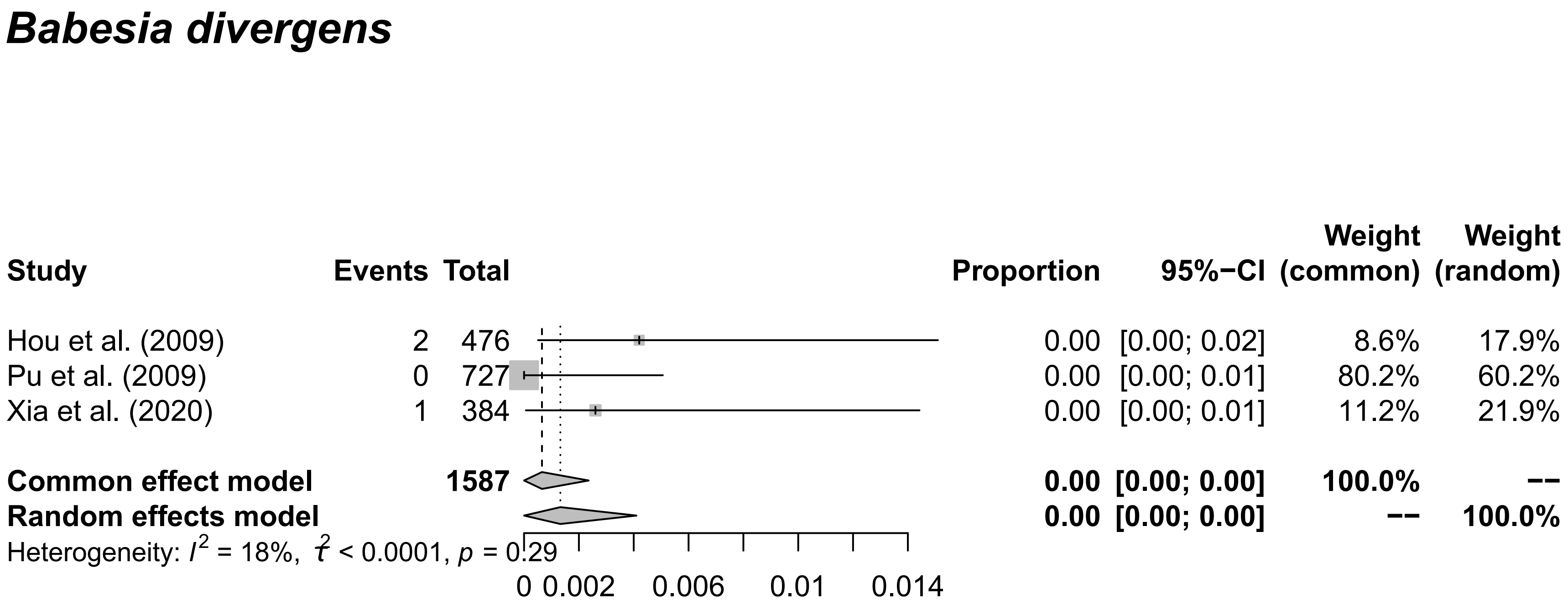
Babesia divergens
Study
Events
Total
Proportion
95%−CI
Weight (common)
Weight (random)
Hou et al. (2009) 2 476 0.00 [0.00; 0.02] 8.6% 17.9%
Pu et al. (2009) 0 727 0.00 [0.00; 0.01] 80.2% 60.2%
Xia et al. (2020) 1 384 0.00 [0.00; 0.01] 11.2% 21.9%
Common effect model 1587 0.00 [0.00; 0.00] 100.0% --
Random effects model 0.00 [0.00; 0.00] -- 100.0%
Heterogeneity: I^2 = 18%, τ^2 < 0.0001, p = 0.29
0
0.002
0.006
0.01
0.014

Babesia microti

Study	Events	Total	Proportion	95%−CI	Weight (common)	Weight (random)
Andersson et al. (2017)	0	3	0.00	[0.00; 0.71]	0.1%	6.2%
Hamsikova et al. (2016)	4	5	0.80	[0.28; 0.99]	0.2%	8.1%
Hou et al. (2009)	9	476	0.02	[0.01; 0.04]	17.3%	14.6%
Jiang et al. (2016)	0	553	0.00	[0.00; 0.01]	20.1%	14.6%
Pu et al. (2009)	0	727	0.00	[0.00; 0.01]	26.5%	14.6%
Rar et al. (2014)	0	573	0.00	[0.00; 0.01]	20.9%	14.6%
Sanmartin et al. (2008)	0	24	0.00	[0.00; 0.14]	0.9%	12.6%
Xia et al. (2020)	0	384	0.00	[0.00; 0.01]	14.0%	14.6%
**Common effect model**		**2745**	**0.00**	**[0.00; 0.00]**	**100.0%**	**--**
**Random effects model**			**0.01**	**[0.00; 0.09]**	**--**	**100.0%**

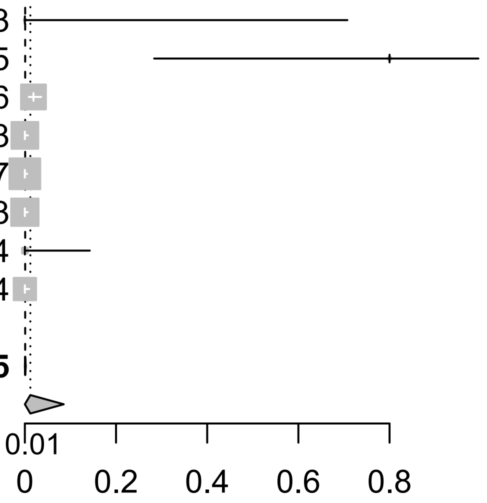

Heterogeneity: $I^2 = 87\%$, $\tau^2 = 0.0614$, $p < 0.01$

Babesia sp. Bime

Study	Events	Total	Proportion	95%−CI	Weight (common)	Weight (random)
Jia et al. (2018)	1	252	0.00	[0.00; 0.02]	59.0%	54.6%
Jiang et al. (2016)	7	553	0.01	[0.01; 0.03]	41.0%	45.4%
**Common effect model**		**805**	**0.01**	**[0.00; 0.01]**	**100.0%**	**--**
**Random effects model**			**0.01**	**[0.00; 0.02]**	**--**	**100.0%**

Heterogeneity: $I^2 = 49\%$, $\tau^2 < 0.0001$, $p = 0.16$

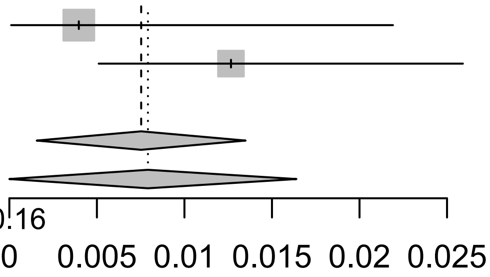

Uncharacterised *Babesia*

Study	Events	Total	Proportion	95%−CI	Weight (common)	Weight (random)
Andersson et al. (2018)	0	1	0.00	[0.00; 0.97]	0.0%	0.3%
Blaschitz et al. (2008)	0	11	0.00	[0.00; 0.28]	0.2%	1.9%
Cerro et al. (2022)	0	53	0.00	[0.00; 0.07]	0.9%	3.7%
Chen et al. (2016)	0	12	0.00	[0.00; 0.26]	0.2%	2.0%
Chen et al. (2016)	0	30	0.00	[0.00; 0.12]	0.5%	3.1%
Cheng et al. (2017)	0	308	0.00	[0.00; 0.01]	5.5%	4.6%
Cheng et al. (2015)	0	49	0.00	[0.00; 0.07]	0.9%	3.6%
Flaisz et al. (2017)	51	321	0.16	[0.12; 0.20]	5.7%	4.7%
Fu et al. (2015)	0	38	0.00	[0.00; 0.09]	0.7%	3.3%
Hamsikova et al. (2016)	0	7	0.00	[0.00; 0.41]	0.1%	1.4%
Han et al. (2016)	0	55	0.00	[0.00; 0.06]	1.0%	3.7%
Han et al. (2015)	0	781	0.00	[0.00; 0.00]	13.9%	4.8%
Kazimirova et al. (2018)	3	13	0.23	[0.05; 0.54]	0.2%	2.1%
Liang et al. (2021)	0	1359	0.00	[0.00; 0.00]	24.2%	4.8%
Min et al. (2022)	0	48	0.00	[0.00; 0.07]	0.9%	3.6%
Polat et al. (2021)	0	63	0.00	[0.00; 0.06]	1.1%	3.8%
Raileanu et al. (2022)	7	88	0.08	[0.03; 0.16]	1.6%	4.1%
Rar et al. (2014)	17	573	0.03	[0.02; 0.05]	10.2%	4.8%
Rataud et al. (2022)	0	11	0.00	[0.00; 0.28]	0.2%	1.9%
Rybarova et al. (2017)	6	150	0.04	[0.01; 0.09]	2.7%	4.4%
Spitalska et al. (2021)	0	1	0.00	[0.00; 0.97]	0.0%	0.3%
Sun et al. (2019)	0	45	0.00	[0.00; 0.08]	0.8%	3.5%
Svehlova et al. (2014)	0	4	0.00	[0.00; 0.60]	0.1%	0.9%
Szarek et al. (2021)	5	427	0.01	[0.00; 0.03]	7.6%	4.7%
Wang et al. (2021)	2	47	0.04	[0.01; 0.15]	0.8%	3.6%
Wijnveld et al. (2021)	4	44	0.09	[0.03; 0.22]	0.8%	3.5%
Xia et al. (2020)	11	461	0.02	[0.01; 0.04]	8.2%	4.7%
Yang et al. (2016)	0	106	0.00	[0.00; 0.03]	1.9%	4.2%
Ye et al. (2018)	0	41	0.00	[0.00; 0.09]	0.7%	3.4%
Zhang et al. (2013)	13	461	0.03	[0.02; 0.05]	8.2%	4.7%
**Common effect model**		**5608**	**0.01**	**[0.01; 0.01]**	**100.0%**	**--**
**Random effects model**			**0.01**	**[0.00; 0.02]**	**--**	**100.0%**

Heterogeneity: $I^2 = 91\%$, $\tau^2 = 0.0141$, $p < 0.01$

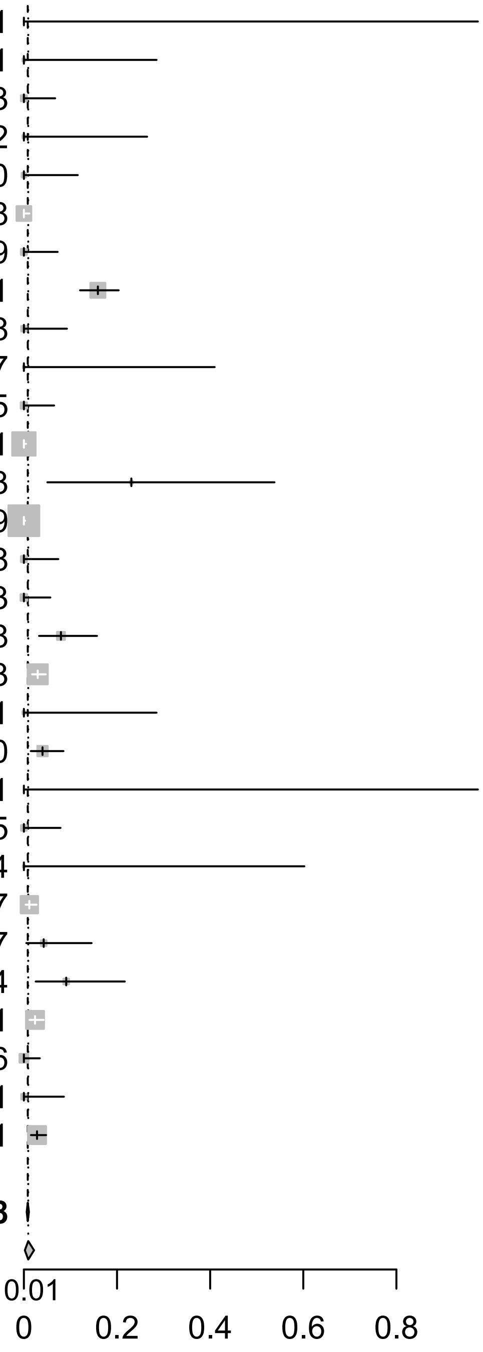

Uncharacterised *Bartonella*

Study	Events	Total	Proportion	95%−CI	Weight (common)	Weight (random)
Chen et al. (2022)	4	98	0.04	[0.01; 0.10]	4.1%	9.6%
Cheng et al. (2017)	0	308	0.00	[0.00; 0.01]	13.0%	13.6%
Cheng et al. (2015)	0	49	0.00	[0.00; 0.07]	2.1%	6.7%
Fu et al. (2015)	0	38	0.00	[0.00; 0.09]	1.6%	5.7%
Han et al. (2016)	0	55	0.00	[0.00; 0.06]	2.3%	7.2%
Ju et al. (2016)	0	41	0.00	[0.00; 0.09]	1.7%	6.0%
Liang et al. (2021)	12	1359	0.01	[0.00; 0.02]	57.4%	16.0%
Seo et al. (2021)	0	1	0.00	[0.00; 0.97]	0.0%	0.2%
Sonnberge et al. (2022)	3	5	0.60	[0.15; 0.95]	0.2%	1.1%
Tomanovic et al. (2012)	0	35	0.00	[0.00; 0.10]	1.5%	5.4%
Vichova et al. (2018)	0	36	0.00	[0.00; 0.10]	1.5%	5.5%
Yang et al. (2016)	0	106	0.00	[0.00; 0.03]	4.5%	9.9%
Ye et al. (2018)	0	236	0.00	[0.00; 0.02]	10.0%	12.9%
**Common effect model**		**2367**	**0.00**	**[0.00; 0.01]**	**100.0%**	**--**
**Random effects model**			**0.00**	**[0.00; 0.01]**	**--**	**100.0%**

Heterogeneity: $I^2 = 71\%$, $\tau^2 = 0.0034$, $p < 0.01$

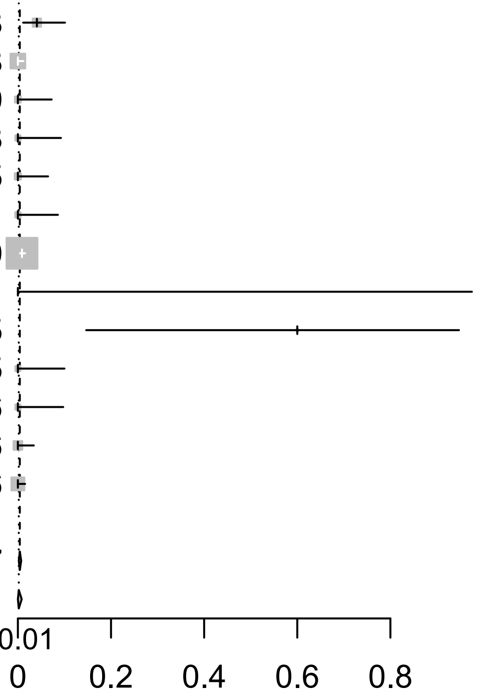

Borreliella afzelii

Study	Events	Total	Proportion	95%-CI	Weight (common)	Weight (random)
Chu et al. (2008)	0	2	0.00	[0.00; 0.84]	0.1%	1.3%
Li et al. (2010)	23	356	0.06	[0.04; 0.10]	23.4%	26.4%
Pu et al. (2009)	8	727	0.01	[0.00; 0.02]	47.8%	27.9%
Rigo et al. (2011)	3	388	0.01	[0.00; 0.02]	25.5%	26.6%
Wijnveld et al. (2021)	1	44	0.02	[0.00; 0.12]	2.9%	15.1%
Zhou et al. (2017)	1	4	0.25	[0.01; 0.81]	0.3%	2.6%
**Common effect model**		**1521**	**0.02**	**[0.01; 0.03]**	**100.0%**	**--**
**Random effects model**			**0.02**	**[0.01; 0.06]**	**--**	**100.0%**

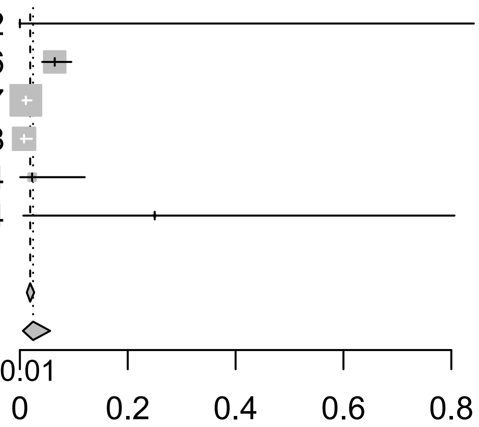

Heterogeneity: $I^2 = 83\%$, $\tau^2 = 0.0060$, $p < 0.01$

Borrelia burgdorferi

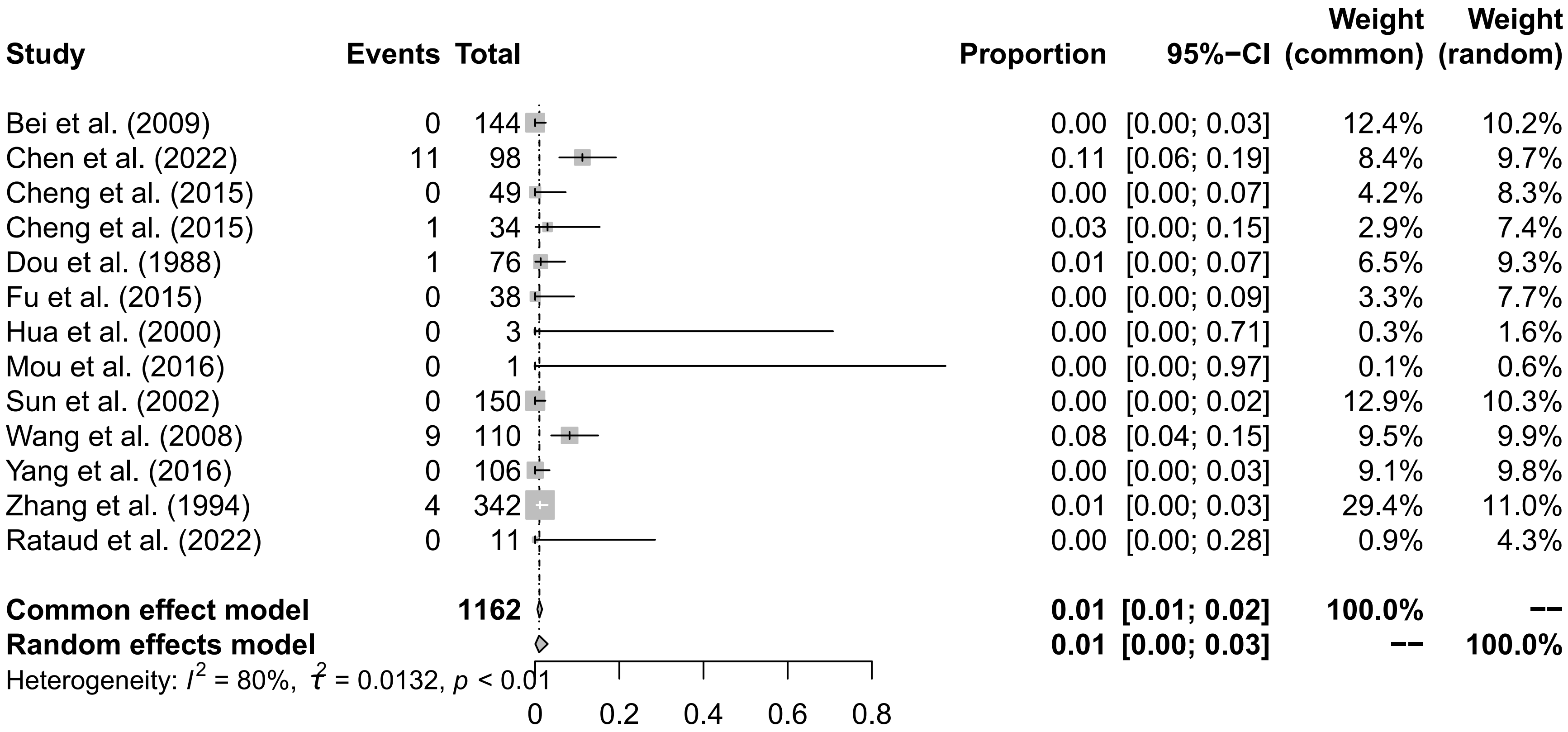

Borrelia garinii

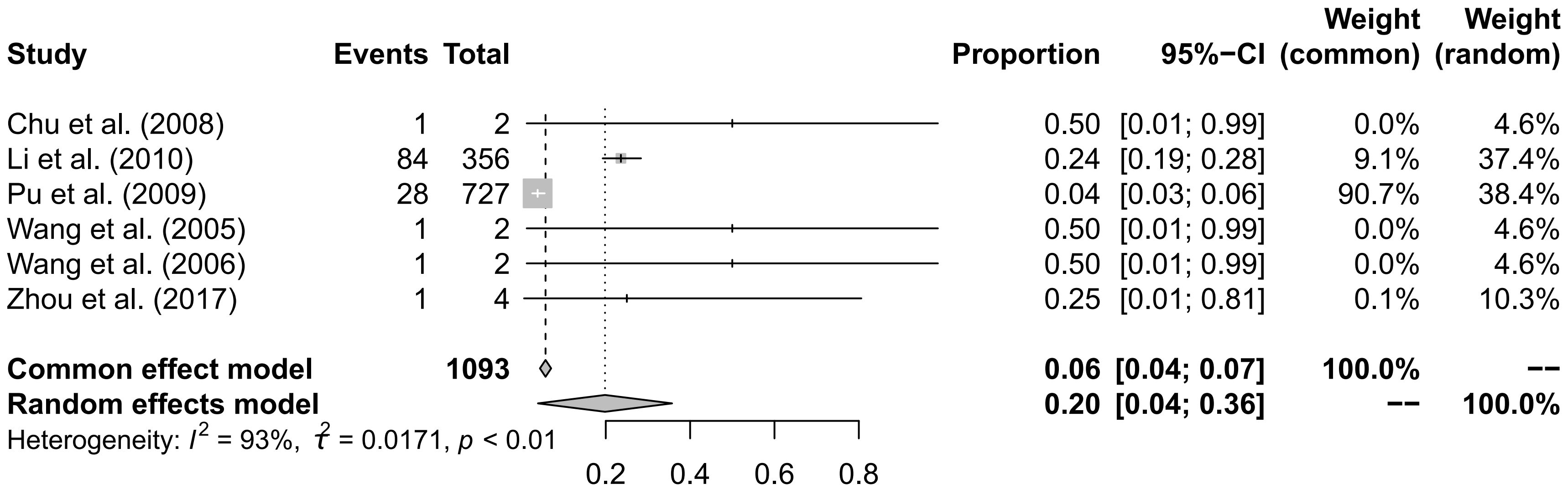

Borrelia miyamotoi

Study	Events	Total	Proportion	95%−CI	Weight (common)	Weight (random)
Heglasova et al. (2020)	0	93	0.00	[0.00; 0.04]	12.9%	16.6%
Jiang et al. (2018)	1	36	0.03	[0.00; 0.15]	5.0%	12.9%
Liu et al. (2021)	35	442	0.08	[0.06; 0.11]	61.3%	19.3%
Liu et al. (2018)	1	36	0.03	[0.00; 0.15]	5.0%	12.9%
Rataud et al. (2022)	0	11	0.00	[0.00; 0.28]	1.5%	7.1%
Szekeres et al. (2015)	0	72	0.00	[0.00; 0.05]	10.0%	15.8%
Tang et al. (2018)	0	5	0.00	[0.00; 0.52]	0.7%	4.0%
Zhou et al. (2017)	0	26	0.00	[0.00; 0.13]	3.6%	11.4%
**Common effect model**		**721**	**0.04**	**[0.02; 0.05]**	**100.0%**	**--**
**Random effects model**			**0.01**	**[0.00; 0.04]**	**--**	**100.0%**

Heterogeneity: $I^2 = 85\%$, $\tau^2 = 0.0125$, $p < 0.01$

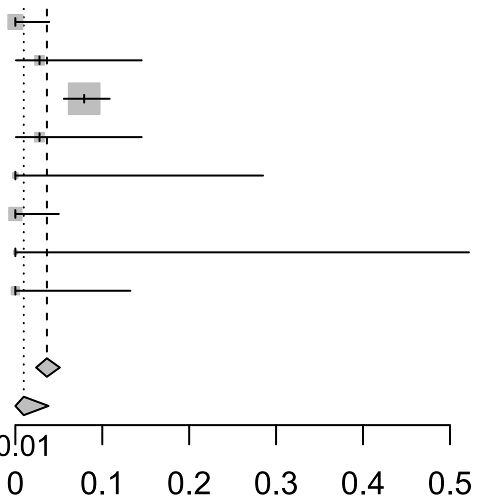

Uncharacterised *Borreliella*

Study	Events	Total	Proportion	95%−CI	Weight (common)	Weight (random)
Jiang et al. (2006)	0	2	0.00	[0.00; 0.84]	0.0%	0.5%
Liang et al. (2021)	32	1359	0.02	[0.02; 0.03]	17.0%	2.9%
Liu et al. (2021)	187	442	0.42	[0.38; 0.47]	5.5%	2.9%
Yang et al. (2006)	9	110	0.08	[0.04; 0.15]	1.4%	2.7%
Andersson et al. (2018)	0	1	0.00	[0.00; 0.97]	0.0%	0.3%
Barandika et al. (2008)	0	52	0.00	[0.00; 0.07]	0.7%	2.5%
Blaschitz et al. (2008)	0	11	0.00	[0.00; 0.28]	0.1%	1.5%
Borsan et al. (2021)	0	6	0.00	[0.00; 0.46]	0.1%	1.1%
Cai et al. (1994)	0	10	0.00	[0.00; 0.31]	0.1%	1.4%
Cerro et al. (2022)	0	53	0.00	[0.00; 0.07]	0.7%	2.5%
Chen et al. (2016)	4	12	0.33	[0.10; 0.65]	0.2%	1.6%
Chen et al. (2016)	5	30	0.17	[0.06; 0.35]	0.4%	2.2%
Cheng et al. (2017)	0	308	0.00	[0.00; 0.01]	3.9%	2.9%
Chu et al. (2005)	0	144	0.00	[0.00; 0.03]	1.8%	2.8%
Fu et al. (2008)	6	180	0.03	[0.01; 0.07]	2.3%	2.8%
Han et al. (2015)	1	573	0.00	[0.00; 0.01]	7.2%	2.9%
Han et al. (2015)	1	781	0.00	[0.00; 0.01]	9.8%	2.9%
Hou et al. (2009)	22	476	0.05	[0.03; 0.07]	6.0%	2.9%
Hubalek et al. (1996)	0	13	0.00	[0.00; 0.25]	0.2%	1.6%
Hubalek et al. (1998)	1	249	0.00	[0.00; 0.02]	3.1%	2.8%
Hubalek et al. (2003)	0	22	0.00	[0.00; 0.15]	0.3%	2.0%
Kahl et al. (1992)	0	96	0.00	[0.00; 0.04]	1.2%	2.7%
Leschnik et al. (2012)	1	30	0.03	[0.00; 0.17]	0.4%	2.2%
Polat et al. (2021)	0	63	0.00	[0.00; 0.06]	0.8%	2.5%
Pukhovskaya et al. (2019)	14	481	0.03	[0.02; 0.05]	6.0%	2.9%
Raileanu et al. (2022)	0	88	0.00	[0.00; 0.04]	1.1%	2.6%
Seo et al. (2021)	0	1	0.00	[0.00; 0.97]	0.0%	0.3%
Sonnberge et al. (2022)	0	5	0.00	[0.00; 0.52]	0.1%	1.0%
Spitalska et al. (2021)	0	1	0.00	[0.00; 0.97]	0.0%	0.3%
Sun et al. (2001)	0	150	0.00	[0.00; 0.02]	1.9%	2.8%
Szarek et al. (2021)	28	427	0.07	[0.04; 0.09]	5.3%	2.9%
Szekeres et al. (2015)	0	72	0.00	[0.00; 0.05]	0.9%	2.6%
Tang et al. (2012)	14	409	0.03	[0.02; 0.06]	5.1%	2.9%
Tang et al. (2012)	3	108	0.03	[0.01; 0.08]	1.4%	2.7%
Tang et al. (2018)	0	5	0.00	[0.00; 0.52]	0.1%	1.0%
Tao et al. (1991)	0	120	0.00	[0.00; 0.03]	1.5%	2.7%
Tomanovic et al. (2012)	0	35	0.00	[0.00; 0.10]	0.4%	2.3%
Wan et al. (1998)	7	50	0.14	[0.06; 0.27]	0.6%	2.4%
Wang et al. (2005)	0	200	0.00	[0.00; 0.02]	2.5%	2.8%
Wang et al. (2019)	0	412	0.00	[0.00; 0.01]	5.2%	2.9%
Wang et al. (2021)	2	47	0.04	[0.01; 0.15]	0.6%	2.4%
Ye et al. (2018)	0	41	0.00	[0.00; 0.09]	0.5%	2.4%
Zhang et al. (1992)	4	250	0.02	[0.00; 0.04]	3.1%	2.8%
Zhou et al. (2017)	0	22	0.00	[0.00; 0.15]	0.3%	2.0%
Zhuang et al. (1994)	0	47	0.00	[0.00; 0.08]	0.6%	2.4%
**Common effect model**		**7994**	**0.02**	**[0.02; 0.02]**	**100.0%**	**--**
**Random effects model**			**0.01**	**[0.00; 0.03]**	**--**	**100.0%**

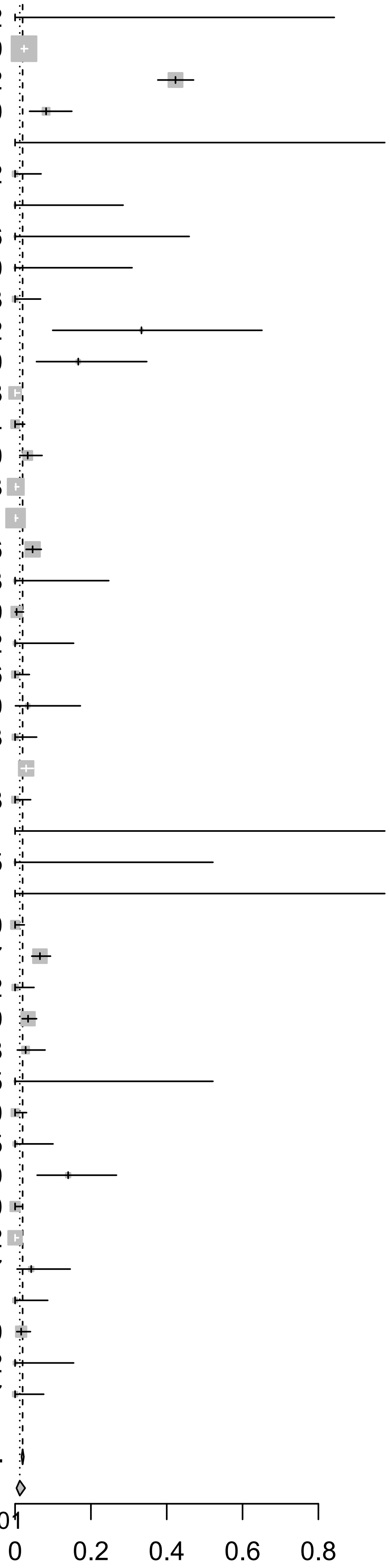

Heterogeneity: $I^2 = 95\%$, $\tau^2 = 0.0238$, $p < 0.01$

Coxiella burnetii

Study	Events	Total	Proportion	95%−CI	Weight (common)	Weight (random)
Andoh et al. (2013)	0	4	0.00	[0.00; 0.60]	0.1%	1.8%
Barandika et al. (2008)	0	52	0.00	[0.00; 0.07]	1.2%	5.8%
Cerro et al. (2022)	0	53	0.00	[0.00; 0.07]	1.2%	5.8%
Fu et al. (2015)	1	38	0.03	[0.00; 0.14]	0.9%	5.5%
Guo et al. (2013)	4	143	0.03	[0.01; 0.07]	3.3%	6.6%
Gyuranecz et al. (2012)	0	735	0.00	[0.00; 0.01]	16.9%	7.0%
Han et al. (2016)	3	55	0.05	[0.01; 0.15]	1.3%	5.9%
Liang et al. (2021)	4	1359	0.00	[0.00; 0.01]	31.2%	7.0%
Lu et al. (2021)	0	20	0.00	[0.00; 0.17]	0.5%	4.5%
Luo et al. (2021)	7	112	0.06	[0.03; 0.12]	2.6%	6.4%
Minichova et al. (2017)	0	61	0.00	[0.00; 0.06]	1.4%	6.0%
Pu et al. (2009)	11	727	0.02	[0.01; 0.03]	16.7%	7.0%
Rahravani et al. (2022)	3	24	0.12	[0.03; 0.32]	0.6%	4.8%
Spitalska et al. (2003)	3	344	0.01	[0.00; 0.03]	7.9%	6.9%
Tomanovic et al. (2012)	10	35	0.29	[0.15; 0.46]	0.8%	5.4%
Ye et al. (2018)	0	236	0.00	[0.00; 0.02]	5.4%	6.8%
Zhang et al. (2011)	48	356	0.13	[0.10; 0.17]	8.2%	6.9%
**Common effect model**		**4354**	**0.01**	**[0.01; 0.01]**	**100.0%**	**--**
**Random effects model**			**0.02**	**[0.00; 0.05]**	**--**	**100.0%**

Heterogeneity: $I^2 = 93\%$, $\tau^2 = 0.0220$, $p < 0.01$

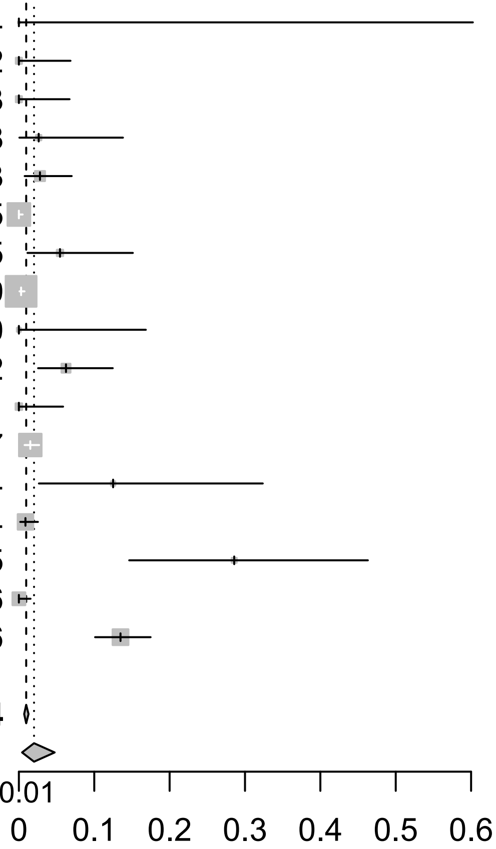

Francisella tularensis

Study	Events	Total	Proportion	95%−CI	Weight (common)	Weight (random)
Fu et al. (2015)	0	38	0.00	[0.00; 0.09]	0.6%	3.9%
Gurycova et al. (1995)	1	35	0.03	[0.00; 0.15]	0.6%	3.7%
Gyuranecz et al. (2011)	2	504	0.00	[0.00; 0.01]	8.5%	7.4%
Hubalek et al. (1990)	0	39	0.00	[0.00; 0.09]	0.7%	3.9%
Hubalek et al. (1996)	0	60	0.00	[0.00; 0.06]	1.0%	4.8%
Hubalek et al. (1997)	0	60	0.00	[0.00; 0.06]	1.0%	4.8%
Jia et al. (2011)	17	356	0.05	[0.03; 0.08]	6.0%	7.2%
Kreizinger et al. (2013)	2	735	0.00	[0.00; 0.01]	12.4%	7.6%
Lancz et al. (2009)	0	194	0.00	[0.00; 0.02]	3.3%	6.6%
Li et al. (2014)	17	356	0.05	[0.03; 0.08]	6.0%	7.2%
Liang et al. (2021)	1	1359	0.00	[0.00; 0.00]	22.9%	7.7%
Rahravani et al. (2022)	0	24	0.00	[0.00; 0.14]	0.4%	3.0%
Sonnberge et al. (2022)	0	5	0.00	[0.00; 0.52]	0.1%	0.9%
Tomanovic et al. (2012)	0	35	0.00	[0.00; 0.10]	0.6%	3.7%
Wang et al. (2022)	1	107	0.01	[0.00; 0.05]	1.8%	5.8%
Wei et al. (2013)	20	1434	0.01	[0.01; 0.02]	24.2%	7.8%
Ye et al. (2018)	0	236	0.00	[0.00; 0.02]	4.0%	6.8%
Zheng et al. (2018)	17	356	0.05	[0.03; 0.08]	6.0%	7.2%
**Common effect model**		**5933**	**0.01**	**[0.01; 0.01]**	**100.0%**	**--**
**Random effects model**			**0.01**	**[0.00; 0.02]**	**--**	**100.0%**

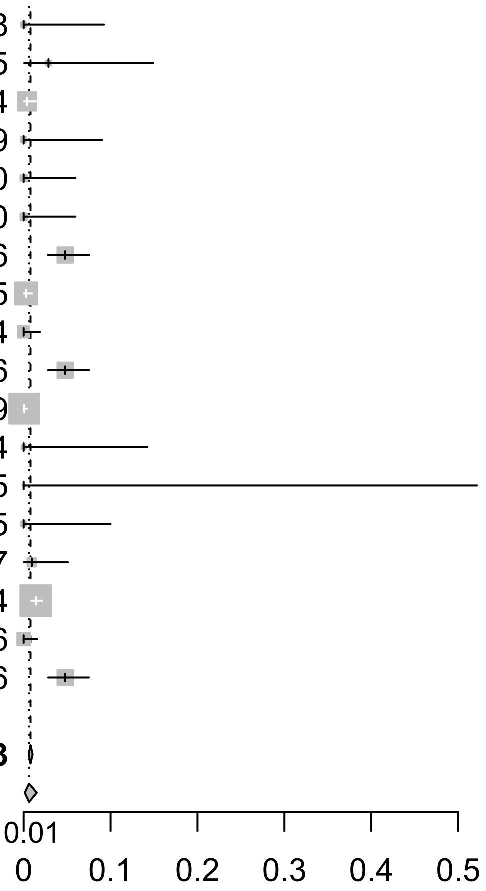

Heterogeneity: $I^2 = 87\%$, $\tau^2 = 0.0063$, $p < 0.01$

Hepatozoon canis

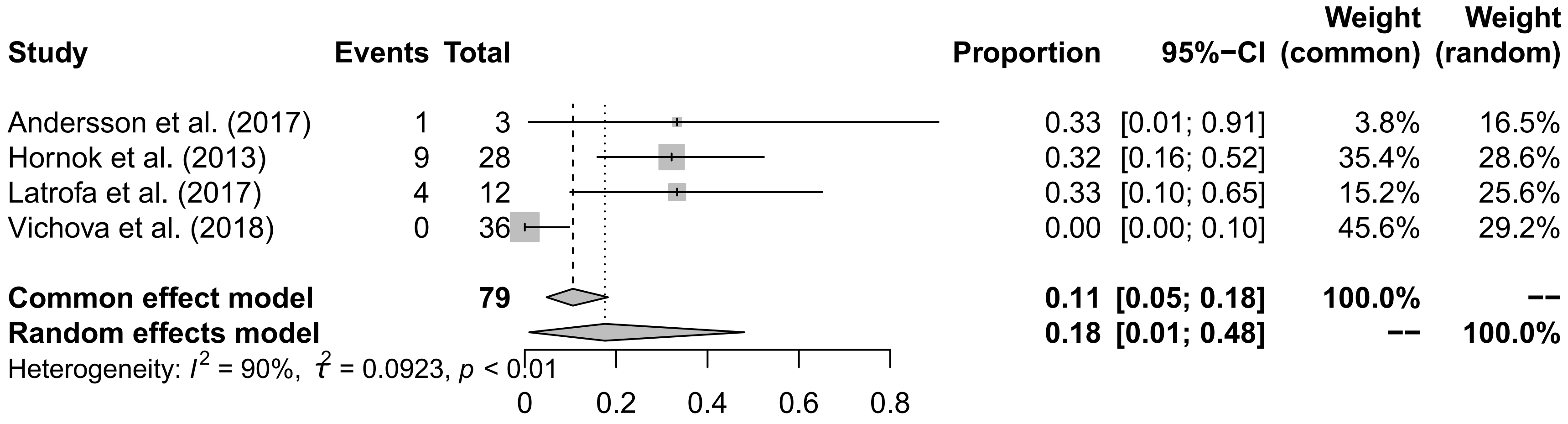

Uncharacterised *Hepatozoon*

Study	Events	Total	Proportion	95%−CI	Weight (common)	Weight (random)
Andersson et al. (2018)	0	1	0.00	[0.00; 0.97]	0.0%	0.0%
Borsan et al. (2021)	0	6	0.00	[0.00; 0.46]	0.1%	0.1%
Hornok et al. (2022)	2	259	0.01	[0.00; 0.03]	32.0%	32.0%
Li et al. (2018)	2	378	0.01	[0.00; 0.02]	67.9%	67.9%
**Common effect model**		**644**	**0.01**	**[0.00; 0.01]**	**100.0%**	**--**
**Random effects model**			**0.01**	**[0.00; 0.01]**	**--**	**100.0%**

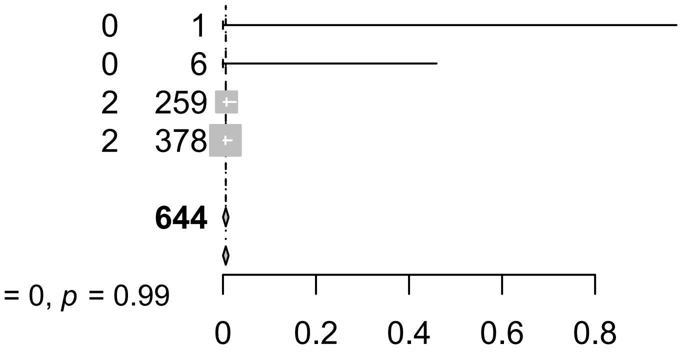

Heterogeneity: $I^2 = 0\%$, $\tau^2 = 0$, $p = 0.99$

Rickettsia heilongjiangensis

Study	Events	Total	Proportion	95%−CI	Weight (common)	Weight (random)
Ando et al. (2010)	3	15	0.20	[0.04; 0.48]	0.0%	2.4%
Chen et al. (2000)	1	100	0.01	[0.00; 0.05]	3.5%	7.5%
Cheng et al. (2019)	32	211	0.15	[0.11; 0.21]	0.6%	6.8%
Cheng et al. (2016)	17	55	0.31	[0.19; 0.45]	0.1%	4.3%
Cheng et al. (2017)	32	308	0.10	[0.07; 0.14]	1.1%	7.2%
Cui et al. (2021)	8	271	0.03	[0.01; 0.06]	3.2%	7.5%
Hou et al. (2009)	1	476	0.00	[0.00; 0.01]	77.6%	7.6%
Igolkina et al. (2018)	46	439	0.10	[0.08; 0.14]	1.6%	7.3%
Liu et al. (2017)	15	412	0.04	[0.02; 0.06]	4.0%	7.5%
Mediannikov et al. (2006)	9	32	0.28	[0.14; 0.47]	0.1%	3.4%
Mediannikov et al. (2009)	2	31	0.06	[0.01; 0.21]	0.2%	5.5%
Sheng et al. (2021)	5	136	0.04	[0.01; 0.08]	1.3%	7.3%
Shpynov et al. (2004)	1	38	0.03	[0.00; 0.14]	0.5%	6.7%
Shpynov et al. (2009)	4	20	0.20	[0.06; 0.44]	0.0%	2.9%
Wang et al. (2018)	17	530	0.03	[0.02; 0.05]	5.8%	7.6%
Yang et al. (2016)	16	106	0.15	[0.09; 0.23]	0.3%	6.1%
Zhou et al. (2017)	2	12	0.17	[0.02; 0.48]	0.0%	2.3%
**Common effect model**		**3192**	**0.01**	**[0.01; 0.02]**	**100.0%**	**--**
**Random effects model**			**0.09**	**[0.05; 0.13]**	**--**	**100.0%**

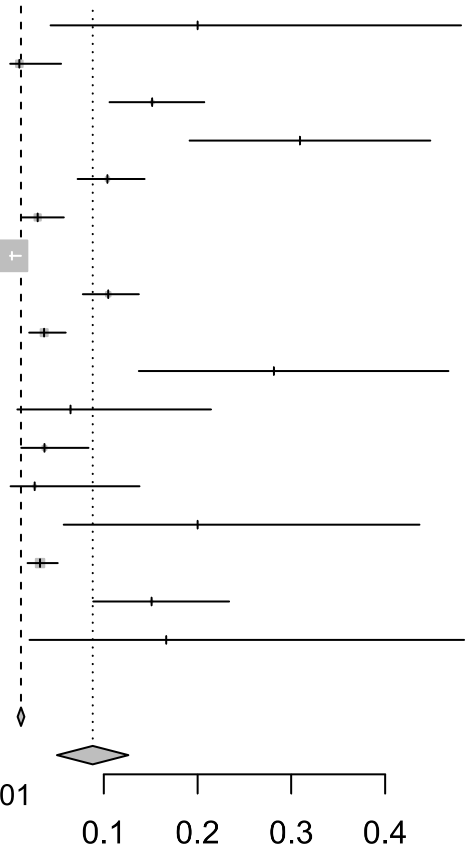

Heterogeneity: $I^2 = 92\%$, $\tau^2 = 0.0049$, $p < 0.01$

Rickettsia helvetica

Study	Events	Total	Proportion	95%−CI	Weight (common)	Weight (random)
Hornok et al. (2014)	7	18	0.39	[0.17; 0.64]	13.6%	23.6%
Radulovic et al. (2011)	0	35	0.00	[0.00; 0.10]	26.5%	25.3%
Tomanovic et al. (2012)	0	35	0.00	[0.00; 0.10]	26.5%	25.3%
Wijnveld et al. (2021)	2	44	0.05	[0.01; 0.15]	33.3%	25.7%
**Common effect model**		**132**	**0.03**	**[0.01; 0.06]**	**100.0%**	**--**
**Random effects model**			**0.05**	**[0.00; 0.24]**	**--**	**100.0%**

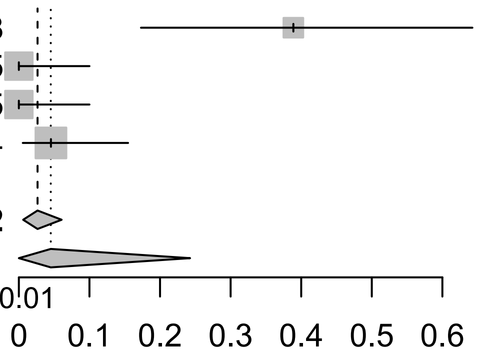

Heterogeneity: $I^2 = 89\%$, $\tau^2 = 0.0858$, $p < 0.01$

Rickettsia monacensis

Study	Events	Total	Proportion	95%−CI	Weight (common)	Weight (random)
Borsan et al. (2021)	1	6	0.17	[0.00; 0.64]	6.2%	6.3%
Hornok et al. (2014)	0	18	0.00	[0.00; 0.19]	18.7%	18.8%
Marcutan et al. (2016)	1	2	0.50	[0.01; 0.99]	2.1%	2.1%
Radulovic et al. (2011)	0	35	0.00	[0.00; 0.10]	36.5%	36.5%
Tomanovic et al. (2012)	0	35	0.00	[0.00; 0.10]	36.5%	36.5%
**Common effect model**		**96**	**0.00**	**[0.00; 0.02]**	**100.0%**	**--**
**Random effects model**			**0.00**	**[0.00; 0.02]**	**--**	**100.0%**

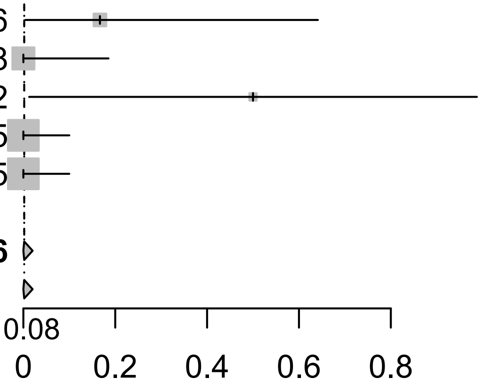

Heterogeneity: $I^2 = 53\%$, $\tau^2 < 0.0001$, $p = 0.08$

Rickettsia raoultii

Study	Events	Total	Proportion	95%−CI	Weight (common)	Weight (random)
Cheng et al. (2019)	32	211	0.15	[0.11; 0.21]	10.4%	9.1%
Cheng et al. (2016)	9	55	0.16	[0.08; 0.29]	2.7%	8.5%
Cheng et al. (2017)	32	308	0.10	[0.07; 0.14]	15.2%	9.2%
Gao et al. (2022)	0	242	0.00	[0.00; 0.02]	11.9%	9.2%
Han et al. (2019)	1	18	0.06	[0.00; 0.27]	0.9%	7.0%
Igolkina et al. (2018)	10	439	0.02	[0.01; 0.04]	21.6%	9.3%
Liu et al. (2016)	0	412	0.00	[0.00; 0.01]	20.3%	9.3%
Lu et al. (2021)	0	6	0.00	[0.00; 0.46]	0.3%	4.6%
Shao et al. (2020)	0	106	0.00	[0.00; 0.03]	5.2%	8.9%
Shao et al. (2019)	0	106	0.00	[0.00; 0.03]	5.2%	8.9%
Shpynov et al. (2009)	6	20	0.30	[0.12; 0.54]	1.0%	7.2%
Yang et al. (2016)	20	106	0.19	[0.12; 0.28]	5.2%	8.9%
**Common effect model**		**2029**	**0.03**	**[0.02; 0.04]**	**100.0%**	**--**
**Random effects model**			**0.05**	**[0.01; 0.11]**	**--**	**100.0%**

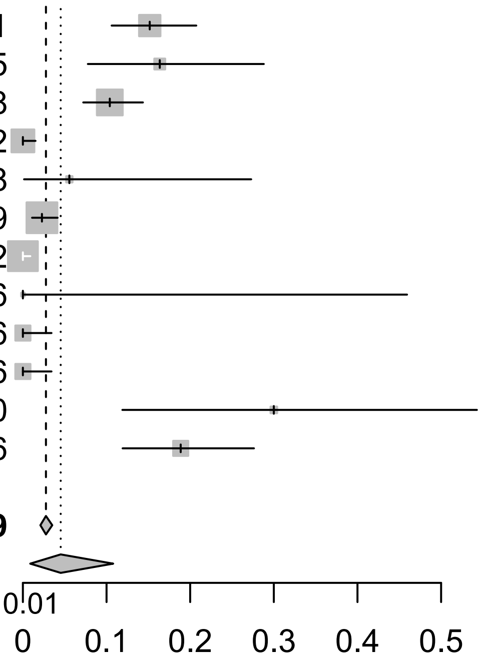

Heterogeneity: I^2 = 95%, τ^2 = 0.0402, p < 0.01

Rickettsia sibirica

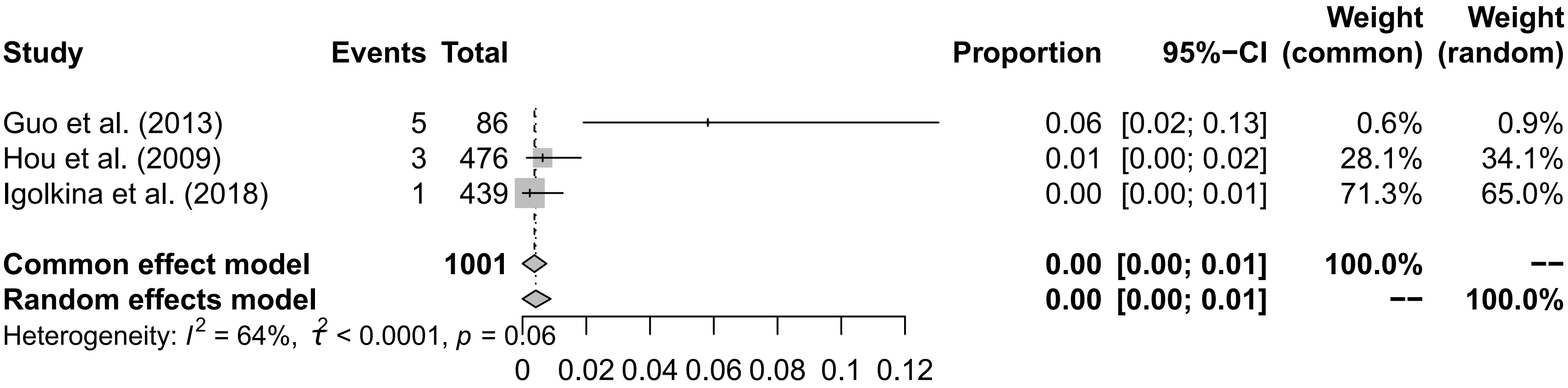

Candidatus Rickettsia kotlanii

Study	Events	Total	Proportion	95%−CI	Weight (common)	Weight (random)
Hornok et al. (2010)	1	257	0.00	[0.00; 0.02]	75.9%	75.9%
Lancz et al. (2006)	2	204	0.01	[0.00; 0.03]	24.1%	24.1%
**Common effect model**		**461**	**0.01**	**[0.00; 0.01]**	**100.0%**	**--**
**Random effects model**			**0.01**	**[0.00; 0.01]**	**--**	**100.0%**

Heterogeneity: $I^2 = 0\%$, $\tau^2 = 0$, $p = 0.46$

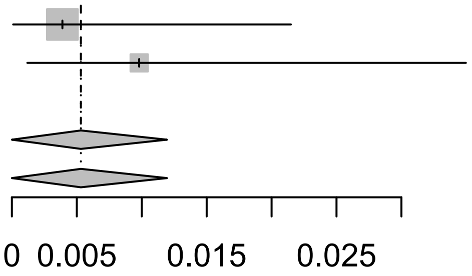

Candidatus Rickettsia tarasevichiae

Study	Events	Total	Proportion	95%−CI	Weight (common)	Weight (random)
Cheng et al. (2019)	4	75	0.05	[0.01; 0.13]	2.8%	6.6%
Cheng et al. (2016)	4	55	0.07	[0.02; 0.18]	2.0%	6.4%
Cheng et al. (2017)	4	308	0.01	[0.00; 0.03]	11.3%	7.0%
Cui et al. (2021)	23	271	0.08	[0.05; 0.12]	10.0%	7.0%
Gao et al. (2022)	23	242	0.10	[0.06; 0.14]	8.9%	7.0%
Igolkina et al. (2018)	14	439	0.03	[0.02; 0.05]	16.1%	7.1%
Li et al. (2019)	0	86	0.00	[0.00; 0.04]	3.2%	6.7%
Li et al. (2022)	0	152	0.00	[0.00; 0.02]	5.6%	6.9%
Liu et al. (2017)	1	412	0.00	[0.00; 0.01]	15.1%	7.1%
Liu et al. (2016)	1	412	0.00	[0.00; 0.01]	15.1%	7.1%
Lu et al. (2021)	0	6	0.00	[0.00; 0.46]	0.2%	3.5%
Shao et al. (2020)	0	106	0.00	[0.00; 0.03]	3.9%	6.8%
Shao et al. (2019)	0	106	0.00	[0.00; 0.03]	3.9%	6.8%
Wei et al. (2017)	3	24	0.12	[0.03; 0.32]	0.9%	5.6%
Zhou et al. (2017)	10	22	0.45	[0.24; 0.68]	0.8%	5.5%
Zhou et al. (2017)	3	5	0.60	[0.15; 0.95]	0.2%	3.1%
**Common effect model**		**2721**	**0.02**	**[0.01; 0.02]**	**100.0%**	**--**
**Random effects model**			**0.04**	**[0.01; 0.08]**	**--**	**100.0%**

Heterogeneity: $I^2 = 91\%$, $\tau^2 = 0.0387$, $p < 0.01$

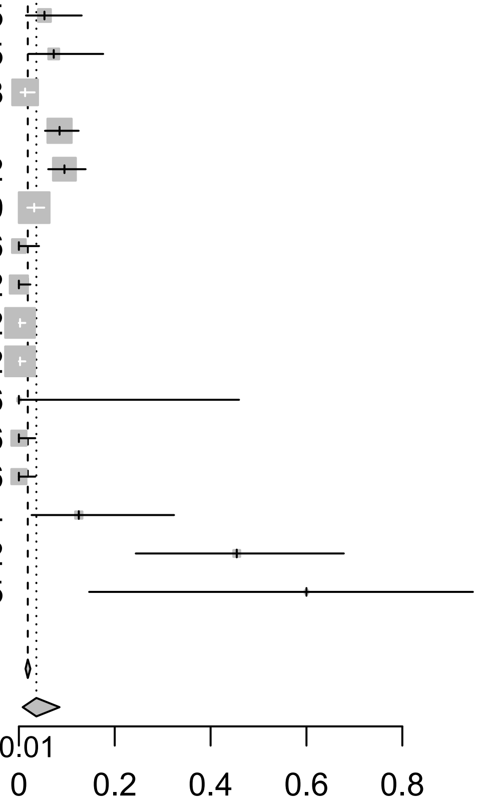

Uncharacterised *Rickettsia*

Study	Events	Total	Proportion	95%−CI	Weight (common)	Weight (random)
Barandika et al. (2008)	0	52	0.00	[0.00; 0.07]	0.9%	2.8%
Chen et al. (2022)	6	98	0.06	[0.02; 0.13]	1.7%	2.9%
Cheng et al. (2019)	17	151	0.11	[0.07; 0.17]	2.5%	3.0%
Cheng et al. (2015)	9	49	0.18	[0.09; 0.32]	0.8%	2.8%
Cheng et al. (2015)	1	34	0.03	[0.00; 0.15]	0.6%	2.7%
Han et al. (2015)	31	781	0.04	[0.03; 0.06]	13.2%	3.1%
Li et al. (2019)	24	827	0.03	[0.02; 0.04]	14.0%	3.1%
Li et al. (2019)	1	5	0.20	[0.01; 0.72]	0.1%	1.6%
Li et al. (2019)	0	14	0.00	[0.00; 0.23]	0.2%	2.3%
Li et al. (2019)	0	2	0.00	[0.00; 0.84]	0.0%	0.9%
Li et al. (2022)	0	152	0.00	[0.00; 0.02]	2.6%	3.0%
Liang et al. (2021)	285	1359	0.21	[0.19; 0.23]	22.9%	3.1%
Liu et al. (2021)	294	442	0.67	[0.62; 0.71]	7.5%	3.0%
Pan et al. (2021)	5	47	0.11	[0.04; 0.23]	0.8%	2.8%
Tang et al. (2012)	31	409	0.08	[0.05; 0.11]	6.9%	3.0%
Tang et al. (2012)	13	108	0.12	[0.07; 0.20]	1.8%	2.9%
Wu et al. (2012)	6	94	0.06	[0.02; 0.13]	1.6%	2.9%
Ye et al. (2018)	0	236	0.00	[0.00; 0.02]	4.0%	3.0%
Borsan et al. (2021)	1	6	0.17	[0.00; 0.64]	0.1%	1.7%
Andersson et al. (2018)	0	1	0.00	[0.00; 0.97]	0.0%	0.5%
Cerro et al. (2022)	0	53	0.00	[0.00; 0.07]	0.9%	2.8%
Chen et al. (2016)	8	12	0.67	[0.35; 0.90]	0.2%	2.2%
Chen et al. (2016)	10	30	0.33	[0.17; 0.53]	0.5%	2.6%
Fu et al. (2015)	5	38	0.13	[0.04; 0.28]	0.6%	2.7%
Guan et al. (2019)	2	28	0.07	[0.01; 0.24]	0.5%	2.6%
Latrofa et al. (2017)	0	6	0.00	[0.00; 0.46]	0.1%	1.7%
Leschnik et al. (2012)	2	30	0.07	[0.01; 0.22]	0.5%	2.6%
Min et al. (2022)	0	48	0.00	[0.00; 0.07]	0.8%	2.8%
Minichova et al. (2017)	0	61	0.00	[0.00; 0.06]	1.0%	2.8%
Polat et al. (2021)	6	63	0.10	[0.04; 0.20]	1.1%	2.9%
Raileanu et al. (2022)	0	88	0.00	[0.00; 0.04]	1.5%	2.9%
Rataud et al. (2022)	2	11	0.18	[0.02; 0.52]	0.2%	2.1%
Sandor et al. (2017)	1	9	0.11	[0.00; 0.48]	0.2%	2.0%
Sonnberge et al. (2022)	3	5	0.60	[0.15; 0.95]	0.1%	1.6%
Spitalska et al. (2021)	0	1	0.00	[0.00; 0.97]	0.0%	0.5%
Svehlova et al. (2014)	0	4	0.00	[0.00; 0.60]	0.1%	1.4%
Szarek et al. (2021)	2	427	0.00	[0.00; 0.02]	7.2%	3.0%
Vichova et al. (2018)	2	36	0.06	[0.01; 0.19]	0.6%	2.7%
Wang et al. (2021)	5	47	0.11	[0.04; 0.23]	0.8%	2.8%
Zhou et al. (2017)	0	4	0.00	[0.00; 0.60]	0.1%	1.4%
Han et al. (2016)	4	55	0.07	[0.02; 0.18]	0.9%	2.8%
**Common effect model**		**5923**	**0.09**	**[0.08; 0.10]**	**100.0%**	**--**
**Random effects model**			**0.07**	**[0.03; 0.11]**	**--**	**100.0%**

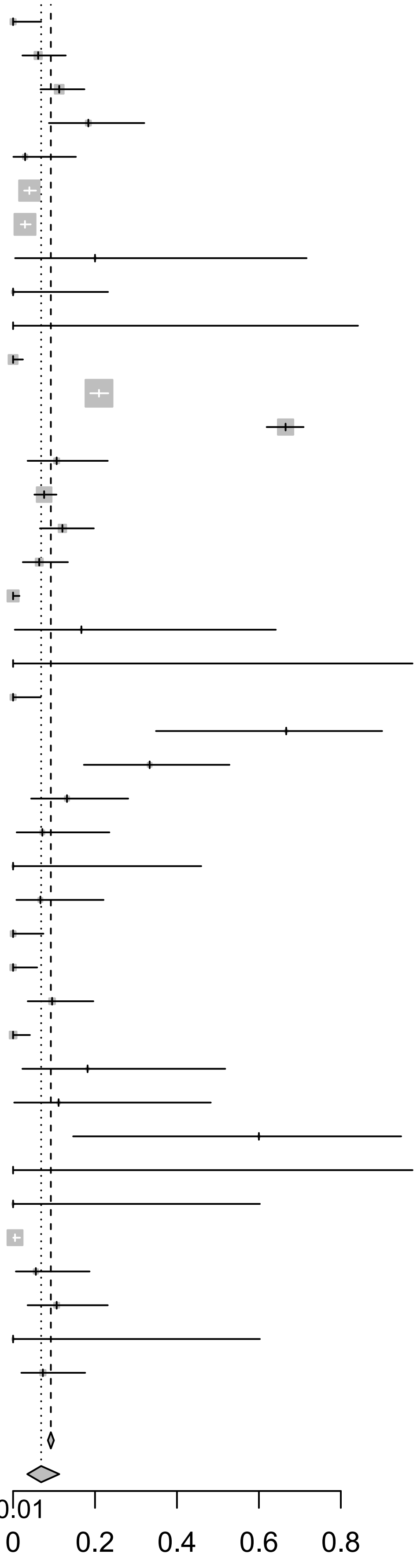

Heterogeneity: $I^2 = 97\%$, $\tau^2 = 0.0511$, $p < 0.01$

Uncharacterised *Theileria*

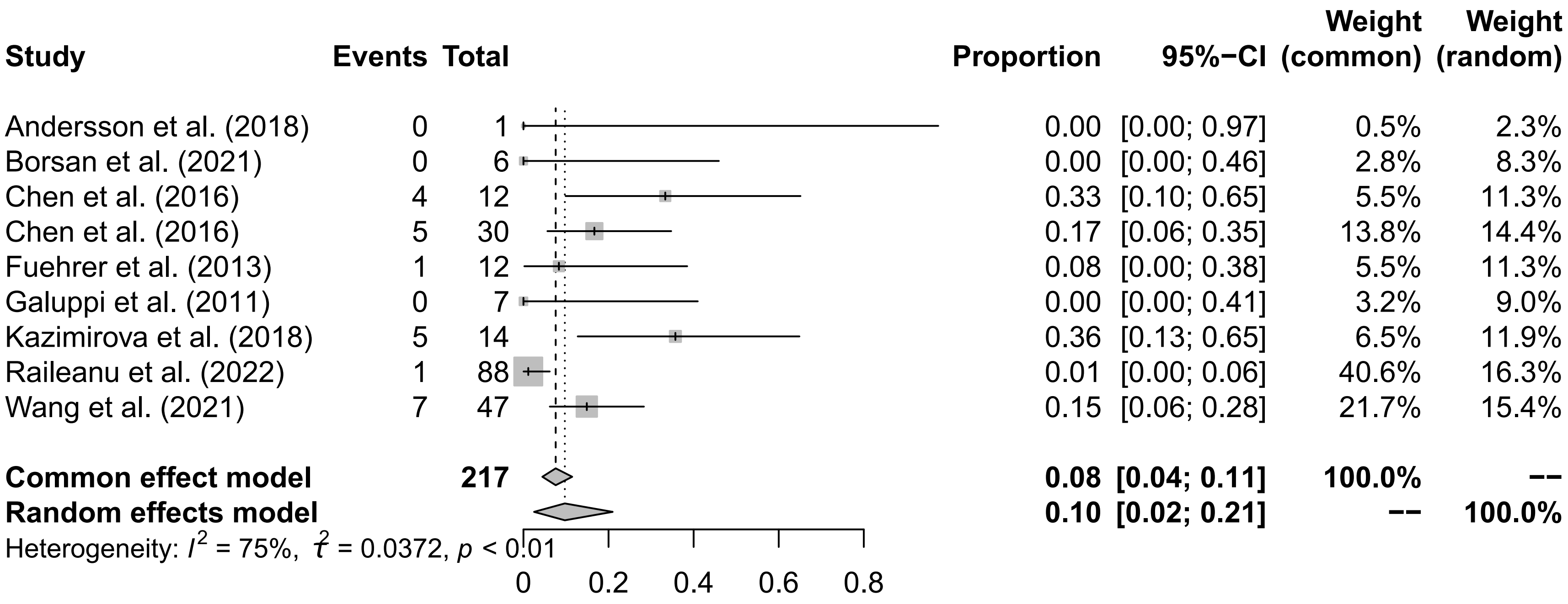

Alongshan virus

Study	Events	Total	Proportion	95%−CI	Weight (common)	Weight (random)
Cai et al. (2020)	0	97	0.00	[0.00; 0.04]	4.5%	4.5%
Feng et al. (2021)	1	1930	0.00	[0.00; 0.00]	90.0%	89.9%
Kholodilov et al. (2021)	2	16	0.12	[0.02; 0.38]	0.7%	0.7%
Li et al. (2022)	0	55	0.00	[0.00; 0.06]	2.6%	2.6%
Wang et al. (2021)	0	47	0.00	[0.00; 0.08]	2.2%	2.2%
**Common effect model**		**2145**	**0.00**	**[0.00; 0.00]**	**100.0%**	**--**
**Random effects model**			**0.00**	**[0.00; 0.00]**	**--**	**100.0%**

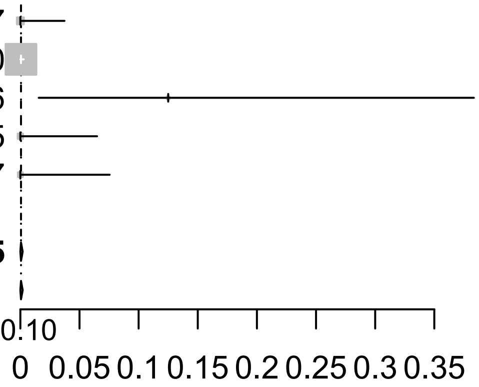

Heterogeneity: $I^2 = 48\%$, $\tau^2 < 0.0001$, $p = 0.10$

Crimean-Congo hemorrhagic fever virus

Study	Events	Total	Proportion	95%−CI	Weight (common)	Weight (random)
Fu et al. (2015)	0	38	0.00	[0.00; 0.09]	99.6%	83.2%
Tekin et al. (2011)	1	3	0.33	[0.01; 0.91]	0.4%	16.8%
**Common effect model**		**41**	**0.00**	**[0.00; 0.04]**	**100.0%**	**--**
**Random effects model**			**0.06**	**[0.00; 0.30]**	**--**	**100.0%**

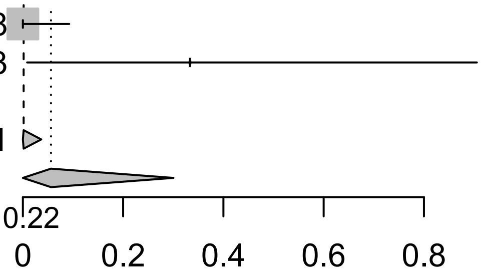

Heterogeneity: $I^2 = 33\%$, $\tau^2 = 0.0184$, $p = 0.22$

Severe fever with thrombocytopenia syndrome virus

Study	Events	Total	Proportion	95%−CI	Weight (common)	Weight (random)
Feng et al. (2021)	0	1930	0.00	[0.00; 0.00]	95.9%	18.6%
Li et al. (2017)	9	104	0.09	[0.04; 0.16]	0.0%	10.0%
Li et al. (2019)	5	86	0.06	[0.02; 0.13]	0.0%	10.8%
Li et al. (2022)	11	152	0.07	[0.04; 0.13]	0.0%	12.4%
Li et al. (2020)	0	366	0.00	[0.00; 0.01]	3.5%	18.5%
Meng et al. (2015)	3	72	0.04	[0.01; 0.12]	0.0%	11.5%
Tian et al. (2017)	17	860	0.02	[0.01; 0.03]	0.6%	18.1%
**Common effect model**		**3570**	**0.00**	**[0.00; 0.00]**	**100.0%**	**--**
**Random effects model**			**0.03**	**[0.01; 0.06]**	**--**	**100.0%**

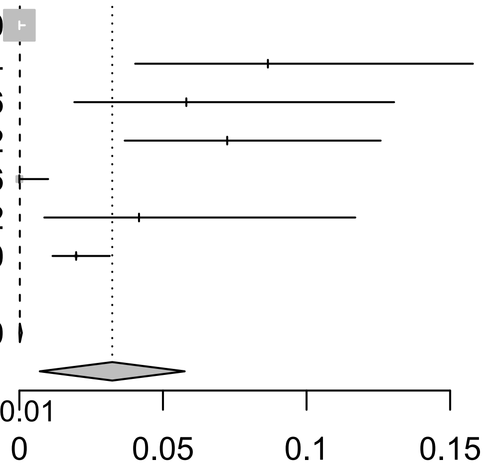

Heterogeneity: $I^2 = 87\%$, $\tau^2 = 0.0009$, $p < 0.01$

South Bay virus

Study	Events	Total	Proportion	95%−CI	Weight (common)	Weight (random)
Ma et al. (2017)	2	464	0.00	[0.00; 0.02]	95.9%	95.9%
Wang et al. (2021)	0	47	0.00	[0.00; 0.08]	4.1%	4.1%
**Common effect model**		**511**	**0.00**	**[0.00; 0.01]**	**100.0%**	**--**
**Random effects model**			**0.00**	**[0.00; 0.01]**	**--**	**100.0%**

Heterogeneity: $I^2 = 0\%$, $\tau^2 = 0$, $p = 0.77$

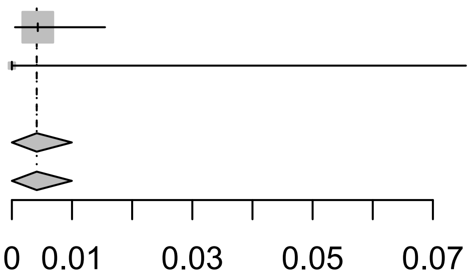

Tick borne encephalitis virus

Study	Events	Total	Proportion	95%−CI	Weight (common)	Weight (random)
Andersson et al. (2018)	0	1	0.00	[0.00; 0.97]	0.0%	0.1%
Cai et al. (2020)	0	97	0.00	[0.00; 0.04]	1.3%	4.8%
Cai et al. (1995)	8	2185	0.00	[0.00; 0.01]	28.9%	7.9%
Dobler et al. (2019)	0	14	0.00	[0.00; 0.23]	0.2%	1.4%
Feng et al. (2021)	0	1930	0.00	[0.00; 0.00]	25.5%	7.9%
Fu et al. (2015)	0	38	0.00	[0.00; 0.09]	0.5%	3.0%
Han et al. (2016)	0	55	0.00	[0.00; 0.06]	0.7%	3.7%
Hayasaka et al. (1999)	0	120	0.00	[0.00; 0.03]	1.6%	5.3%
Hou et al. (2009)	0	476	0.00	[0.00; 0.01]	6.3%	7.2%
Li et al. (2022)	0	55	0.00	[0.00; 0.06]	0.7%	3.7%
Ma et al. (2017)	0	464	0.00	[0.00; 0.01]	6.1%	7.2%
Pinter et al. (2013)	0	489	0.00	[0.00; 0.01]	6.5%	7.2%
Pukhovskaya et al. (2018)	28	498	0.06	[0.04; 0.08]	6.6%	7.2%
Ren et al. (1994)	0	270	0.00	[0.00; 0.01]	3.6%	6.6%
Shchuchinova et al. (2015)	1	132	0.01	[0.00; 0.04]	1.7%	5.4%
Wang et al. (1995)	0	270	0.00	[0.00; 0.01]	3.6%	6.6%
Wang et al. (2021)	0	99	0.00	[0.00; 0.04]	1.3%	4.9%
Wang et al. (2020)	0	327	0.00	[0.00; 0.01]	4.3%	6.8%
Ye et al. (2018)	0	41	0.00	[0.00; 0.09]	0.5%	3.1%
**Common effect model**		**7561**	**0.00**	**[0.00; 0.00]**	**100.0%**	**--**
**Random effects model**			**0.00**	**[0.00; 0.00]**	**--**	**100.0%**

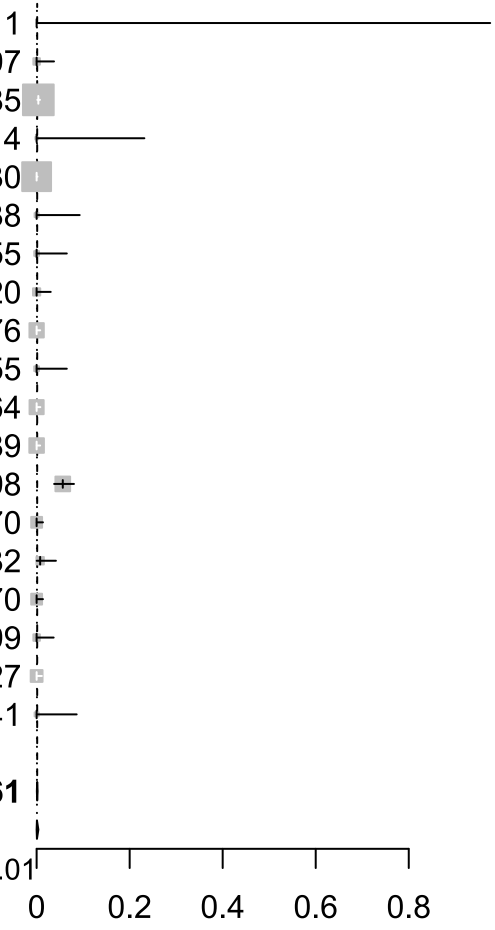

Heterogeneity: $I^2 = 84\%$, $\tau^2 = 0.0037$, $p < 0.01$

Uncharacterised Orthobunyavirus

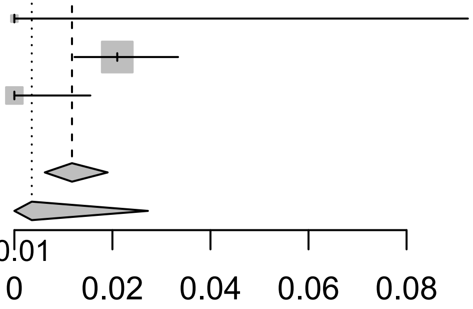

Study	Events	Total	Proportion	95%−CI	Weight (common)	Weight (random)
Fu et al. (2015)	0	38	0.00	[0.00; 0.09]	3.5%	21.9%
Hu et al. (2015)	17	810	0.02	[0.01; 0.03]	74.7%	41.0%
Ye et al. (2018)	0	236	0.00	[0.00; 0.02]	21.8%	37.1%
**Common effect model**		**1084**	**0.01**	**[0.01; 0.02]**	**100.0%**	**--**
**Random effects model**			**0.00**	**[0.00; 0.03]**	**--**	**100.0%**

Heterogeneity: $I^2 = 88\%$, $\tau^2 = 0.0068$, $p < 0.01$
